# Chemoenzymatic Synthesis of Norisoprenoid Aroma Compounds via C–H Activation by Engineered P450_BM3_

**DOI:** 10.1021/acscatal.5c08132

**Published:** 2026-02-27

**Authors:** Wenyu Chen, Rory Woodhouse, Yuan Zhang, Avinash Pandreka, Yang Cao, Linxue Feng, Luet L. Wong

**Affiliations:** † Department of Chemistry, 6396 University of Oxford, Inorganic Chemistry Laboratory, South Parks Road, Oxford OX1 3QR, U.K.; ‡ 665886 Oxford Suzhou Centre for Advanced Research, Ruo Shui Road, Suzhou Industrial Park, Jiangsu 215123, P. R. China

**Keywords:** cytochrome P450, CYP, norisoprenoids, rose ketones, ionone, damascenone, protein engineering, monooxygenases

## Abstract

Norisoprenoid compounds such as the damascones, damascenones, and megastigmatrienones are widely used in the flavor and fragrance industry. Their low natural abundance and the limitations of traditional synthetic routes, such as high energy demands, use of toxic reagents, and challenges in isomeric selectivity, hinder production under mild conditions. Here, we report chemoenzymatic synthesis routes to these compounds using engineered P450_BM3_ variants for late-stage C–H activation. Screening of α- and β-damascone with a panel of 96 P450_BM3_ variants revealed high conversion rates and regioselectivities for the intermediates for acid-catalyzed dehydration to form γ- and β-damascenone, respectively. Megastigmatriene oxidation did not give tabanone due to rearrangement to β-ionol, but alternative routes via α-ionol and α-ionone oxidation yielded a mixture of tabanone isomers. Beyond allylic oxidation, the enzyme collection also oxidized these norisoprenoids at the less reactive sp^3^ aliphatic positions, expanding the diversity of accessible products. Scalability of enzymatic oxidation was demonstrated by the high titer (7.3 g/L), conversion (95%), and total turnover number (9500) for β-damascone oxidation. The findings demonstrate the power of chemoenzymatic strategies in accessing complex norisoprenoids in fewer steps than chemical synthesis routes and lay the groundwork for scalable biotechnological production processes.

## Graphical abstract

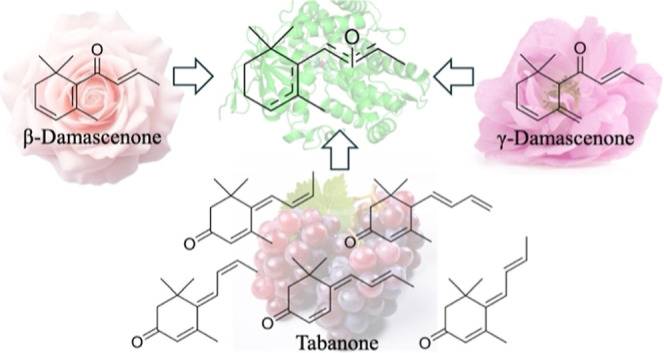

## 1 Introduction

Terpenes and terpenoids are common constituents of essential oils. Norisoprenoids are a subclass of terpenes and possess desirable organoleptic and medicinal properties. Many norisoprenoids and their alcohol, aldehyde, and ketone derivatives are biologically active, e.g., damascones and ionones (Figure 1), and have been identified as a novel class of cancer chemopreventive phytochemicals.^1^ In addition to their bioactivities, norisoprenoid compounds have numerous applications in perfumery and the food industry. Because of their closely related structures, norisoprenoids have overlapping aroma profiles. α-Irone, found in iris oil, has a sweet floral, iris, and woody odor and is one of the major odorants in perfumes. Extracted from Bulgarian roses, β-damascenone is a minor component of rose oil but a major contributor to the rose aroma, with an odor threshold of 0.002 ppb, while adding sweet subnotes of ripe plums and berries, followed by a tobacco nuance as the concentration drops. γ-Damascenone also possesses a rose-like note but has more apple and citrus undertones. Megastigmatrienone, also known as tabanone, was quantified in wines.^2^ While its prominent tobacco note has found applications in food and other applications, the balsamic, fruity, and spicy notes have made it a popular component in household air care products.

**Figure 1:**
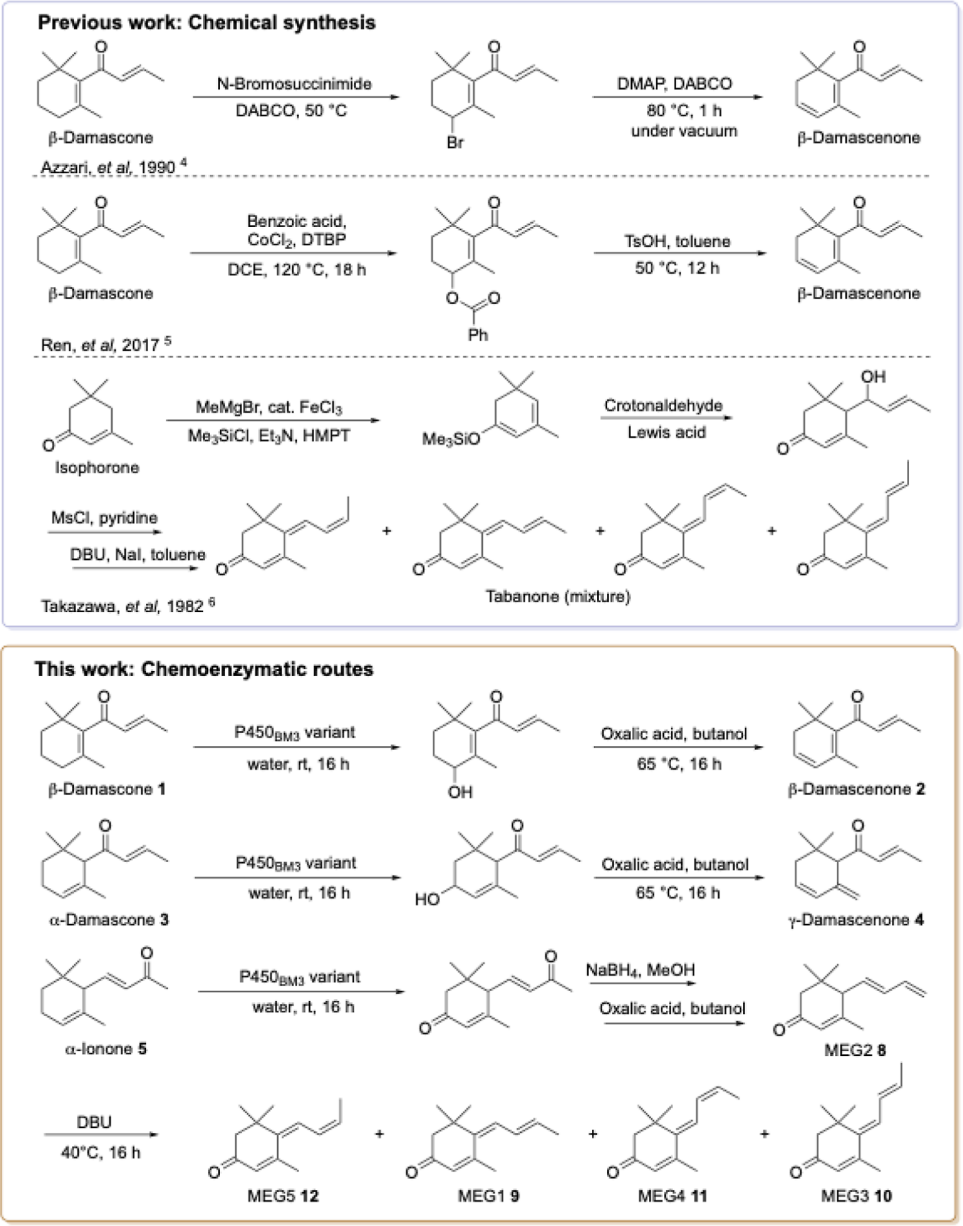
Chemical synthesis routes of the damascenones from damascones and a mixture of megastigmatrienones from isophorone and chemoenzymatic routes developed in this work.

The complex and multifaceted olfactory properties of norisoprenoid compounds have made them valuable products, but they are difficult to extract from nature due to their low concentration. β-Damascenone is biosynthesized via the degradation of carotenoids which are converted to neoxanthin, the proposed precursor to β-damascenone, by oxidases.^3^ Chemically, the oxidation of β-damascone is an attractive route to β-damascenone. Allylic bromination at C4 of β-damascone followed by dehydrobromination in the presence of DABCO/DMAP gives β-damascenone.^4^ Another route is cobalt-catalyzed oxidative esterification at C4 at high temperature followed by acid-catalyzed elimination of benzoic acid (Figure 1).^5^ The origin of the five isomers of megastigmatrienone is less well-known. It has been proposed that carotenoid degradation leads to the formation of 3-oxo-α-ionol, which may be the precursor to megastigmatrienone via acid-catalyzed dehydration. Due to difficulties in controlling the selectivity for the five isomers, only a few synthetic methods have been reported; one efficient route starts from isophorone (Figure 1).^6^ The aim of this work is to develop chemoenzymatic routes to β-damascenone and megastigmatrienones initiated by P450_BM3_-catalyzed C–H bond oxidation. P450_BM3_ is highly attractive for biotransformation applications as it is a single-polypeptide, self-sufficient enzyme which has been expressed to a high level in *E. coli* fermentations and multikilogram scale reactions have been reported.^7,8^ β-Damascenone (**2**) can be prepared from β-damascone via oxidation to the C4 alcohol followed by acid treatment to eliminate water, likewise for the synthesis of γ-damascenone (**4**) from α-damascone (**3**) which mirrors the biosynthetic route in grapes. We propose three pathways for the synthesis of megastigmatrienone (tabanone). Acid treatment of β-ionol gives megastigmatriene **7** that is oxidized at C3 to give megastigmatrienone. Alternatively, oxidation of α-ionone (**6**) at C3 to 3-oxo-α-ionone, selective reduction of the side chain ketone to the C9 alcohol followed by acid treatment gives the trienones **8**–**12**. The third approach is the oxidation of α-ionol (**5**) to 3-oxo-α-ionol, followed by acid treatment. Oxidation of these target substrates to the C3 ketone can be accomplished directly by sequential oxidation by a P450_BM3_ enzyme or the C3 alcohol from P450-catalyzed oxidation is oxidized to the ketone by an alcohol dehydrogenase.

Cytochrome P450 (CYP) enzymes have been reported to catalyze the oxidation of norisoprenoids to generate potential precursors for synthesis.^9–12^ CYP101B1 from *Novosphingobium aromaticivorans* DSM12444 catalyzed C3 oxidation of α-ionone and β-damascone with high selectivity.^13,14^ CYP260B1 and CYP267B1 from *Sorangium cellulosum* So ce56 catalyzed the selective oxidation of carotenoid-derived compounds such as the ionones and damascones.^15^ CYP109E1 from *Bacillus megaterium* has been found to oxidize terpenoid compounds.^16^ Unspecific peroxygenases (UPOs) catalyze the formation of norisoprenoid alcohols, aldehydes, and carboxylic acids.^17–19^ Herein, we report the oxidative diversification of β-damascone (**1**), α-damascone (**3**), α-ionol (**5**), α-ionone (**6**), and megastigmatriene (**7**) by engineered P450_BM3_ and the development of chemoenzymatic routes to β-damascenone (**2**), γ-damascenone (**4**), and tabanone (megastigmatrienones **8**–**12**). Preliminary studies showed that wild-type (WT) P450_BM3_ had no detectable activity with substrates **1**, **5**, **6**, and **7** and <5% conversion of substrate **3**. These substrates were then screened for oxidation by a panel of 96 P450_BM3_ variants generated in previous work.^20–24^ This panel was a subset of a collection of over 1000 variants constructed by site-directed mutagenesis to target 2–7 key residues around the substrate binding pocket to provide variants with diverse substrate pocket topologies for screening for the oxidation of unnatural substrates. Subsets of this collection have demonstrated efficacy with a broad range of substrates, including steroids, cyclic nitrogen compounds, terpenes, etc. The 96-enzyme panel contained variants with and without a mutation of Phe87 (F87A, F87V, and F87I), a key active residue located above the heme. The topology of the substrate pocket was varied by additional mutations at residues close to the heme (I263, A264, A328, A330) as well as residues that were further removed, in the B′ helix (S72, A74, V78, F81, A82), in the F helix (L181, A184, L188), and at the entrance to the substrate access channel (R47, Y51) (Figure 2). Preparative scale reactions were conducted with variants showing high activity for product characterization and the production of desired precursors for synthesizing the damascenones and tabanones.

**Figure 2:**
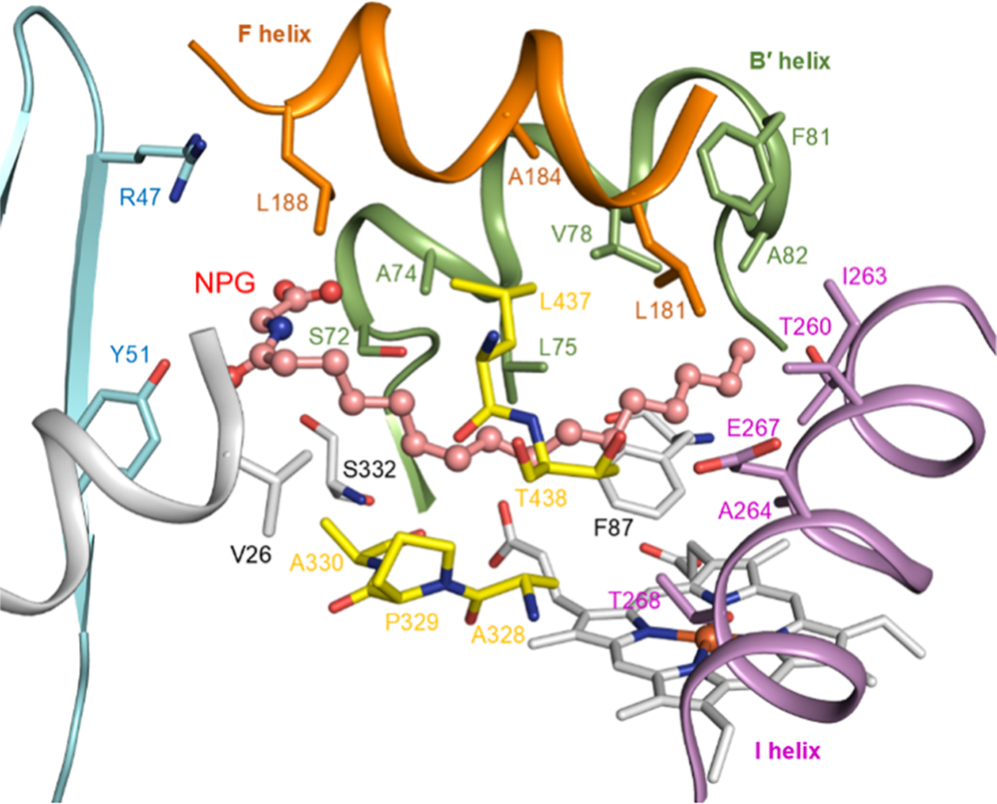
Active site structure of the heme domain of wild-type P450_BM3_ with *N*-palmitoyl-glycine bound (pdb code: 1JPZ) showing the location of residues targeted in the construction of the panel of 96 enzyme variants (Table S1) selected for screening.

## 2 Results and Discussion

### 2.1 Oxidation of β-Damascone by P450_BM3_

β-Damascone (**1**) was screened for oxidation with the panel of 96 P450_BM3_ enzyme variants (Table S1 in the Electronic Supporting Information, ESI). Of these, 66 showed >50% conversion (5 mM of **1** with 2 μM enzyme; 50% conversion corresponds to a total turnover number, TON, of 1250) to a collection of five major products. Full data on activity and selectivity are given in Table S2. The products were purified from preparative scale reactions by silica column chromatography and characterized by their NMR and MS data (full characterization data in the ESI) as 2-hydroxy-β-damascone (**1a**), 3-hydroxy-β-damascone (**1b**), 4-hydroxy-β-damascone (**1c**), 4-oxo-β-damascone (**1d**), and 10-hydroxy-β-damascone (**1e**) (Figure 3 and Table 1). The NMR data for **1b**, **1c**, **1d**, and **1e** were consistent with literature reports.^14,17,25,26^ Product **1a** was a new metabolite of β-damascone; **1a** could not be separated from **1e** by column chromatography, but its structure was readily assigned by 1D- and 2D-NMR data. The wider range of oxidation products observed with the P450_BM3_ screening panel compared with other P450 enzymes studied previously might arise from the diverse substrate pocket topology generated by the combinations of mutations in the enzyme collection.

**Figure 3:**
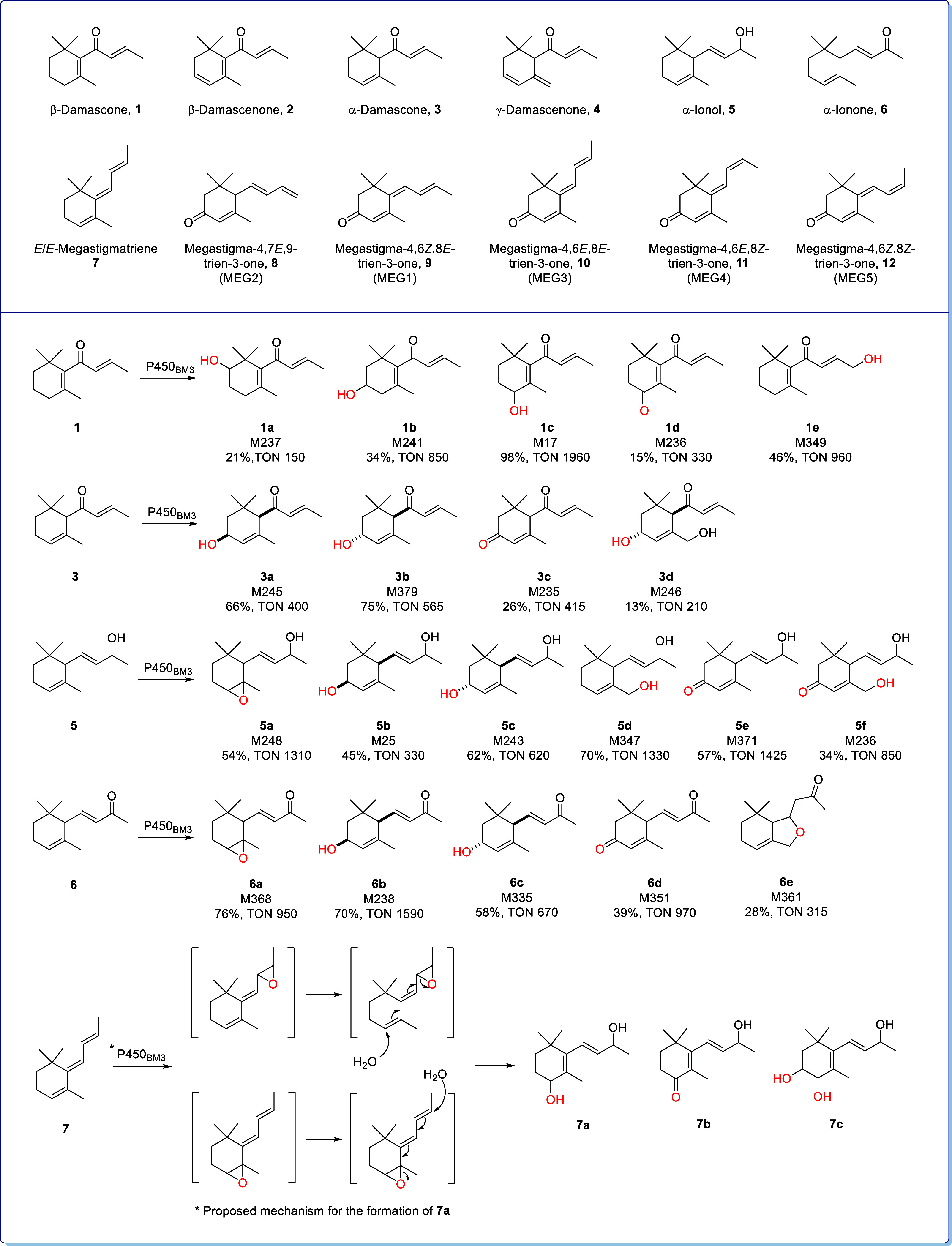
Oxidation products of β-damascone (**1**), α-damascone (**3**), α-ionol (**5**), α-ionone (**6**), and megastigmatriene (**7**), and the proposed mechanism for the formation of **7a** from **7**. The P450_BM3_ variant with the highest selectivity for the formation of each product is also shown. Using product **1a** as an example, variant M237 is the most selective of the 96 screened variants for the formation of **1a** from the oxidation of **1**, with 21% of **1a** in the product mixture, and TON refers to the turnover number for the formation of **1a** in the reaction.

**Table 1:**
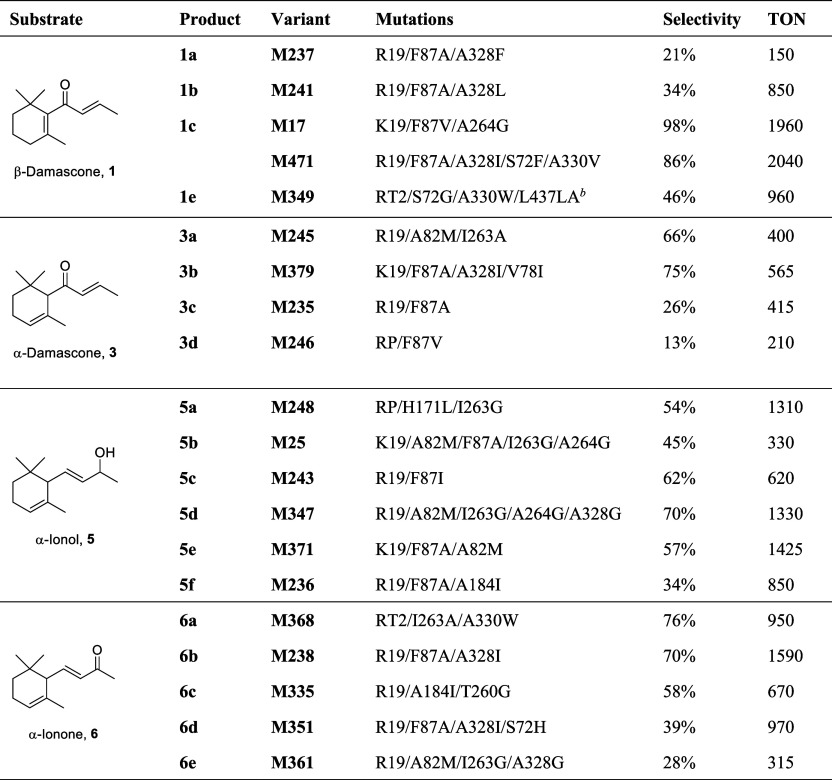
Activity and Selectivity for the Oxidation of β-Damascone (**1**), α-Damascone (**3**), α-Ionol (**5**), and α-Ionone (**6**)­^a^

a The P450_BM3_ variant with the highest selectivity for the formation of each characterized product from the oxidation of the norisoprenoid substrates is shown. Screening scale reactions (0.5 mL in 24-well plates) were in 200 mM phosphate buffer, pH 8.0, containing 2 μM P450_BM3_ enzyme, 5 mM β-damascone (**1**), α-damascone (**3**), α-ionol (**5**), α-ionone (**6**), 40 μM NADP^+^, 100 mM glucose, and 4 U/mL GDH. Plates were shaken at 120 rpm at 20 °C for 16 h. Selectivity refers to the percentage of the oxidation product in the product mixture from the reaction catalyzed by the enzyme variant. TON refers to the turnover number for the formation of the product for each variant.

b L437LA denotes the insertion of an alanine residue after Leu437. K19 = H171L/Q307H/N319Y; R19 = R47L/Y51F/K19; RP = R47L/Y51F/I401P; RT2 = R47L/Y51F/A191T/N239H/I259V/A276T/L353I; KU3 = N239H/I259V/A276T.

Of the 96 variants, 82 gave 4-hydroxy-β-damascone (**1c**), the proposed precursor to β-damascenone, as the major product (>50%). The K19/F87V/A264G variant formed 98% **1c** with 80% conversion (Table 1, M17; Table S2, M244). Most variants showing higher conversion had lower selectivity for **1c**, but a good compromise between activity and selectivity was observed with the R19/F87A/A328I/S72F/A330V variant (M471, 86% **1c**, 95% conversion, Table 1). Further oxidation of **1c** to ketone **1d** was observed for variants containing the F87A mutation, e.g., R19/F87A/A184I gave 15% of **1d** and 66% of **1c** (M236), but full conversion to **1d** was not observed even in prolonged reactions. Substitutions of A328 with residues with bulkier side chains in F87A-based variants shifted oxidation away from the allylic C4 position toward the unactivated C2 and C3 centers. The A328F mutation increased C2 oxidation to form 21% **1a** (Table 1, M237), whereas the A328L mutation favored 3-hydroxy-β-damascone, **1b** (M241, 34%). Addition of the E267F mutation to R19/F87A/A328I increased C3 oxidation selectivity from 3% to 32% of **1b** (Table S2, M333). 10-Hydroxy-β-damascone (**1e**) was favored by the retention of F87, by the bulky substitutions A330P and A330W, and by the S72W mutation (M231, M256, M257, and M262).

Retention of F87 and introduction of bulky substitutions at A330 were expected to constrain the space above the heme such that the least sterically hindered terminal C10 methyl group was more likely to approach the ferryl oxygen. The L437LA insertion mutation (insertion of an alanine residue between Leu437 and Thr438) limited the space available higher up the substrate pocket, which might force a closer approach of a substrate to the heme. This insertion improved the selectivity for **1e** from 13% to 46% when introduced to the RT2/S72G/A330W variant, almost entirely from reduction of the C4 selectivity from 87% to 52%, while maintaining the conversion at >80% (TON = 960, M349).

These screening results showed varied trends and effects of mutations, some of which suggested new combinations to increase the selectivity for the other products. For example, C3 oxidation percentages could be increased via the introduction of the E267F mutation to R19/F87A/A328L (M241), and C10 selectivity could be increased by adding the L437LA insertion to the RT2/S72W/A330W (M257) and KU3/S72W/A330P (M262) variants.

### 2.2 Oxidation of α-Damascone by P450_BM3_

Screening of α-damascone (**3**) with the panel of 96 variants gave four major products (Table S3) which were characterized as *cis*-3-hydroxy-α-damascone (**3a**), *trans*-3-hydroxy-α-damascone (**3b**), 3-oxo-α-damascone (**3c**), and 3,13-dihydroxy-α-damascone (**3e**) (Figure 3 and Table 1). The relative stereochemistry of C3 and C6 for **3a** and **3b** was determined from NOE data, but the absolute configurations were not established. Variants with the F87A mutation favored *trans*-3-alcohol **3b** (Table S3). R19/F87A/A328I gave 68% of product **3b**, and addition of the V78I mutation raised the selectivity to 75% but lowered the conversion. Variants without a substitution at F87, on the other hand, favored the *cis*-3-alcohol **3a**, but conversions were generally lower (<50%). Variant RP gave 38% **3a** and 20% **3b**, RP/F81W showed higher selectivity for **3a** and higher conversion, and the highest selectivity for **3a** (66%) was observed with the RP/A82M/I263A variant. The further oxidation products **3c** and **3d** were mostly observed with variants that contained the F87A mutation.

### 2.3 Oxidation of α-Ionol by P450_BM3_

Racemic α-ionol (**5**) was screened for oxidation with the panel of 96 P450_BM3_ variants. α-Ionol was a good substrate for the P450_BM3_ variants, with 61 of these showing >50% conversion (Table S4). Preparative scale reactions led to the characterization of seven products; the 4,5-epoxide (**5a**),^1,26,27^ *cis*-3-hydroxy-α-ionol (**5b**), *trans*-3-hydroxy-α-ionol (**5c**), 13-hydroxy-α-ionol (**5d**), 3-oxo-α-ionol (**5e**), 3-oxo-13-hydroxy-α-ionol (**5f**), and 3-oxo-α-ionone (**6d**) (Figure 3 and Table 1).^13,28^ Product **5d** was a new metabolite of α-ionol. Although **6d** was isolated from preparative scale reactions, it was a minor product in screening scale reactions.

Few variants showed high selectivities for *cis*- and *trans*-3-hydroxy-α-ionol. Those showing moderate selectivity contained the F87A/V/I mutation and tended to be less active (Tables 1 and S4, M25, M56, and M243). 3-Oxo-α-ionol (**5e**), the proposed precursor to tabanone and target of the screening, was a minor product for most enzymes in the screening library except for K19/F87A/A82M which gave 57% of **5e** at 100% conversion (M371). The reaction was readily scaled to 400 mg of **5** converted (1 g/L), enabling the isolation of **5e** in a 44% yield. Of the other products, mutations F87V and I263G favored the epoxide **5a** (M7, M17, and M248). 13-Hydroxy-α-ionol (**5d**) was favored by variants containing the A82M/I263G/A264G combination without mutation of F87, e.g., R19/A82M/I263G/A264G/A328G gave 70% of **5d** (M347). Product **5f** was formed from three cycles of ionol oxidation; the most selective variant for **5f** was R19/F87A/A184I (34%, M236).

### 2.4 Oxidation of α-Ionone by P450_BM3_

α-Ionone (**6**) was oxidized by the panel of 96 P450_BM3_ enzymes to five major products—the 4,5-epoxide (**6a**), *cis*-3-hydroxy-α-ionone (**6b**), *trans*-3-hydroxy-α-ionone (**6c**), 3-oxo-α-ionone (**6d**), and the cyclization compound **6e** (Figure 3 and Table 1). This cyclic compound had been observed for β-ionone^18^ but was a new metabolite of α-ionone formed by intramolecular Michael addition of the unobserved C13 alcohol to the side chain αβ-unsaturated ketone. Full activity and selectivity data are provided in Table S5. The relative stereochemistry of C3 and C6 for alcohols **6b** and **6c** was determined from NOE data, but the absolute configurations were not established.

The main target of the screening was selectivity for C3 oxidation to give **6b** and **6c**, preferably direct oxidation to ketone **6d**. As shown in Tables 1 and S5, variants containing the F87A mutation showed ≥90% selectivity for the C3 alcohols **6b** and **6c** together with some **6d** (M235, M238, M333, and M372). Addition of the A328L mutation to R19/F87A raised the selectivity to 70% for the *cis* alcohol **6b** (M241), whereas the A184I/A328I combination slightly favored the *trans* alcohol **6c** (46%, M372). The highest selectivity for **6c** of 58% was observed for the R19/A184I/T260G variant that had an I-helix mutation (T260G), but the F87 residue was unchanged (M335). Further oxidation to ketone **6d** was promoted by the mutations A184I and S72H, with the highest proportion of **6d** reaching 39% (total C3 selectivity = 89%) at 99% conversion (M351). It was notable that different residues and mutations were required to effect selective oxidation at C3 and C13 of α-ionone (**6**) and α-ionol (**5**). These differences might arise from the hydrogen bonding characteristics, whereby the H-bond-donating ionol interacted with different residues to the H-bond-accepting ionone, leading to altered binding orientations and product selectivities.

Since there was only partial conversion of the C3 alcohols **6b** and **6c** to the ketone **6d** in the P450_BM3_-catalyzed reactions, the product mixture from variant K19/F87A/A328I/A184I (M372) was screened with a panel of commercially available alcohol dehydrogenases for conversion to **6d**. One enzyme, CRED231, selectively converted **6c** to **6d** while another, CRED641, was selective for the oxidation of **6b**. A 1:1 mixture of these two enzymes gave >90% conversion of the two C3 alcohols to ketone **6d**. Selective reduction of **6d** with 1 equiv. of NaBH_4_ gave 3-oxo-α-ionol (**5e**), the proposed precursor to megastigmatrienone (Figure 4III-e).

**Figure 4:**
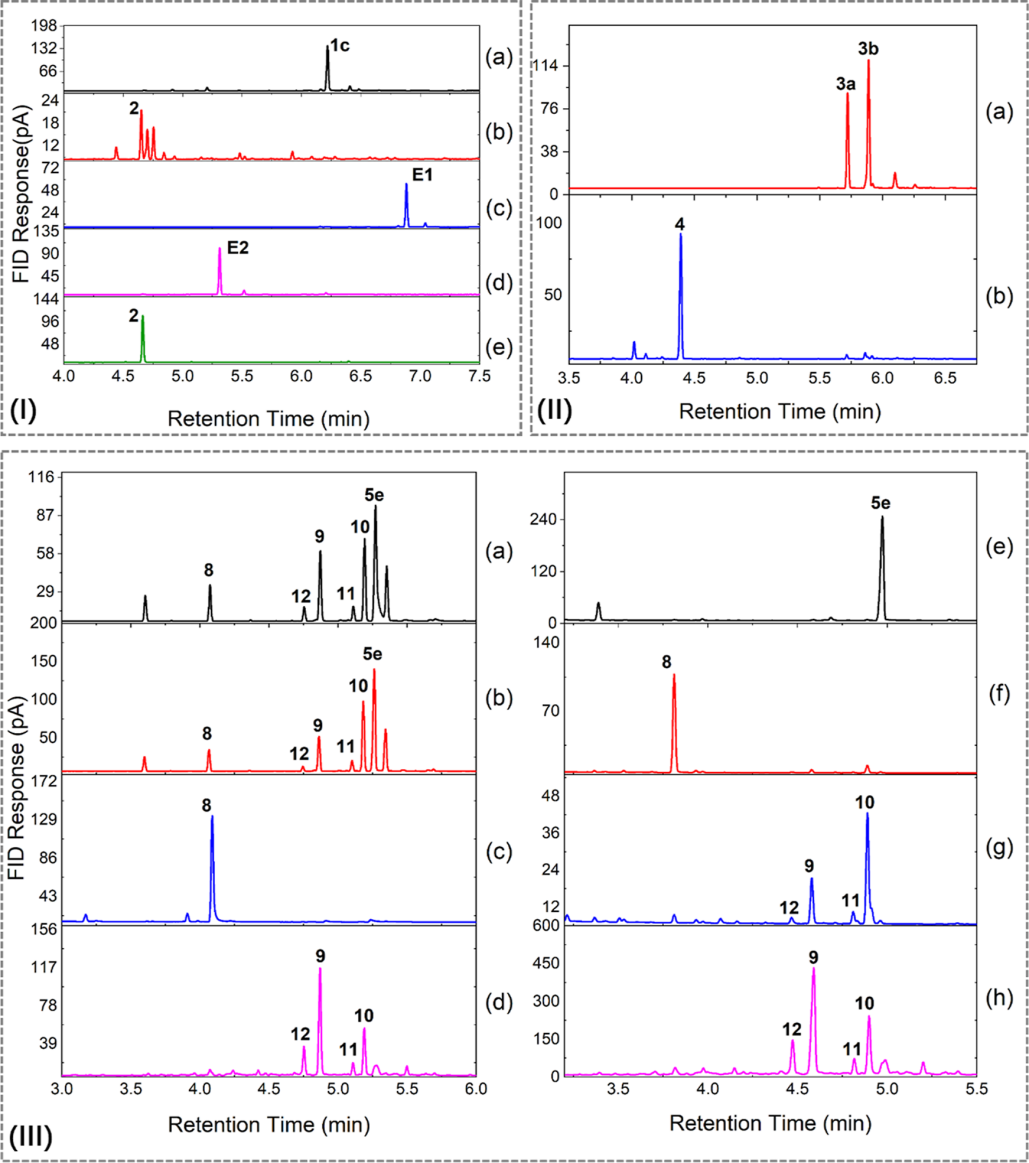
Gas chromatographic (GC) analysis of the synthesis of **2**, **4**, and **8**–**12** via acid-catalyzed dehydration reactions. (I) Synthesis of **2** from **1c**. (a) Control sample of **1c**. (b) **1c** with dilute H_2_SO_4_ (pH = 1) gave a mixture of four products (22% of **2**). (c) **1c** with acetic acid and acetic anhydride formed ester **E1**. (d) **1c** with trifluoracetic acid formed ester **E2**. (e) **1c** with oxalic acid gave full conversion to **2**. (II) Synthesis of **4** by heating a mixture of **3a** and **3b** with oxalic acid. (III) Synthesis of **8**–**12** from **5e**. (a) Heating **5e** with aqueous HCl (67% conversion). (b) Heating **5e** with sulfuric acid. (c) Heating **5e** with oxalic acid gave **8**. (d) Commercial tabanone **9**–**12** as reference (**9**, 50% and **10**, 20%). (e) Reduction of **6d** with NaBH_4_ gave **5e**. (f) Heating **5e** with oxalic acid gave **8**. (g) Treatment of **8** with DBU gave tabanone isomers **9**–**12** (**9**, 23%; **10**, 61%). (h) Commercial tabanone **9**–**12** as reference (**9**, 50% and **10**, 20%).

### 2.5 Oxidation of Megastigmatriene by P450_BM3_

Megastigmatriene (triene, **7**) was synthesized in 84% yield via borohydride reduction of β-ionone followed by treatment with sulfuric acid.^29^ The NMR data (see the ESI) showed that the major isomer was the *E/E* form (93%). Screening of triene **7** with the 96-enzyme variant panel showed that 25 enzymes gave >50% conversion. However, most variants gave complex mixtures of numerous products which could not be separated by silica column chromatography. Megastigmatriene is acid labile, which may lead to the formation of multiple products via rearrangements. Nevertheless, preparative scale reactions led to the characterization of the three major products observed with many variants as 4-hydroxy-β-ionol (**7a**), 4-oxo-β-ionol (**7b**), and 3,4-dihydroxy-β-ionol (**7c**).^30,31^ There was no evidence for significant proportions of the C3 alcohol or ketone, which were expected to have shorter retention times on the GC compared to **7a**–**7c**. Products **7b** and **7c** were likely formed by further oxidation of 4-alcohol **7a**, which could be formed from megastigmatriene **7** via initial epoxidation of the C4,C5 or C8,C9 double bonds followed by epoxide ring opening and trapping of the carbocation by water at either C4 or C9 (Figure 3). The direct oxidation of **7** did not appear to be a viable route to tabanone isomers **8**–**12** and was not pursued further.

### 2.6 Chemoenzymatic Synthesis of β-Damascenone

We explored the scalability of the oxidation of β-damascone (**1**) to give C4 alcohol **1c**, the proposed precursor to β-damascenone (**2**), by increasing the reaction volume and substrate concentration. Catalyst loading was also reduced to raise the TON. Reactions were conducted at a 500 mL scale with 2 μM of the R19/F87A/A328I/S72F/A330V variant (M471) and different β-damascone concentrations: 10 mM (0.96 g of **1**, 0.02% catalyst), 20 mM (1.92 g of **1**, 0.01% catalyst), and 40 mM (3.84 g of **1**, 0.005% catalyst). After 16 h, the reaction mixtures containing 10 mM and 20 mM β-damascone showed >95% conversion, corresponding to a TON > 9500 for substrate conversion at 20 mM of (**1**). The reaction with 40 mM β-damascone achieved 21% conversion (TON = 4200). Increasing the enzyme catalyst loading to 0.01% raised the conversion to 95% with 86% selectivity for **1c** (7.30 g/L **1** converted, TON = 9500), demonstrating the scalability of this enzymatic process. Product **1c** was isolated by silica column chromatography in 67% yield (based on the mass of β-damascone converted) from this reaction.

β-Damascenone (**2**) can be generated from **1c** by acid-catalyzed dehydration. Treatment of **1c** with sulfuric acid at 70 °C for 16 h gave total conversion to 22% of **2** together with three unidentified products (Figure 4I-a,I-b). Screening of other acids revealed that acetic acid formed the acetate derivative **E1** which did not undergo elimination on heating at 90 °C for 2 days (Figure 4I-c), whereas trifluoroacetic acid formed the ester **E2** which eliminated cleanly to form **2** after 2 days at 90 °C (Figure 4I-d,I-e). Oxalic acid gave **2** as the only product without an evident intermediate after 16 h at 65 °C (Figure 4I-e). When 250 mg of **1c** was treated with oxalic acid under these conditions, **2** was isolated in 66% yield after silica column chromatography. This sample of **2** possessed a strong and pleasant rose scent that was cleaner than that of a commercial sample.

### 2.7 Chemoenzymatic Synthesis of γ-Damascenone

The successful synthesis of β-damascenone stimulated interest in the dehydration of 3-hydroxy-α-damascone (**3a**/**3b**). A 10 mg mixture of *cis*-3-hydroxy-α-damascone **3a** and *trans*-3-hydroxy-α-damascone **3b** (4:6 ratio) isolated from a preparative scale reaction with the R19/F87A/A328I variant was treated with oxalic acid at 65 °C in *n*-butanol, leading to full conversion after 16 h (Figure 4II-a,II-b). However, instead of producing α-damascenone via dehydration across C2 and C3, γ-damascenone (**4**) was formed (34% isolated yield) via allylic rearrangement to form the more stabilized C5 carbocation, followed by proton abstraction from the C13 methyl group. This sample of γ-damascenone possessed a fruity and pleasant citrus scent.

### 2.8 Conversion of 3-Oxo-α-ionol to Megastigmatrienone

Treatment of 3-oxo-α-ionol (**5e**) with 6 M hydrochloric acid (80 °C, 2 h) did not lead to full dehydration. A mixture of products (Figure 4III-a) including 11% of the terminal alkene isomer **8** (megastigma-4,7*E*,9-trienone, MEG2), 26% of **10** (6*E*/8*E*-tabanone, MEG3), and 23% of **9** (6*Z*/8*E*-tabanone, MEG1) was formed. Treatment with 3 M sulfuric acid (80 °C, 1 h) in water also gave low conversion to a similar mixture of products (Figure 4III-b). Heating with oxalic acid (80 °C, 1 h) in butanol led to exclusive formation of the terminal alkene isomer **8** (MEG2, Figure 4III-c) (isolated yield 59%). The fully conjugated isomers **9**–**12** were not formed (Figure 4III-d).

Commercial samples of tabanone did not contain MEG2 (**8**), only the fully conjugated isomers (50% of **9**, 20% of **10**, 4% of **11**, and 10% of **12**, Figure 4III-d,h). MEG2, which was not fully conjugated, was likely the kinetic product of the reaction. Therefore, we explored base-catalyzed isomerization of the terminal alkene **8** to the fully conjugated tabanone isomers **9**–**12**. Stirring of **8** with NaOH in aqueous methanol at 40 °C gave 66% conversion to a mixture of 27% **10** and 13% **9** and small amounts of **11** and **12**. Treatment of **8** with DBU in DME at 40 °C led to full conversion (Figure 4III-g) to tabanone isomers **9**–**12** (isolated yield 14%), comprising 23% **9** (6*Z*/8*E*, MEG1), 61% **10** (6*E*/8*E*, MEG3), 5% **11** (6*E*/8*Z*, MEG4), and 4% **12** (6*Z*/8*Z*, MEG5). This mixture had a tobacco-like aroma even though it had a different isomeric composition from a commercial sample of tabanone.

## 3 Conclusion

This study demonstrates the potential of engineered P450 enzymes to catalyze key C–H bond oxidation steps in the chemoenzymatic synthesis of fine chemicals (Figure 5). Chemical synthesis routes, while effective, require multiple steps, harsh conditions, and toxic reagents and often exhibit poor selectivity. Biocatalytic strategies can offer shorter and more sustainable synthetic pathways by leveraging the selectivity of enzymatic reactions. The small panel of 96 P450_BM3_ variants was sufficiently diverse to provide enzymes for the selective formation of the required precursors as well as products from oxidation at unactivated positions. The chemoenzymatic synthesis for β- and γ-damascenone illustrated semisynthesis using a natural feedstock. The sample of tabanone isomers generated via the oxidation of α-ionol and α-ionone gave a more tobacco-like aroma than a commercial sample. Scalability of enzymatic synthesis was demonstrated with β-damascone oxidation, with 7.30 g/L substrate conversion in 16 h and a total turnover number close to 10,000 under unoptimized conditions. The volumetric yield remains a limiting factor due to substrate inhibition at higher concentrations. Further evolution of the best performing variants could enhance the selectivity and total turnover number and reduce substrate inhibition. Enzyme variants with a higher tolerance for organic compounds and methods for in situ product removal will be beneficial for process intensification. In conclusion, this work establishes a foundation for the development of efficient, selective, and environmentally friendly biocatalytic processes for the synthesis of norisoprenoid compounds.

**Figure 5:**
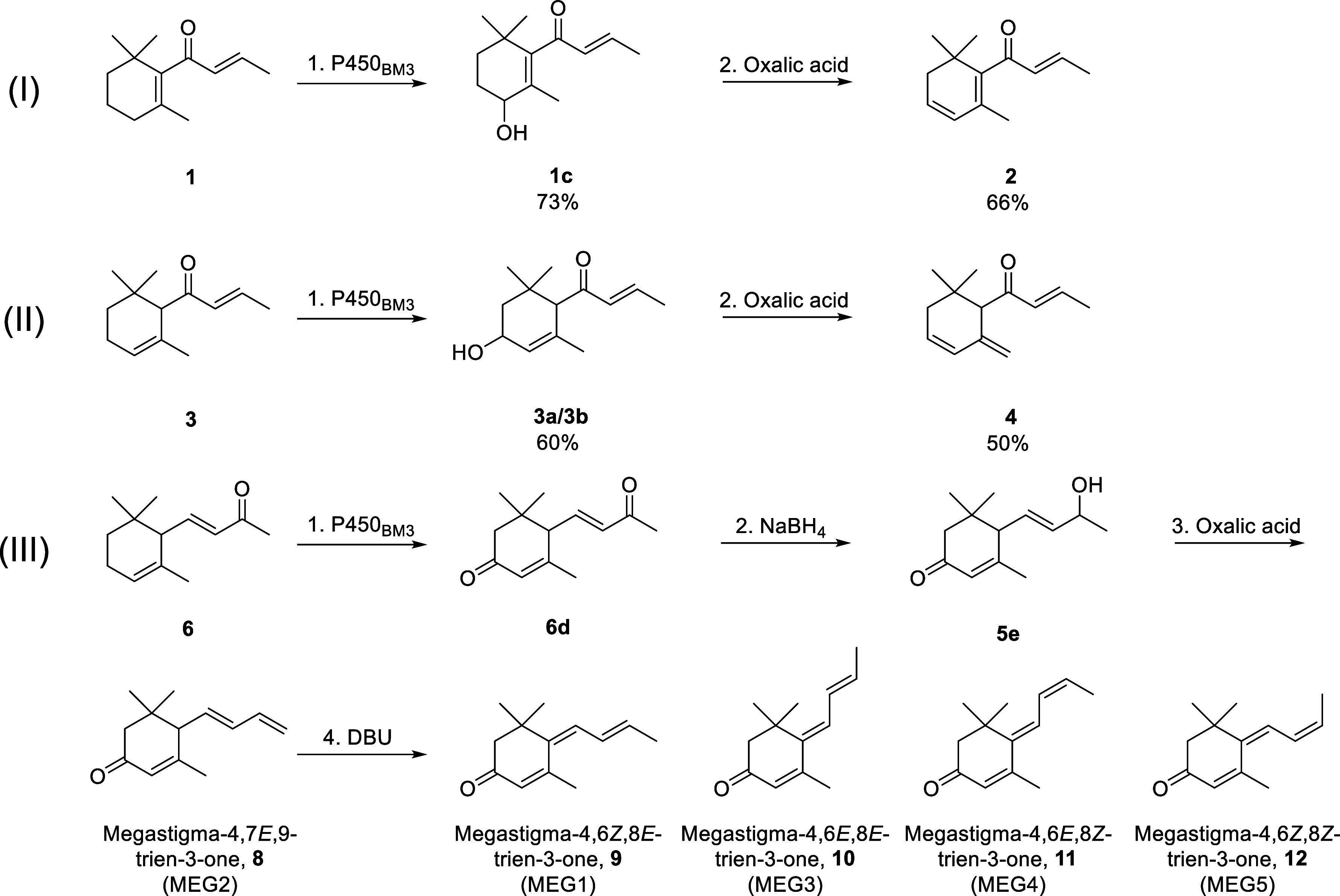
Overview of the synthesis of damascenone compounds **2** and **4** and tabanone isomers **8**–**12**. Reagents and conditions: (I)-1: Variant R19/F87A/A328I/S72F/A330V (0.01 mol %), 16 h, r.t.; (I)-2: oxalic acid, butanol, 65 °C, 16 h. (II)-1: Variant R19/F87A/A328I (0.01 mol %), 16 h, r.t.; (II)-2: oxalic acid, butanol, 65 °C, 16 h. (III)-1: Variant R19/F87A/A328I/E267F (0.02 mol %), 16 h, r.t.; (III)-2: NaBH_4_, methanol, 0.5 h, r.t.; (III)-3: oxalic acid, butanol, 65 °C, 16 h; (III)-4: DBU, dimethoxyethane, 40 °C, 16 h.

## 4 Methods

### 4.1 Enzyme Preparation and Activity Screening

The library of 96 P450_BM3_ enzyme variants was produced in *E. coli* BL21 (DE3) and partially purified by ammonium sulfate fractionation as described previously.^21–24^ For screening scale reactions, the norisoprenoid substrates were dissolved in methanol or ethanol and added as a stock at a 200 mM concentration. Enzymatic activity screening was carried out in a volume of 0.5 mL in 200 mM phosphate buffer (pH 8.0) in 24-well plates. The final concentration of the substrate was 5 mM and the P450_BM3_ variant was at 2 μM. GDH (4 U/mL) and glucose (100 mM) were used to regenerate the NADPH cofactor. NADP^+^ monosodium salt (40 μM) was added last to initiate the reaction. The plates were shaken in the dark in a shaker incubator at 20 °C for 16 h at 120 rpm. The contents of each well were transferred to a 1.5 mL microcentrifuge tube, and organics were extracted by vortexing with 0.3 mL of ethyl acetate. After centrifugation at 14,300 × *g* to separate the phases, the organic layer was removed and analyzed by GC. The oven temperature profiles and retention times of substrates and products are listed in Section S1 in the Supporting Information

### 4.2 Preparative Scale Reactions and Product Purification

Preparative scale reactions (50–1000 mL) for the synthesis of norisoprenoid metabolites with selected enzymes were carried out for 16–24 h under the same conditions as screening scale reactions, except for the larger scale reactions for β-damascone oxidation in which the substrate was added as an 800 mM stock in methanol. Progress of the reactions was monitored by removing a 0.5 mL aliquot at different times, extracting with 0.3 mL of ethyl acetate, and analysis of the organics by GC. Reaction mixtures were then extracted three times with an equal volume of ethyl acetate. The combined extracts were washed with water and brine, dried with Na_2_(SO_4_), and the solvent was removed by rotary evaporation. The crude extracts were purified by silica gel column chromatography. Details on the volume and mass scale of the reactions and column elution solvent mixtures are provided in Section S5 in the Supporting Information.

### 4.3 Synthesis of β-Damascenone

4-Hydroxy-β-damascone (**1c**) (265 mg) was heated at 65 °C for 16 h in butanol (10 mL) with 200 mg of oxalic acid in the presence of activated molecular sieves; **1c** was fully converted to β-damascenone (**2**) by GC analysis. Ethyl acetate (40 mL) was added. The mixture was washed twice with 50 mL of saturated aq. NaHCO_3_ and then 50 mL of brine, dried over Na_2_SO_4_, and filtered, and the solvent was removed by rotary evaporation. The crude extract was purified by silica gel column chromatography, eluting with a 5:1 mixture of petroleum ether (bp 40–60 °C) and ethyl acetate, giving product **2** (160 mg, 66%) as a colorless oil.

### 4.4 Synthesis of γ-Damascenone

A mixture of *cis*-3-hydroxy-α-damascone (**3a**, 4 mg) and *trans*-3-hydroxy-α-damascone (**3b**, 6 mg) was heated at 65 °C for 16 h with 8.6 mg of oxalic acid (2 equiv) in butanol (2 mL). GC analysis showed the full conversion of **3a** and **3b** to γ-damascenone (**4**). Ethyl acetate (50 mL) was added; the mixture was washed twice with 50 mL of saturated aq. NaHCO_3_ and then 50 mL of brine, dried over Na_2_SO_4_, and filtered, and the solvent was removed by rotary evaporation. The crude mixture was purified by silica gel column chromatography, eluting with a 5:1 mixture of petroleum ether (bp 40–60 °C) and ethyl acetate to give γ-damascenone (**4**, 3.1 mg, 34%) as a colorless oil.

### 4.5 Synthesis of Tabanones

3-Oxo-α-ionol (**5e**, 29.6 mg) was heated with oxalic acid (6 equiv) in butanol (4 mL) at 80 °C for 1 h and was fully converted to the terminal dehydration product megastigma-4,7*E*,9-triene-3-one (MEG2, **8**). Ethyl acetate (50 mL) was added to the mixture, which was washed twice with 50 mL of saturated aq. NaHCO_3_ and then 50 mL of brine, dried over Na_2_SO_4_, and filtered, and the solvent was removed by rotary evaporation. The crude mixture was purified by silica gel column chromatography, eluting with a 5:1 mixture of petroleum ether (bp 40–60 °C) and ethyl acetate to give MEG2 (**8**, 15.9 mg, 59%) as a yellowish oil. Treatment of this sample with DBU (3 equiv) at 40 °C in dimethoxyethane (10 mL) for 16 h fully converted **8** to a mixture of **9**–**12**. The reaction mixture was quenched using 10 mL of saturated NH_4_Cl and extracted with dichloromethane followed by washing with 50 mL of brine, dried over Na_2_SO_4_, and filtered, and the solvent was removed by rotary evaporation. The crude mixture was purified by silica gel column chromatography, eluting with dichloromethane to give **9**–**12** (2.2 mg, 14%) as a yellowish oil.

## Supplementary Material



## Notes

Full detail on methods, experimental procedures, list of enzyme variants and the mutations in each, full results of screening scale reactions in tables, chromatograms of gas chromatographic analysis of screening scale reactions, full characterization data, and NMR spectra for all products (PDF)
