# Supplementary material for: Chemoenzymatic Synthesis of Norisoprenoid Aroma Compounds via C–H Activation by Engineered P450BM3
Source: ACS Catal. 2026 Feb 27;16(6):5582–91. doi: 10.1021/acscatal.5c08132 (PMC13010359; doi:10.1021/acscatal.5c08132)
Supplement: Supplementary file 1 [file cs5c08132_si_001.pdf]

Supporting Information

Chemoenzymatic Synthesis of Norisoprenoid Aroma Compounds *via* C–H Activation by Engineered $P450_{BM3}$

Wenyu Chen,[a,b,] Rory Woodhouse,[a] Yuan Zhang,[a,b] Avinash Pandreka,[b]*

Yang Cao,[a,b] Linxue Feng,[b] and Luet L. Wong[a,b,]*

[a] Department of Chemistry, University of Oxford, Inorganic Chemistry Laboratory, South Parks Road, Oxford OX1 3QR, U.K.

[b] Oxford Suzhou Centre for Advanced Research, Ruo Shui Road, Suzhou Industrial Park, Jiangsu, 215123, P.R. China.

S1: Experimental Procedures

S1.1 General

Norisoprenoid substrates were supplied by Merck, Alfa Aesar, Sigma-Aldrich, and Fisher Scientific, UK. Chemical reagents and solvents (HPLC grade) were purchased from Merck, Fisher Scientific, and Acros, UK. Media components and kanamycin were from Melford Laboratories, UK. Isopropyl-β-D-thiogalactopyranoside (IPTG) was from Fisher Scientific, UK. Hen egg white lysozyme was purchased from Sigma-Aldrich. $NADP^+$ monosodium salt was purchased from Prozomix, UK. Glucose dehydrogenase (GDH) was supplied by Codexis, California. Oligonucleotides for site directed mutagenesis were supplied by Eurofins Genetic Service, UK.

Norisoprenoid oxidation products were purified by flash silica gel column chromatography using Geduran Silica 60, 40–63 μm (Fisher Scientific, UK). Analytical thin-layer chromatography (TLC) was performed using petroleum ether (b.p. 40–60 °C)/ethyl acetate mixtures or dichloromethane and bands were visualized under UV or by phosphomolybdic acid staining.

^{1}H, ^{13}C, COSY, HSQC, HMBC, and NOESY NMR spectra were acquired on Bruker AVIII-500 (500/125 MHz), Bruker AVIII-400 (400/100 MHz), or Bruker AV-400 (400/100 MHz) spectrometers. High resolution mass spectra (HRMS) were obtained on a Bruker microTOF mass spectrometer. Gas chromatographic (GC) analyses were carried out with a ThermoFisher Scientific Trace 1300 instrument equipped with a flame ionization detector (FID) and an AI1310 autosampler using a J&W DB-1MS fused silica capillary column (30 m × 0.25 mm; 0.25 μm film thickness, Agilent Technology, UK) with helium as carrier gas at a flow rate of 1.5 mL/min.

S1.2 Enzymes and molecular biology

The gene encoding a $P450_{BM3}$ enzyme variant was cloned in the pET28+ vector by NcoI and BamHI restriction sites.[1] Site-directed mutagenesis was carried out by standard PCR-based protocols using a Q5 High-Fidelity DNA polymerase toolkit from New England Biolabs, UK. The presence of the target mutation was confirmed by DNA sequencing. The relevant plasmid was transformed into chemically competent *E. coli* BL21 (DE3) for enzyme production. P450 enzyme concentrations were determined the difference spectrum method using $\varepsilon_{450-490\ nm}$ = 91000 M^{-1} cm^{-1}.[2] The absolute purity of the enzymes was not determined, and further purification was not necessary for functional characterization or synthetic applications of the enzymes.

S1.3 Activity screening and preparative scale reactions

The norisoprenoid substrates were dissolved in methanol or ethanol and added as a stock at 200 mM concentration. Enzymatic activity screening was carried out in a volume of 0.5 mL in 200 mM phosphate buffer (pH 7.9) in 24-well plates. The final concentration of the substrate was 5 mM and the $P450_{BM3}$ variant was at 2 μM. GDH (4 U/mL) and glucose (100 mM) were used to regenerate the NADPH cofactor. $NADP^+$ monosodium salt (40 μM) was added last to initiate the reaction. Screening plates were shaken at 20 °C for 16 h at 120 rpm. Each reaction was then extracted with 0.3 mL of ethyl acetate. After centrifugation at 14300 ×*g* to separate the phases, the organic extracts were analyzed by GC.

Preparative scale reactions (50–1000 mL) for the synthesis of norisoprenoid metabolites with selected enzymes were carried out for 16–24 h under the same conditions as in the initial screening, except for the larger scale reactions for β-damascone oxidation in which the substrate was added as an 800 mM stock in methanol. Progress of the reactions was monitored

removing a 0.5 mL aliquot at different times, extraction with 0.3 mL ethyl acetate and analysis of the organics by GC. Reaction mixtures were then extracted three times with an equal volume of ethyl acetate. The combined extracts were washed with water and brine, dried with $Na_2(SO_4)$ and the solvent was removed by rotary evaporation. The crude extract was purified by silica gel column chromatography.

For GC analysis of the oxidation of β-damascone (**1**), the oven temperature was held at 140 °C for 1 min then raised at 15 °C/min to 240 °C and held for 1 min. Retention times for compounds were: β-damascone (**1**), 3.81 min; 2-hydroxy-β-damascone (**1a**), 5.14 min; 3-hydroxy-β-damascone (**1b**), 5.08 min; 4-hydroxy-β-damascone (**1c**), 4.97 min; 4-oxo-β-damascone (**1d**), 4.93 min; 10-hydroxy-β-damascone (**1e**), 5.66 min.

For GC analysis of the acid treatment of 4-hydroxy-β-damascone (**1c**), the oven temperature was held at 120 °C for 1 min then raised at 15 °C/min to 240 °C and held for 4 min. Retention times for compounds were: 4-hydroxy-β-damascone (**1c**), 6.21 min; acetate derivative **E1**, 6.89 min; trifluoroacetate derivative **E2**, 5.32 min; β-damascenone (**2**), 4.68 min.

For GC analysis of the oxidation of α-damascone (**3**), the oven temperature was held at 120 °C for 1 min then raised at 15 °C/min to 240 °C and held for 4 min. Retention times for compounds were: α-damascone (**3**), 4.46 min; *cis*-3-hydroxy-α-damascone (**3a**), 5.73 min; *trans*-3-hydroxy-α-damascone (**3b**), 5.89 min; 3-oxo-α-damascone (**3c**), 6.1 min; 3,13-dihydroxy-α-damascone (**3d**), 7.91 min; γ-damascenone (**4**), 4.39 min.

For GC analysis of the oxidation of α-ionol (**5**), the oven temperature was held at 140 °C for 1 min then raised at 15 °C/min to 240 °C and held for 1 min. Retention times for compounds were: α-ionol (**5**), 3.64 min; 3,4-epoxy-α-ionol (**5a**), 4.64 min; *cis*-3-hydroxy-α-ionol (**5b**),

4.98 min; *trans*-3-hydroxy-α-ionol (**5c**), 5.09 min; 13-hydroxy-α-ionol (**5d**), 5.36 min; 3-oxo-α-ionol (**5e**)/3-oxo-α-ionone (**6d**), 5.41 min; 3-oxo-13-hydroxy-α-ionol (**5f**), 5.14 min.

For GC analysis of the oxidation of α-ionone (**6**), the oven temperature was held at 140 °C for 1 min then raised at 15 °C/min to 240 °C and held for 1 min. Retention times for compounds were: α-ionone (**6**), 3.51 min; 4,5-epoxy-α-ionone (**6a**), 4.29 min; *cis*-3-hydroxy-α-ionone (**6b**), 4.94 min; *trans*-3-hydroxy-α-ionone (**6c**), 4.85 min; 3-oxo-α-ionone (**6d**), 5.01 min; 1-(7,7-dimethyl-1,3,5,6,7,7a-hexa-hydroisobenzofuran-1-yl)propan-2-one (7,13-furan, **6e**), 4.51 min.

For GC analysis of the oxidation of megastigmatriene (**7**), the oven temperature was held at 140 °C for 1 min then raised at 15 °C/min to 240 °C and held for 0.5 min. Retention times for compounds were: megastigmatriene (**7**), 3.52 min; 4-hydroxy-β-ionol (**7a**), 5.1 min; 4-oxo-β-ionol (**7b**), 5.5 min; 3,4-dihydroxy-β-ionol (**7c**), 6.25 min.

For GC analysis of megastigmatrienone **8**–**12** synthesis, the oven temperature was held at 140 °C for 1 min then raised at 15 °C/min to 240 °C and held for 0.5 min. Retention times for compounds were: 3-oxo-α-ionol (**5e**), 5.28 min; megastigma-4,7*E*,9-trien-3-one (**8**), 4.04 min; megastigma-4,6*Z*,8*E*-trien-3-one (**9**), 4.84 min; megastigma-4,6*E*,8*E*-trien-3-one (**10**), 5.17 min; megastigma-4,6*E*,8*Z*-trien-3-one (**11**), 5.07 min; megastigma-4,6*Z*,8*Z*-trien-3-one (**12**), 4.71 min.

S2: List of P450$_{BM3}$ variants

Table S1. List of the 96 P450$_{BM3}$ variants in the screening library. GVQ=A74G/F87V/L188Q; GV=A74G/F87V; VQ= F87V/L188Q; K19=H171L/Q307H/N319Y; R19=R47L/Y51F/K19; KU3=N239H/I259V/A276T; RP=R47L/Y51F/I401P.

Entry	Variant	Mutations
1	**M330**	A82M/A330P
2	**M258**	A330P
3	**M217**	GVQ
4	**M216**	GV/A184I
5	**M219**	GVQ/A264G
6	**M8**	GV/A184I/I263G/A328G
7	**M7**	GV/A184I/I263G/A264G/A328G
8	**M331**	I263A/A330P
9	**M326**	K19
10	**M346**	K19/A82M/A264G/A328G
11	**M223**	K19/F87A/F81W
12	**M371**	K19/F87A/A82M
13	**M222**	K19/F87A/A82M/I263G
14	**M25**	K19/F87A/A82M/I263G/A264G
15	**M221**	K19/F87A/A82M/E267F
16	**M224**	K19/F87A/I263A
17	**M379**	K19/F87A/A328I/I263A
18	**M372**	K19/F87A/A328I/A184I
19	**M222**	K19/F87A/A328I/V78I
20	**M304**	K19/F87I
21	**M226**	K19/F87V
22	**M228**	K19/F87V/E267V
23	**M327**	K19/F87V/A328I
24	**M230**	K19/F87V/Q403P
25	**M17**	K19/F87V/A264G
26	**M229**	K19/F87V/E267V/V78I
27	**M231**	KU3/A330P
28	**M262**	KU3/A330P/S72W
29	**M328**	KU3/A330P/V78I
30	**M364**	KU3/A330P/I263G
31	**M232**	KU3/A330P/A328I
32	**M233**	R19
33	**M358**	R19/S72W/A330W
34	**M339**	R19/F81W/T260G/A328G
35	**M345**	R19/A82M/A184I/T260G
36	**M350**	R19/A82M/T260G
37	**M356**	R19/A82M/I263G
38	**M359**	R19/A82M/T260G/A328G/P329G
39	**M361**	R19/A82M/I263G/A328G
40	**M347**	R19/A82M/I263G/A264G/A328G
41	**M357**	R19/A82M/I263W/A328G
42	**M360**	R19/A82M/G265GG
43	**M362**	R19/A82M/P329G/A330W
44	**M235**	R19/F87A
45	**M236**	R19/F87A/A184I
46	**M242**	R19/F87A/I263G
47	**M238**	R19/F87A/A328I
48	**M333**	R19/F87A/A328I/E267F

Entry	Variant	Mutations
49	**M239**	R19/F87A/A328I/S72W
50	**M240**	R19/F87A/A328I/V78I
51	**M239**	R19/F87A/A328I/S72W
52	**M275**	R19/F87A/A328I/S72A
53	**M351**	R19/F87A/A328I/S72H
54	**M471**	R19/F87A/A328I/S72F/A330V
55	**M237**	R19/F87A/A328F
56	**M241**	R19/F87A/A328L
57	**M243**	R19/F87I
58	**M335**	R19/A184I/T260G
59	**M344**	R19/T260G
60	**M341**	R19/I263G
61	**M342**	R19/I263G/A328G
62	**M340**	R19/G265GG/W130C
63	**M337**	R19/A328G/I263W
64	**M336**	R19/A328G/I263G/A264G
65	**M338**	R19/A328G/P329G/A330G
66	**M343**	R19/P329G/A330W
67	**M367**	R47L/Y51F/H171L/I263G/E267F
68	**M373**	RP
69	**M377**	RP/F81W
70	**M252**	RP/V78I/E267V
71	**M253**	RP/A82W/I263A
72	**M245**	RP/A82M/I263A
73	**M365**	RP/A82M/A330W
74	**M246**	RP/F87V
75	**M247**	RP/F87V/V78I
76	**M374**	RP/F87V/E267V
77	**M329**	RP/H171L
78	**M248**	RP/H171L/I263G
79	**M249**	RP/H171L/I263G/A184I
80	**M352**	RP/H171L/I263G/A82M
81	**M355**	RP/H171L/I263G/S72G
82	**M250**	RP/H171L/I263G/F87V/V78I
83	**M348**	RP/H171L/I263G/S72W
84	**M366**	RP/H171L/E267F
85	**M251**	RP/I263A/E267V
86	**M375**	RP/E267V
87	**M254**	RT2
88	**M334**	RT2/H171L/I263G/A330W
89	**M256**	RT2/A330W/S72G
90	**M255**	RT2/A330P/V78I/A184I
91	**M370**	RT2/A330W/S72H
92	**M369**	RT2/A330W/S72Y
93	**M368**	RT2/A330W/I263A
94	**M349**	RT2/A330W/S72G/L437LA
95	**M257**	RT2/A330W/S72W
96	**M269**	VQ/S72G/A330W

S3: Product profiles and selectivity trends

S3.1 Oxidation of β-damascone (1)

Table S2. Screening data of β-damascone (**1**).[a]

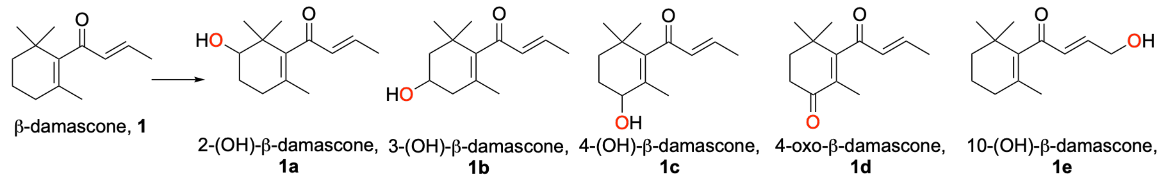

Variant	Mutations [b]	1a	1b	1c	1d	1e	Other	Conv.
**M330**	A82M/A330P			60%	2%	9%	26%	22%
**M8**	GV/A184I/I263G/A328G			91%		9%		55%
**M216**	GV/A184I			73%	3%	7%	14%	87%
**M219**	GVQ/A264G			85%	2%	2%	6%	65%
**M223**	K19/F87A/F81W	3%		74%	8%		12%	85%
**M221**	K19/F87A/A82M/E267F	4%		79%	6%		11%	58%
**M222**	K19/F87A/A82M/I263G			100%				49%
**M379**	K19/F87A/A328I/I263A			93%	4%	3%		89%
**M304**	K19/F87I			73%	5%	19%		93%
**M226**	K19/F87V			74%	2%	10%	11%	87%
**M17**	K19/F87V/A264G			98%	2%			80%
**M228**	K19/F87V/E267V	2%		73%	4%	3%	15%	46%
**M229**	K19/F87V/E267V/V78I	6%		68%	3%	2%	18%	35%
**M327**	K19/F87V/A328I			83%		4%	13%	85%
**M230**	K19/F87V/Q403P			77%	2%	9%	9%	82%
**M231**	KU3/A330P			54%		31%	10%	40%
**M262**	KU3/A330P/S72W			39%		45%	16%	43%
**M364**	KU3/A330P/I263G			95%		3%		78%
**M235**	R19/F87A	3%		82%	4%		11%	52%
**M236**	R19/F87A/A184I			66%	15%		19%	87%
**M242**	R19/F87A/I263G			84%	6%	3%	7%	73%
**M237**	R19/F87A/A328F	21%	5%	51%		14%	9%	79%
**M238**	R19/F87A/A328I	7%	3%	77%			13%	85%
**M471**	R19/F87A/A328I/S72F/A330V			86%	3%		11%	95%
**M333**	R19/F87A/A328I/E267F	11%	32%	41%		6%	10%	97%
**M241**	R19/F87A/A328L	17%	34%	38%			11%	100%
**M243**	R19/F87I		2%	72%	5%	18%	3%	84%
**M252**	RP/V78I/E267V			79%	2%	4%	15%	77%
**M245**	RP/A82M/I263A			93%		7%		52%
**M365**	RP/A82M/A330W			94%		6%		52%
**M246**	RP/F87V			74%	2%	8%	16%	78%
**M247**	RP/F87V/V78I			80%		9%	11%	43%
**M329**	RP/H171L			60%		25%	15%	86%

Variant	Mutations [b]	1a	1b	1c	1d	1e	Other	Conv.
**M248**	RP/H171L/I263G			84%	2%		14%	93%
**M352**	RP/H171L/I263G/A82M			69%		8%	23%	96%
**M355**	RP/H171L/I263G/S72G			98%	2%			84%
**M348**	RP/H171L/I263G/S72W			94%	4%	2%		91%
**M249**	RP/H171L/I263G/A184I			70%	2%	4%	24%	55%
**M366**	RP/H171L/E267F			85%		15%		42%
**M254**	RT2			66%		23%	11%	66%
**M255**	RT2/A330P/V78I/A184I			69%		22%	9%	76%
**M368**	RT2/A330W/I263A			100%				51%
**M256**	RT2/A330W/S72G/			87%		13%		88%
**M349**	RT2/A330W/S72G/L437LA			52%		46%	2%	83%
**M370**	RT2/A330W/S72H			88%	2%	10%		85%
**M369**	RT2/A330W/S72Y			84%		16%		90%
**M257**	RT2/A330W/S72W			50%		35%	15%	45%
**M269**	VQ/S72G/A330W			95%	2%	3%		82%

[a] Only variants with conversions higher than 20% are listed. [b] GVQ=A74G/F87V/L188Q; GV=A74G/F87V; VQ= F87V/L188Q; K19=H171L/Q307H/N319Y; R19=R47L/Y51F/K19; KU3=N239H/I259V/A276T; RP=R47L/Y51F/I401P.

S3.2 Oxidation of α-damascone (3)

Table S3. Screening data of α-damascone, (**3**).[a]

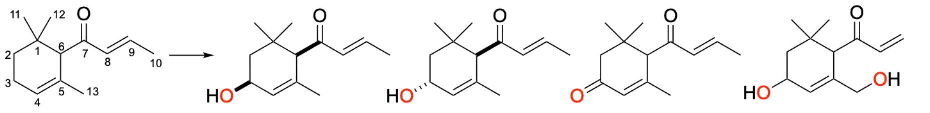

α-Damascone, **3** | *cis*-3-(OH)-α-damascone, **3a** | *trans*-3-(OH)-α-damascone, **3b** | 3-Oxo-α-damascone, **3c** | 3,13-(OH)$_2$-α-damascone, **3d**

Variant	Mutations [b]	3a	3b	3c	3d	Other	Conv.
**M217**	GVQ	19%	39%	11%	16%	5%	48%
**M371**	K19/F87A/A82M	10%	63%	12%	7%	8%	88%
**M378**	K19/F87A/A328I/V78I	9%	75%			16%	30%
**M372**	K19/F87A/A328I/A184I	12%	53%	23%	11%	1%	83%
**M379**	K19/F87A/A328I/I263A	8%	69%	10%		13%	70%
**M304**	K19/F87I	33%	48%	12%		7%	48%
**M230**	K19/F87V/Q403P	13%	56%	6%	6%	19%	43%
**M345**	R19/A82M/A184I/T260G	18%	43%			39%	74%
**M347**	R19/A82M/I263G/A264G/A328G	26%	8%	5%		61%	42%
**M362**	R19/A82M/P329G/A330W	28%	10%	13%		49%	23%
**M235**	R19/F87A	8%	55%	26%	7%	4%	64%
**M238**	R19/F87A/A328I	7%	68%	19%		6%	86%
**M341**	R19/I263G	25%	2%	2%		71%	90%
**M342**	R19/I263G/A328G	14%	2%	3%		81%	77%
**M373**	RP	38%	20%			42%	35%
**M377**	RP/F8IW	41%	21%			38%	45%
**M245**	RP/A82M/I263A	66%	6%			28%	24%
**M246**	RP/F87V	29%	31%	14%	13%	13%	65%
**M374**	RP/F87V/E267V	34%	36%	12%		18%	28%
**M248**	RP/HI7IL/I263G	36%				64%	41%
**M251**	RP/I263A/E267V	52%				48%	37%
**M375**	RP/E267V	30%	34%			36%	23%
**M255**	RT2/A330P/V78I/A184I	15%	28%			57%	31%
**M269**	VQ/S72G/A330W	15%	55%	11%		19%	73%

[a] Only variants with conversions higher than 20% are listed. [b] GVQ=A74G/F87V/L188Q; GV=A74G/F87V; VQ= F87V/L188Q; K19=H171L/Q307H/N319Y; R19=R47L/Y51F/K19; KU3=N239H/I259V/A276T; RP=R47L/Y51F/I401P.

S3.3 Oxidation of α-ionol (5)

Table S4. Screening data for α-ionol, (**5**).[a]

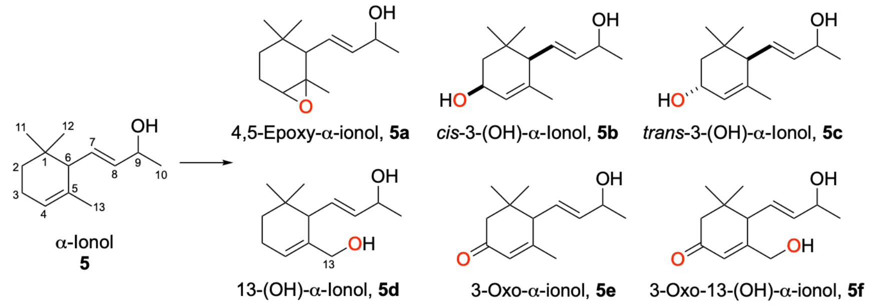

Variant	Mutations [b]	5a	5b	5c	5d	5e	5f	Other	Conv.
**M216**	GV/A184I	11%	2%	6%		2%	20%	59%	100%
**M7**	GV/A184I/I263G/A264G/A328G	47%	14%	3%	6%			30%	75%
**M217**	GVQ		29%	51%	5%	6%		9%	36%
**M223**	K19/F87A/F81W		29%	45%		6%	8%	12%	32%
**M371**	K19/F87A/A82M		6%	14%		57%	15%	8%	100%
**M25**	K19/F87A/A82M/I263G/A264G	7%	45%	33%	7%	5%		3%	29%
**M304**	K19/F87I	17%	10%	57%			6%	10%	44%
**M226**	K19/F87V	15%	4%	26%		14%	16%	25%	100%
**M230**	K19/F87V/Q403P	13%	5%	34%	2%	23%	8%	10%	100%
**M17**	K19/F87V/A264G	51%	9%	16%		10%		19%	88%
**M262**	KU3/A330P/S72W	30%	7%	15%	14%	2%		32%	77%
**M233**	R19	47%	15%	27%	3%				83%
**M345**	R19/A82M/A184I/T260G	32%	4%	36%	2%		4%	22%	100%
**M347**	R19/A82M/I263G/A264G/A328G	6%	7%		70%	2%		15%	76%
**M361**	R19/A82M/I263G/A328G	5%	9%		63%	8%		15%	47%
**M362**	R19/A82M/P329G/A330W	51%	2%	9%	27%				91%
**M350**	R19/A82M/T260G	44%	2%	44%	2%		2%	3%	100%
**M235**	R19/F87A		21%	43%		12%	14%	7%	100%
**M236**	R19/F87A/A184I		17%	35%	3%	4%	34%	5%	100%
**M56**	R19/F87A/T260G/A328G		33%	55%	6%	6%			29%
**M275**	R19/F87A/A328I/S72A	4%	31%	28%	3%	17%	8%	9%	100%
**M239**	R19/F87A/A328I/S72W		45%	24%		16%	4%	11%	47%
**M243**	R19/F87I	13%		62%	3%			22%	40%
**M335**	R19/A184I/T260G	7%	29%	43%			6%	15%	58%
**M336**	R19/A328G/I263G/A264G	18%	9%		45%			28%	94%
**M341**	R19/I263G	28%	16%		3%			53%	100%
**M342**	R19/I263G/A328G	7%	7%		52%	5%		31%	100%
**M244**	R47L/Y51F/I263R	46%	18%		4%			32%	100%
**M252**	RP/V78I/E267V	9%	7%	36%		22%	11%	25%	100%
**M365**	RP/A82M/A330W	30%	14%	6%	22%	3%		25%	63%
**M245**	RP/A82M/I263A	38%	21%	4%	25%			12%	65%
**M248**	RP/H171L/I263G	54%	19%					27%	97%

Variant	Mutations [a]	5a	5b	5c	5d	5e	5f	Other	Conv.
**M352**	RP/H171L/I263G/A82M	30%	27%		26%			17%	100%
**M355**	RP/H171L/I263G/S72G	54%	20%		3%			25%	99%
**M348**	RP/H171L/I263G/S72W	40%	20%		2%			38%	100%
**M254**	RT2	40%	20%	29%	3%		3%	5%	100%
**M256**	RT2/A330W/S72G	44%	17%	23%	4%			12%	77%
**M370**	RT2/A330W/S72H	45%	16%	20%	5%			14%	100%
**M357**	RT2/A330W/S72W	41%	15%	24%	6%			14%	30%
**M369**	RT2/A330W/S72Y	43%	20%	29%				8%	40%

[a] Only variants with conversions higher than 20% are listed. [b] GVQ=A74G/F87V/L188Q; GV=A74G/F87V; VQ= F87V/L188Q; K19=H171L/Q307H/N319Y; R19=R47L/Y51F/K19; KU3=N239H/I259V/A276T; RP=R47L/Y51F/I401P.

S3.4 Oxidation of α-ionone (6)

Table S5. Screening data of α-ionone (**6**).[a]

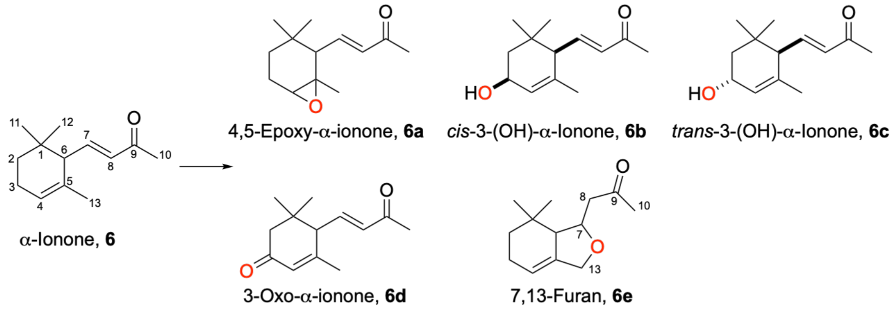

Variant	Mutations [b]	6a	6b	6c	6d	6e	Other	Conv.
**M8**	GV/A184I/I263G/A328G	16%	2%	18%		13%	51%	86%
**M216**	GV/A184I	21%	11%	13%	24%		30%	87%
**M217**	GVQ	19%	42%	11%	10%		18%	59%
**M219**	GVQ/A264G	45%	15%	10%	11%	2%	17%	67%
**M326**	K19	42%	23%	24%			11%	30%
**M223**	K19/F87A/F81W	3%	36%	24%	29%		8%	61%
**M371**	K19/F87A/A82M		44%	23%	24%		9%	95%
**M221**	K19/F87A/A82M/E267F		48%	17%	28%		8%	48%
**M372**	K19/F87A/A328I/A184I		25%	46%	26%		3%	92%
**M379**	K19/F87A/A328I/I263A		56%	29%	8%		7%	85%
**M222**	K19/F87A/A82M/I263G		28%	21%	33%		18%	88%
**M224**	K19/F87A/I263A	4%	62%	24%	6%		4%	34%
**M304**	K19/F87I	5%	15%	40%	33%		7%	81%
**M226**	K19/F87V	29%	16%	7%	21%		27%	84%
**M17**	K19/F87V/A264G	42%	23%	12%	8%		15%	64%
**M327**	K19/F87V/A328I	19%	33%	31%	4%		13%	53%
**M230**	K19/F87V/Q403P	21%	40%	11%	16%		12%	73%
**M231**	KU3/A330P	36%	31%	11%		6%	16%	27%
**M364**	KU3/A330P/I263G	62%		17%		2%	19%	81%
**M233**	R19	52%	4%	16%	5%		23%	88%
**M345**	R19/A82M/A184I/T260G	23%	35%	22%	7%		13%	92%
**M350**	R19/A82M/T260G	32%	50%	13%	4%			60%
**M361**	R19/A82M/I263G/A328G	4%		8%		28%	60%	45%
**M347**	R19/A82M/I263G/A264G/A328G	10%		6%		24%	60%	70%
**M362**	R19/A82M/P329G/A330W	48%	13%	5%		9%	25%	75%
**M335**	R19/A184I/T260G	3%	19%	58%	13%		7%	46%
**M336**	R19/I263G/A264G/A328G	26%	2%	8%		16%	47%	77%
**M235**	R19/F87A		44%	22%	27%		7%	80%
**M236**	R19/F87A/A184I	2%	32%	18%	36%		12%	79%
**M242**	R19/F87A/I263G	18%	38%	15%	12%	2%	15%	75%
**M275**	R19/F87A/A328I/S72A		4%	23%	39%		34%	100%
**M351**	R19/F87A/A328I/S72H		17%	33%	39%		11%	99%
**M239**	R19/F87A/A328I/S72W		29%	31%	30%		10%	94%
**M238**	R19/F87A/A328I		42%	38%	13%		6%	73%

Variant	Mutations [b]	6a	6b	6c	6d	6e	Other	Conv.
**M237**	R19/F87A/A328F		53%	32%	4%		11%	43%
**M333**	R19/F87A/A328I/E267F		69%	15%	8%		8%	92%
**M240**	R19/F87A/A328I/V78I		41%	37%	6%		16%	27%
**M241**	R19/F87A/A328L	14%	70%	14%			2%	91%
**M243**	R19/F87I	5%	12%	42%	33%		8%	88%
**M341**	R19/I263G	42%		28%			30%	97%
**M342**	R19/I263G/A328G	13%		9%		12%	66%	92%
**M343**	R19/P329G/A330W	48%	18%	10%	3%	3%	18%	45%
**M244**	R47L/Y51F/I263R	51%		28%			21%	98%
**M373**	RP	47%	21%	26%			6%	48%
**M252**	RP/V78I/E267V	15%	27%	25%	17%		16%	83%
**M377**	RP/F81W	57%	12%	24%			7%	52%
**M253**	RP/A82M/I263A	24%	14%	26%	2%	13%	21%	67%
**M365**	RP/A82M/A330W	31%	14%	22%	2%	8%	23%	85%
**M246**	RP/F87V	22%	29%	18%	17%		14%	79%
**M329**	RP/H171L	56%	14%	20%	2%		8%	66%
**M248**	RP/H171L/I263G	65%		27%			8%	44%
**M352**	RP/H171L/I263G/A82M	26%	3%	25%		13%	33%	91%
**M355**	RP/H171L/I263G/S72G	58%		20%			22%	91%
**M348**	RP/H171L/I263G/S72W	58%		20%			22%	83%
**M249**	RP/H171L/I263G/A184I	48%		36%			16%	88%
**M254**	RT2	46%	21%	24%	2%		7%	62%
**M334**	RT2/H171L/I263G/A330W	55%	25%				20%	95%
**M255**	RT2/A330P/V78I/A184I	55%	10%	17%		6%	13%	55%
**M368**	RT2/A330W/I263A	76%	2%	9%		3%	10%	50%
**M256**	RT2/A330W/S72G	58%	9%	17%	2%		13%	93%
**M349**	RT2/A330W//S72G/L437LA	22%	26%	43%			9%	31%
**M370**	RT2/A330W/S72H	57%	14%	18%	2%		9%	76%
**M369**	RT2/A330W/S72Y	40%	21%	28%	3%		8%	87%
**M257**	RT2/A330W/S72W	64%	17%	11%			7%	45%
**M269**	VQ/S72G/A330W	19%	12%	7%	11%		51%	92%

[a] Only variants with conversions higher than 20% are listed. [b] GVQ=A74G/F87V/L188Q; GV=A74G/F87V; VQ= F87V/L188Q; K19=H171L/Q307H/N319Y; R19=R47L/Y51F/K19; KU3=N239H/I259V/A276T; RP=R47L/Y51F/I401P.

S4: GC analysis data

S4.1 Oxidation of β-damascone (1)

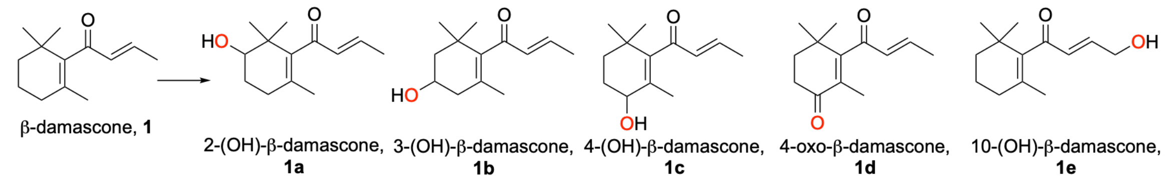

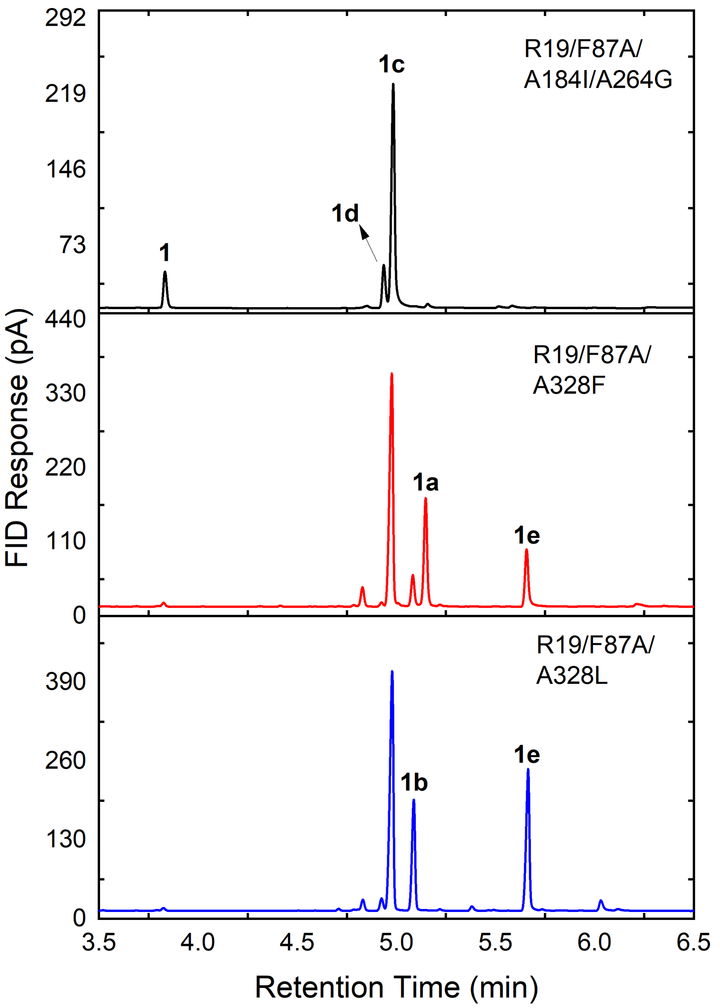

Figure S1. GC analysis of selected variants showing β-damascone oxidation products **1a**–**1e**.

S4.2 Oxidation of α-damascone (3)

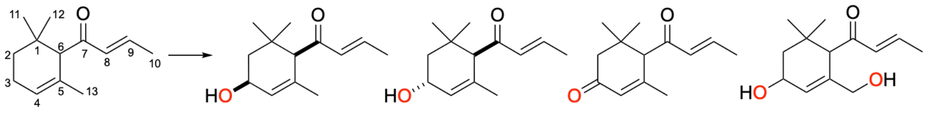

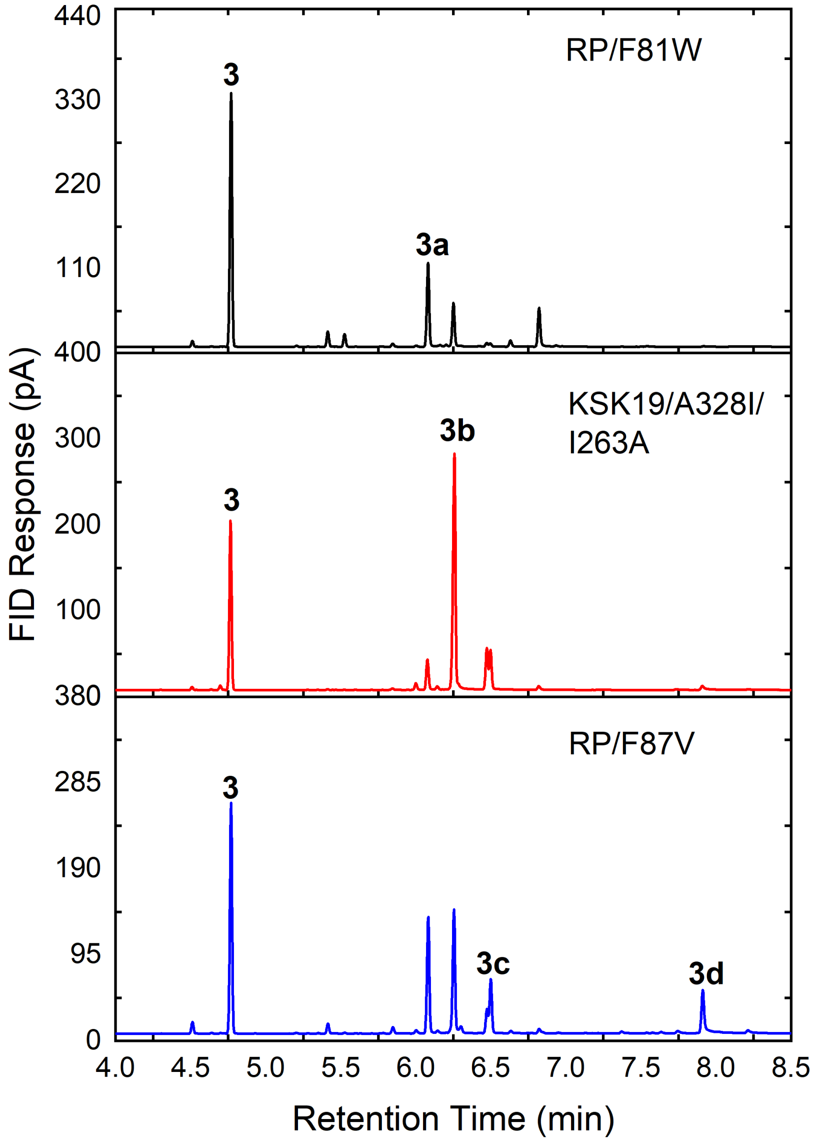

Figure S2. GC analysis of selected variants showing α-damascone oxidation products **3a**–**3d**.

S4.3 Oxidation of α-ionol, (5)

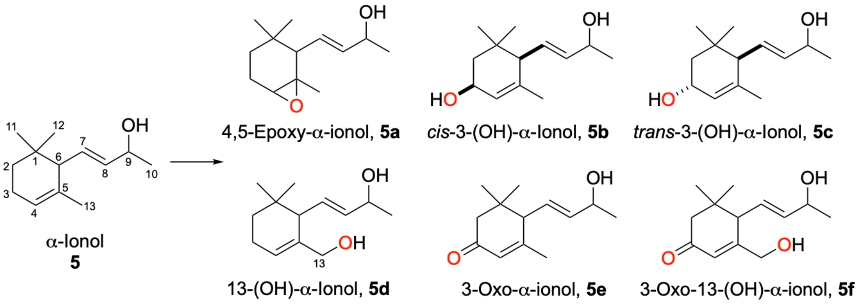

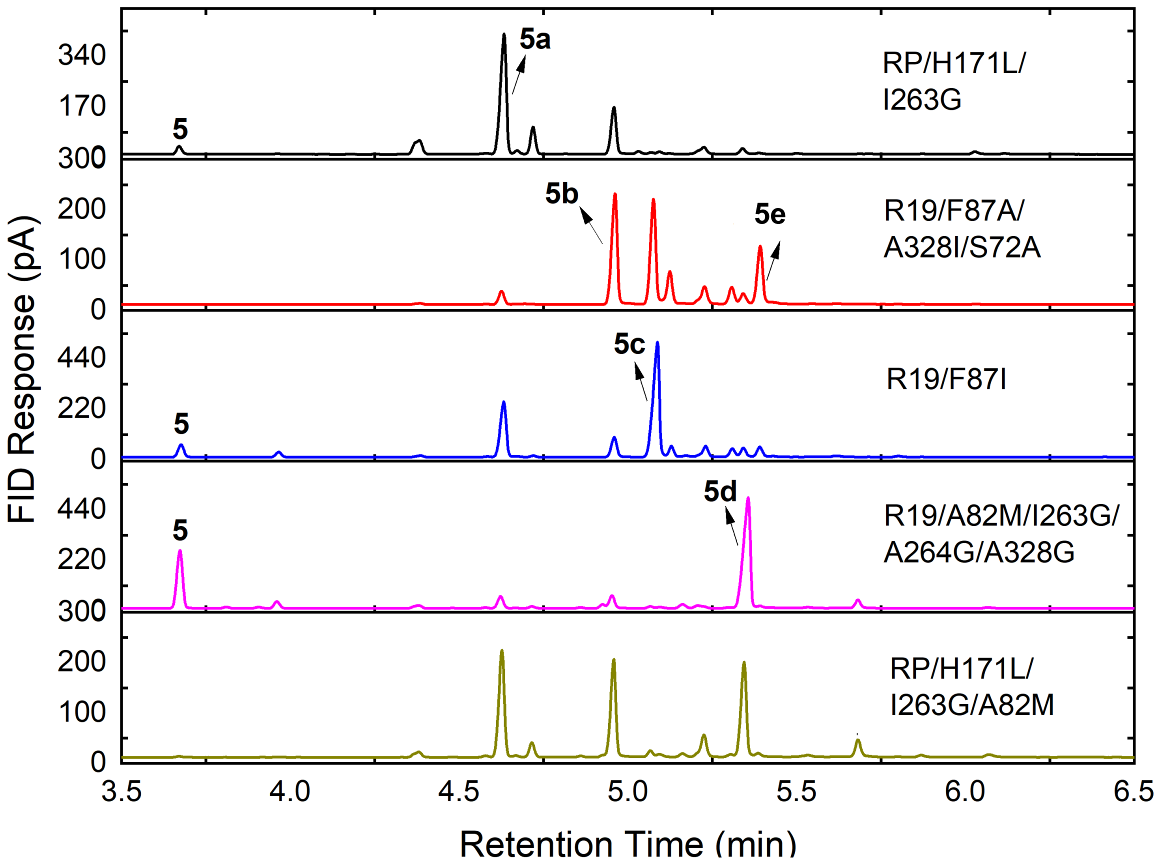

Figure S3. GC analysis of selected variants showing α-ionol oxidation products **5a**–**5e**.

S4.4 Oxidation of α-ionone, (6)

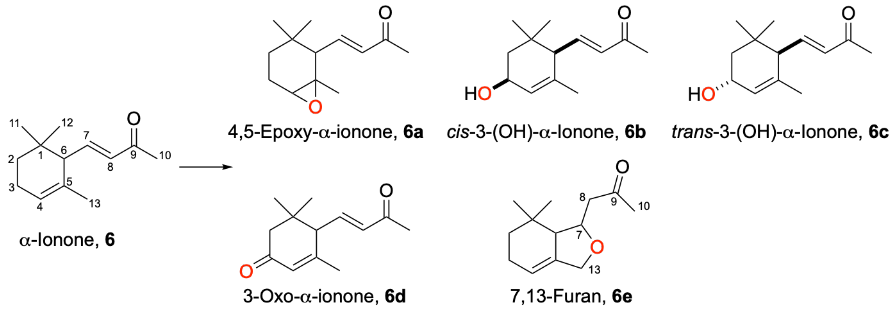

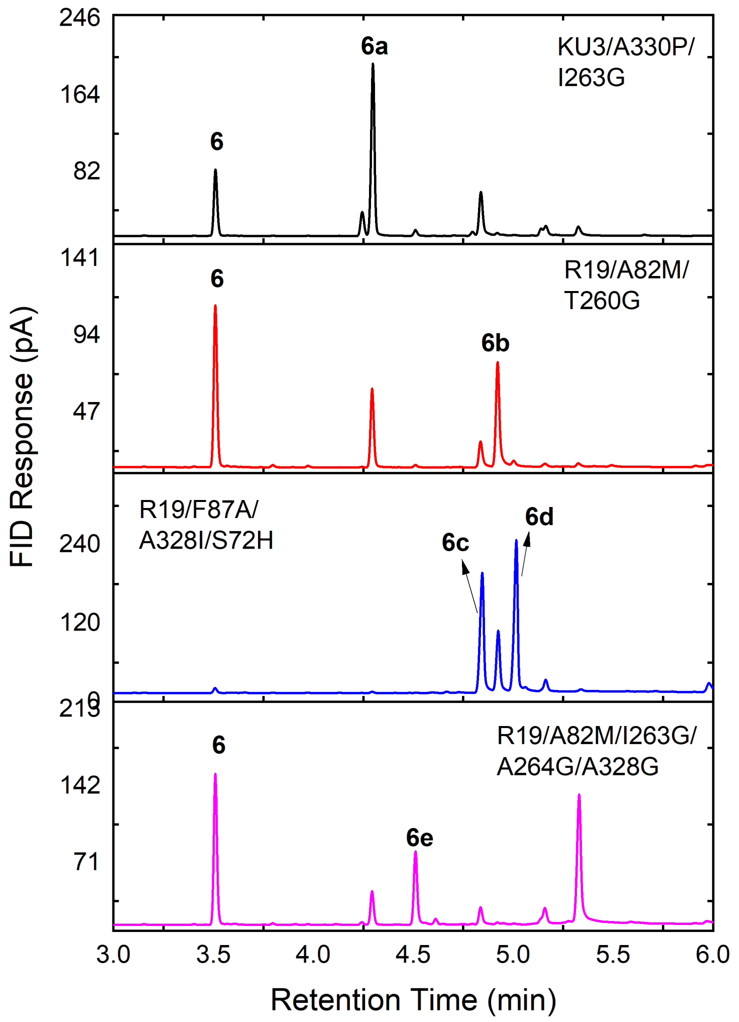

Figure S4. GC analysis of selected variants showing α-ionone oxidation products **6a**–**6e**.

S4.5 Oxidation of (*E*/*E*)-megastigmatriene, (7)

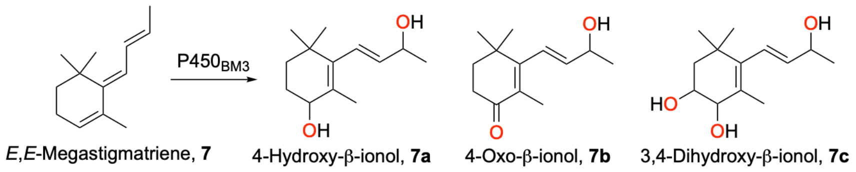

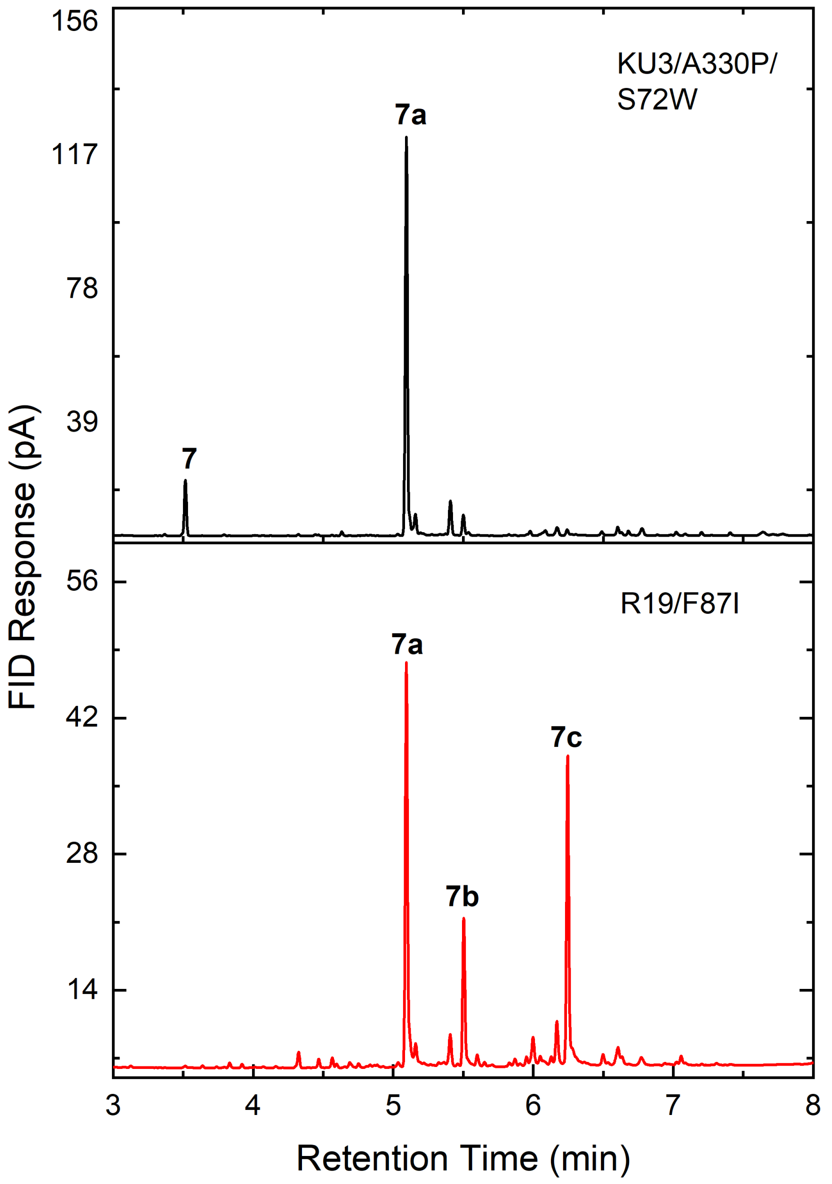

Figure S5. GC analysis of selected variants showing megastigmatriene oxidation products **7a**–**7c**.

S5: Product information

1a: 2-Hydroxy-β-damascone

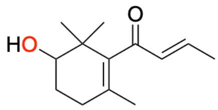

The 100 mL reaction mixture (200 mM potassium phosphate buffer, pH 7.9) in a 500 mL conical flask contained the $P450_{BM3}$ variant R19/F87A/A328F (2 μM), β-damascone (**1**) (96 mg, 5 mM added as a 200 mM solution stock in methanol), glucose dehydrogenase (4 U/mL, 4 U/μL stock) and glucose (100 mM, 10 mL of a 1 M stock). The reaction was initiated by the addition of $NADP^+$ monosodium salt (40 μM, 1 mL of a 4.0 mM solution). After the flask was shaken at 120 rpm, 20 °C for 16 h, GC analysis showed 79% conversion of **1** with 21% selectivity for **1a**. The reaction mixture was extracted three times with 50 mL ethyl acetate; the combined extracts were washed with water and then brine, dried over Na_2SO_4 and the solvent was removed by rotary evaporation. The crude mixture was purified by silica gel column chromatography. Product **1a** was eluted with 5:1 mixture of petroleum ether (bp 40–60 °C) and ethyl acetate to give a pale yellow mixture with compound **1e**.

^{1}H NMR (600 MHz, $CDCl_3$) δ 6.76 (dd, J = 16.0, 7.0 Hz, 1H), 6.15 (dq, J = 16.0, 1.5 Hz, 1H), 3.56 (dd, J = 8.5, 3.0 Hz, 1H), 2.21 – 2.13 (m, 1H), 2.09 (m, 1H), 1.92 (dd, J = 7.0, 1.5 Hz, 3H), 1.91 – 1.87 (m, 1H), 1.78 (ddt, J = 13.5, 8.5, 7.0 Hz, 1H), 1.52 (br s, 3H), 1.06 (s, 3H), 1.04 (s, 3H). **^{13}C NMR** (151 MHz, $CDCl_3$) δ 201.3, 146.6, 146.5, 138.6, 134.7, 130.1, 75.0, 38.2, 28.7, 27.0, 26.2, 22.3, 21.1, 18.6. **HRMS** (ESI) $[M+H]^+$ Calcd for $C_{13}H_{21}O_2^+$: 209.1536, Found: 209.1532.

1b: 3-Hydroxy-β-damascone

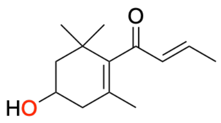

The 100 mL reaction mixture (200 mM potassium phosphate buffer, pH 7.9) in a 500 mL conical flask contained the $P450_{BM3}$ variant R19/F87A/A328L (2 μM), β-damascone (**1**) (96 mg, 5 mM added as a 200 mM solution stock in methanol), glucose dehydrogenase (GDH, 4 U/mL, 4 U/μL stock) and glucose (100 mM, 10 mL of a 1 M stock). The reaction was initiated by the addition of $NADP^+$ monosodium salt (40 μM, 1 mL of a 4.0 mM solution). After the flask was shaken at 120 rpm, 20 °C for 16 h, GC analysis showed 97% conversion of **1** with 32% selectivity for **1b**. The reaction mixture was extracted three times with 50 mL ethyl acetate; the combined extracts were washed with water and then brine, dried over Na_2SO_4 and the solvent was removed by rotary evaporation. The crude mixture was purified by silica gel column chromatography. Product **1b** was eluted with 5:1 mixture of petroleum ether (bp 40–60 °C) and ethyl acetate to give a yellowish oil. The assignment of **1b** as 3-hydroxy-β-damascone was consistent with literature data.[3]

^{1}H NMR (500 MHz, Chloroform-*d*) δ 6.72 (dq, *J* = 16.0, 7.0 Hz , 1H), 6.15 (dq, *J* = 16.0, 1.5 Hz, 1H), 4.08 (dddd, *J* = 11.0, 9.5, 6.0, 4.0 Hz, 1H), 2.36 (ddd, *J* = 17.0, 6.0, 1.5 Hz, 1H), 2.02 (ddd, *J* = 17.0, 9.5, 1.5 Hz, 1H), 1.93 (dd, *J* = 7.0, 1.5 Hz, 3H), 1.74 (ddd, *J* = 12.0, 4.0, 1.5 Hz, 1H), 1.55 (s, 3H), 1.51 (t, *J* = 12.0 Hz, 1H), 1.15 (s, 3H), 0.99 (s, 3H). **^{13}C NMR** (126 MHz, $CDCl_3$) δ 201.8, 146.3, 140.1, 134.6, 128.2, 65.0, 48.0, 41.0, 36.5, 29.8, 29.2, 21.2, 18.6. **HRMS** (ESI) $[M+H]^+$ Calcd for $C_{13}H_{20}NaO_2^+$: 231.1356, Found: 231.1350.

1c: 4-Hydroxy-β-damascone

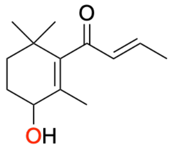

The 100 mL reaction mixture (200 mM potassium phosphate buffer, pH 7.9) in a 500 mL conical flask contained the $P450_{BM3}$ variant RP/H171L/I263G/S72G (2 µM), β-damascone (**1**) (96 mg, 5 mM added as a 200 mM solution stock in methanol), glucose dehydrogenase (GDH, 4 U/mL, 4 U/µL stock) and glucose (100 mM, 10 mL of a 1 M stock). The reaction was initiated by the addition of $NADP^+$ monosodium salt (40 µM, 1 mL of a 4.0 mM solution). After the flask was shaken at 120 rpm, 20 °C for 16 h, GC analysis showed 84% conversion of **1** with 98% selectivity for **1c**. The reaction mixture was extracted three times with 50 mL ethyl acetate; the combined extracts were washed with water and then brine, dried over Na_2SO_4 and the solvent was removed by rotary evaporation. The crude mixture was purified by silica gel column chromatography. Product **1c** was eluted with 5:1 mixture of petroleum ether (bp 40–60 °C) and ethyl acetate to give a yellowish oil. The assignment of **1c** as 4-hydroxy-β-damascone was consistent with literature data.[3]

^{1}H NMR (400 MHz, $CDCl_3$) δ 6.76 (dq, J = 16.0, 7.0 Hz, 1H), 6.13 (dq, J = 16.0, 1.5 Hz, 1H), 3.97 (t, J = 5.0 Hz, 1H), 1.97 (m, 1H), 1.91 (dd, J = 7.0, 1.5 Hz, 3H), 1.75 (m, 1H), 1.68 – 1.62 (m, 1H), 1.62 (s, 3H), 1.47 – 1.37 (m, 1H), 1.01 (s, 3H), 1.01 (s, 3H). **^{13}C NMR** (101 MHz, $CDCl_3$) δ 201.6, 147.2, 143.1, 133.8, 131.4, 68.7, 34.7, 33.9, 28.8, 28.5, 27.7, 18.5, 18.0. **HRMS** (ESI) $[M+H]^+$ Calcd for $C_{13}H_{20}NaO_2^+$: 231.1356, Found: 231.1351.

1d: 4-Oxo-β-damascone

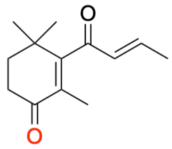

The 100 mL reaction mixture (200 mM potassium phosphate buffer, pH 7.9) in a 500 mL conical flask contained the P450$_{BM3}$ variant R19/F87A/A184I (2 μM), β-damascone (**1**) (96 mg, 5 mM added as a 200 mM solution stock in methanol), glucose dehydrogenase (GDH, 4 U/mL, 4 U/μL stock) and glucose (100 mM, 10 mL of a 1 M stock). The reaction was initiated by the addition of $NADP^+$ monosodium salt (40 μM, 1 mL of a 4.0 mM solution). After the flask was shaken at 120 rpm, 20 °C for 16 h, GC analysis showed 87% conversion of **1** with 15% selectivity for **1d**. The reaction mixture was extracted three times with 50 mL ethyl acetate; the combined extracts were washed with water and then brine, dried over Na_2SO_4 and the solvent was removed by rotary evaporation. The crude mixture was purified by silica gel column chromatography. Product **1d** was eluted with 5:1 mixture of petroleum ether (bp 40–60 °C) and ethyl acetate to give a yellowish oil. The assignment of **1d** as 4-oxo-β-damascone was consistent with literature data.[4]

^{1}H NMR (400 MHz, $CDCl_3$) δ 6.74 (dq, *J* = 16.0, 7.0 Hz, 1H), 6.19 (dq, *J* = 16.0, 1.5 Hz, 1H), 2.57 (dd, *J* = 7.0, 6.5 Hz, 2H), 1.97 (dd, *J* = 7.0, 1.5 Hz, 3H), 1.93 (m, 2H), 1.62 (s, 3H), 1.19 (s, 6H). ^{13}C NMR (151 MHz, $CDCl_3$) δ 198.9, 197.9, 160.9, 148.0, 132.9, 129.5, 38.0, 34.7, 34.3, 27.3, 18.7, 13.1. **HRMS** (ESI) $[M+H]^+$ Calcd for $C_{13}H_{19}O_2^+$: 207.1380, Found: 207.1377.

1e: 10-Hydroxy-β-damascone

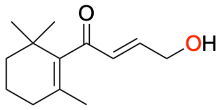

The 100 mL reaction mixture (200 mM potassium phosphate buffer, pH 7.9) in a 500 mL conical flask contained the $P450_{BM3}$ variant RT2/S72G/A330W/L437LA (2 μM), β-damascone (**1**) (96 mg, 5 mM added as a 200 mM solution stock in methanol), glucose dehydrogenase (GDH, 4 U/mL, 4 U/μL stock) and glucose (100 mM, 10 mL of a 1 M stock). The reaction was initiated by the addition of $NADP^+$ monosodium salt (40 μM, 1 mL of a 4.0 mM solution). After the flask was shaken at 120 rpm, 20 °C for 16 h, GC analysis showed 83% conversion of **1** with 46% selectivity for **1e**. The reaction mixture was extracted three times with 50 mL ethyl acetate; the combined extracts were washed with water and then brine, dried over Na_2SO_4 and the solvent was removed by rotary evaporation. The crude mixture was purified by silica gel column chromatography. Product **1e** was eluted with 5:1 mixture of petroleum ether (bp 40–60 °C) and ethyl acetate to give a yellowish oil.

^{1}H NMR (400 MHz, $CDCl_3$) δ 6.78 (dt, J = 16.0, 4.0 Hz, 1H), 6.37 (dt, J = 16.0, 2.0 Hz, 1H), 4.38 (dd, J = 4.0, 2.0 Hz, 2H), 1.98 (td, J = 6.5, 1.0 Hz, 2H), 1.71 – 1.65 (m, 2H), 1.51 (s, 3H), 1.48 – 1.43 (m, 2H), 1.02 (s, 6H). **^{13}C NMR** (101 MHz, $CDCl_3$) δ 202.1, 147.7, 140.4, 131.1, 131.0, 62.2, 38.9, 33.6, 31.3, 29.0, 21.5, 19.0. **HRMS** (ESI) $[M+H]^+$ Calcd for $C_{13}H_{21}O_2^+$: 209.1536, Found: 209.1534.

2: β-Damascenone

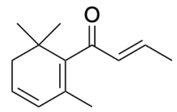

4-Hydroxy-β-damascone (**1c**) (265 mg) was heated at 65 °C for 16 h in butanol (10 mL) with 200 mg of oxalic acid in the presence of activated molecular sieves; **1c** was fully converted to β-damascenone (**2**) by GC analysis. Ethyl acetate (40 mL) was added. The mixture was washed twice with 50 mL of saturated aq. $NaHCO_3$ and then 50 mL of brine, dried over Na_2SO_4, filtered, and the solvent was removed by rotary evaporation. The crude extract was purified by silica gel column chromatography, eluting with a 5:1 mixture of petroleum ether (bp 40–60 °C) and ethyl acetate giving product **2** (160 mg, 66%) as a colorless oil. The assignment of **2** as β-damascenone was consistent with literature data.[5]

^{1}H NMR (400 MHz, Chloroform-*d*) δ 6.81 (dq, J = 16.0, 7.0 Hz, 1H), 6.15 (dq, J = 16.0, 1.5 Hz, 1H), 5.85 – 5.73 (m, 2H), 2.08 (dd, J = 4.0, 1.5 Hz, 2H), 1.90 (dd, J = 7.0, 1.5 Hz, 3H), 1.60 (s, 3H), 1.01 (s, 6H). **^{13}C NMR** (101 MHz, $CDCl_3$) δ 201.2, 146.4, 139.4, 134.7, 128.2, 128.1, 127.4, 39.5, 33.9, 26.4, 19.6, 18.5. **HRMS** (ESI) $[M+H]^+$ Calcd for $C_{13}H_{19}O^+$: 191.1430, Found: 191.1432.

E1: (*E*)-3-(But-2-enoyl)-2,4,4-trimethylcyclohex-2-en-1-yl-acetate

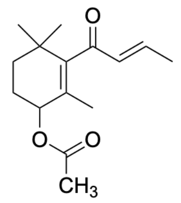

4-Hydroxy-β-damascone (**1c**) was treated with acetic acid and acetic anhydride at 85 °C with stirring for 6 h; full conversion to the acetate derivative **E1** was observed by TLC and GC. After cooling to room temperature, 50 mL of ethyl acetate was added and the mixture washed twice with 50 mL saturated aq. $NaHCO_3$ and then 50 mL brine, dried over Na_2SO_4, filtered, and the solvent was removed by rotary evaporation. The mixture was purified by silica gel column chromatography, eluting with a 5:1 mixture of petroleum ether (bp 40–60 °C) and ethyl acetate to give ester **E1** as a colorless oil.

^{1}H NMR (400 MHz, $CDCl_3$) δ 6.77 (dq, *J* = 16.0, 7.0 Hz, 1H), 6.16 (dq, *J* = 16.0, 1.5 Hz, 1H), 5.21 (t, *J* = 5.0 Hz, 1H), 2.08 (s, 3H), 2.05 – 1.95 (m, 1H), 1.95 – 1.90 (dd, *J* = 7.0, 1.5 Hz, 3H), 1.78 (dtt, *J* = 11.0, 4.5, 2.5 Hz, 1H), 1.68 – 1.59 (m, 1H), 1.50 (br s, 3H), 1.45 (ddd, *J* = 13.0, 7.5, 3.5 Hz, 1H), 1.05 (s, 3H), 1.04 (s, 3H). **^{13}C NMR** (101 MHz, $CDCl_3$) δ 200.5, 171.1, 146.8, 145.9, 134.0, 127.7, 71.1, 34.9, 34.0, 28.8, 25.5, 21.4, 18.7, 17.9.

E2: (*E*)-3-(But-2-enoyl)-2,4,4-trimethylcyclohex-2-en-1-yl 2,2,2-trifluoroacetate

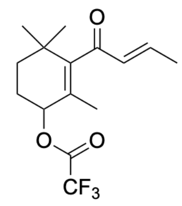

4-Hydroxy-β-damascone (**1c**) was stirred with trifluoroacetic acid at room temperature for 36 h; full conversion to the trifluoroacetate ester **E2** was observed using TLC and GC. Ethyl acetate (50 mL) was added, and the mixture was washed twice with 50 mL saturated aq. $NaHCO_3$ and then 50 mL brine, dried over Na_2SO_4, filtered, and the solvent was removed by rotary evaporation. The mixture was purified by silica gel column chromatography, eluting with a 5:1 mixture of petroleum ether (bp 40–60 °C) and ethyl acetate to give product **E2** as a colorless oil.

^{1}H NMR (400 MHz, $CDCl_3$) δ 6.76 (dq, *J* = 16.0, 7.0 Hz, 1H), 6.17 (dq, *J* = 16.0, 1.5 Hz, 1H), 5.38 (t, *J* = 5.0 Hz, 1H), 2.10 (dddd, *J* = 15.0, 11.5, 5.0, 3.5 Hz, 1H), 1.96 (dd, *J* = 7.0, 1.5 Hz, 3H), 1.94 – 1.86 (m, 1H), 1.68 (ddd, *J* = 14.5, 11.5, 3.5 Hz, 1H), 1.54 (br s, 3H), 1.53 – 1.46 (m, 1H), 1.08 (s, 3H), 1.06 (s, 3H). **^{13}C NMR** (101 MHz, $CDCl_3$) δ 199.6, 148.1, 147.3, 133.7, 125.3, 76.3, 34.3, 34.1, 28.7, 27.3, 25.1, 18.7, 17.9.

3a: *cis*-3-Hydroxy-α-damascone

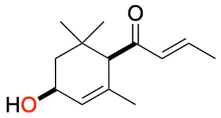

The 100 mL reaction mixture (200 mM potassium phosphate buffer, pH 7.9) in a 500 mL conical flask contained the P450BM3 variant RLYF/K19/F87A/A328I (2 μM), α-damascone (**3**) (96 mg, 5 mM added as a 200 mM solution stock in methanol), glucose dehydrogenase (GDH, 4 U/mL, 4 U/μL stock) and glucose (100 mM, 10 mL of a 1 M stock). The reaction was initiated by the addition of $NADP^+$ monosodium salt (40 μM, 1 mL of a 4.0 mM solution). After the flask was shaken at 120 rpm, 20 °C for 16 h, GC analysis showed 86% conversion of **3** with 3% selectivity for **3a**. The reaction mixture was extracted three times with 50 mL ethyl acetate; the combined extracts were washed with water and then brine, dried over Na_2SO_4 and the solvent was removed by rotary evaporation. The crude mixture was purified by silica gel column chromatography, eluting with a 5:1 mixture of petroleum ether (bp 40–60 °C) and ethyl acetate to give product **3a** as a yellowish oil.

^{1}H NMR (500 MHz, $CDCl_3$) δ 6.91 (dq, *J* = 15.5, 7.0 Hz, 1H), 6.32 (dq, *J* = 15.5, 1.5 Hz, 1H), 5.68 (dq, *J* = 2.5, 1.0 Hz, 1H), 4.19 (th, *J* = 8.0, 2.0 Hz, 1H), 2.93 (d, *J* = 1.5 Hz, 1H), 1.91 (dd, *J* = 7.0, 1.5 Hz, 3H), 1.64 (d, *J* = 8.0 Hz, 2H), 1.59 (br s, 3H), 0.97 (s, 3H), 0.87 (s, 3H). **^{13}C NMR** (126 MHz, $CDCl_3$) δ 201.5, 143.5, 133.5, 132.6, 127.8, 66.3, 60.9, 41.0, 35.1, 28.8, 28.4, 23.2, 18.5. **HRMS** (ESI) $[M+Na]^+$ Calcd for $C_{13}H_{20}NaO_2^+$: 231.1356, Found: 231.1355.

3b: *trans*-3-Hydroxy-α-damascone

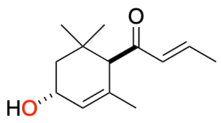

The 100 mL reaction mixture (200 mM potassium phosphate buffer, pH 7.9) in a 500 mL conical flask contained the $P450_{BM3}$ variant RLYF/K19/F87A/A328I (2 μM), α-damascone (**3**) (96 mg, 5 mM added as a 200 mM solution stock in methanol), glucose dehydrogenase (GDH, 4 U/mL, 4 U/μL stock) and glucose (100 mM, 10 mL of a 1 M stock). The reaction was initiated by the addition of $NADP^+$ monosodium salt (40 μM, 1 mL of a 4.0 mM solution). After the flask was shaken at 120 rpm, 20 °C for 16 h, GC analysis showed 86% conversion of **3** with 68% selectivity for **3b**. The reaction mixture was extracted three times with 50 mL ethyl acetate; the combined extracts were washed with water and then brine, dried over Na_2SO_4 and the solvent was removed by rotary evaporation. The crude mixture was purified by silica gel column chromatography, eluting with a 5:1 mixture of petroleum ether (bp 40–60 °C) and ethyl acetate to give **3b** as a yellowish oil. The assignment of **3b** as *trans*-3-hydroxy-α-damascone was consistent with literature data.[6]

^{1}H NMR (400 MHz, $CDCl_3$) δ 6.89 (dq, J = 15.5, 7.0 Hz, 1H), 6.22 (dq, J = 15.5, 1.5 Hz, 1H), 5.70 (dt, J = 3.0, 1.5 Hz, 1H), 4.37 – 4.30 (m, 1H), 3.12 (s, 1H), 1.98 – 1.92 (m, 1H), 1.90 (dd, J = 7.0, 1.5 Hz, 3H), 1.62 (br s, 3H), 1.39 (dd, J = 13.5, 5.5 Hz, 1H), 1.11 (s, 3H), 0.87 (s, 3H). **^{13}C NMR** (101 MHz, $CDCl_3$) δ 200.7, 143.1, 134.3, 132.7, 126.8, 65.6, 61.1, 43.7, 33.5, 30.7, 26.0, 22.9, 18.4. **HRMS** (ESI) $[M+Na]^+$ Calcd for $C_{13}H_{20}NaO_2^+$: 231.1356, Found: 231.1355.

3c: 3-Oxo-α-damascone

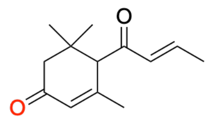

The 100 mL reaction mixture (200 mM potassium phosphate buffer, pH 7.9) in a 500 mL conical flask contained the $P450_{BM3}$ variant RLYF/K19/F87A/A328I (2 μM), α-damascone (**3**) (96 mg, 5 mM added as a 200 mM solution stock in methanol), glucose dehydrogenase (GDH, 4 U/mL, 4 U/μL stock) and glucose (100 mM, 10 mL of a 1 M stock). The reaction was initiated by the addition of $NADP^+$ monosodium salt (40 μM, 1 mL of a 4.0 mM solution). After the flask was shaken at 120 rpm, 20 °C for 16 h, GC analysis showed 86% conversion of **3** with 19% selectivity for **3c**. The reaction mixture was extracted three times with 50 mL ethyl acetate; the combined extracts were washed with water and then brine, dried over Na_2SO_4 and the solvent was removed by rotary evaporation. The crude mixture was purified by silica gel column chromatography, eluting with a 5:1 mixture of petroleum ether (bp 40–60 °C) and ethyl acetate to give **3c** as a yellowish oil. The assignment of **3c** as 3-oxo-α-damascone was consistent with literature data.[7]

^{1}H NMR (400 MHz, $CDCl_3$) δ 6.97 (dq, J = 15.4, 7.0 Hz, 1H), 6.28 (dd, J = 15.4, 2.0 Hz, 1H), 5.99 (s, 1H), 3.37 (s, 1H), 2.66 (d, J = 16.5 Hz, 1H), 2.01 (d, J = 16.5 Hz, 1H), 1.94 (dd, J = 7.0, 2.0 Hz, 3H), 1.84 (d, J = 1.3 Hz, 3H), 1.07 (s, 3H), 0.98 (s, 3H). **^{13}C NMR** (101 MHz, $CDCl_3$) δ 199.3, 197.9, 156.2, 145.0, 132.5, 127.7, 61.8, 47.1, 36.8, 29.3, 27.7, 24.1, 18.6. **HRMS** (ESI) $[M+H]^+$ Calcd for $C_{13}H_{19}O_2^+$: 206.1307, Found: 206.1377.

3d: 3,13-Dihydroxy-α-damascone

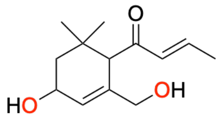

The 100 mL reaction mixture (200 mM potassium phosphate buffer, pH 7.9) in a 500 mL conical flask contained the $P450_{BM3}$ variant GVQ (2 μM), α-damascone (**3**) (96 mg, 5 mM added as a 200 mM solution stock in methanol), glucose dehydrogenase (GDH, 4 U/mL, 4 U/μL stock) and glucose (100 mM, 10 mL of a 1 M stock). The reaction was initiated by the addition of $NADP^+$ monosodium salt (40 μM, 1 mL of a 4.0 mM solution). After the flask was shaken at 120 rpm, 20 °C for 16 h, GC analysis showed 48% conversion of **3** with 16% selectivity for **3d**. The reaction mixture was extracted three times with 50 mL ethyl acetate; the combined extracts were washed with water and then brine, dried over Na_2SO_4 and the solvent was removed by rotary evaporation. The crude mixture was purified by silica gel column chromatography, eluting with a 5:1 mixture of petroleum ether (bp 40–60 °C) and ethyl acetate to give **3d** as a yellowish oil.

^{1}H NMR (400 MHz, MeOD) δ 6.92 (dq, *J* = 15.5, 7.0 Hz, 1H), 6.27 (dq, *J* = 15.5, 1.5 Hz, 1H), 5.83 (dq, *J* = 3.0, 1.5 Hz, 1H), 4.26 (tdt, *J* = 6.0, 3.0, 1.5 Hz, 1H), 3.91 – 3.77 (m, 2H), 3.46 (d, *J* = 1.5 Hz, 1H), 1.93 – 1.82 (m, 4H), 1.40 (dd, *J* = 13.5, 5.5 Hz, 1H), 1.10 (s, 3H), 0.82 (s, 3H). **^{13}C NMR** (101 MHz, MeOD) δ 202.8, 144.8, 139.1, 134.5, 127.6, 65.6, 65.5, 57.2, 44.7, 34.3, 30.8, 26.1, 18.4.

4: γ-Damascenone

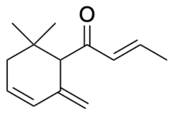

A mixture of *cis*-3-hydroxy-α-damascone (**3a**, 4 mg) and *trans*-3-hydroxy-α-damascone (**3b,** 6 mg) was heated at 65 °C for 16 h with 8.6 mg of oxalic acid (2 equiv.) in butanol (2 mL). GC analysis showed full conversion of **3a** and **3b** to γ-damascenone (**4**). Ethyl acetate (50 mL) was added; the mixture was washed with saturated aq. $NaHCO_3$ (50 mL × 2) and then 50 mL brine, dried over Na_2SO_4, filtered, and the solvent was removed by rotary evaporation. The crude mixture was purified by silica gel column chromatography, eluting with a 5:1 mixture of petroleum ether (bp 40–60 °C) and ethyl acetate to give γ-damascenone (**4**, 3.1 mg, 34%) as a colorless oil. The assignment of **4** as γ-damascenone was consistent with literature data.[8]

^{1}H NMR (600 MHz, $CDCl_3$) δ 6.87 (dd, *J* = 15.5, 7.0 Hz, 1H), 6.22 (dd, *J* = 15.5, 1.5 Hz, 1H), 6.20 – 6.15 (m, 1H), 5.86 – 5.83 (m, 1H), 5.02 (s, 1H), 4.93 – 4.87 (m, 1H), 3.23 (s, 1H), 2.44 – 2.37 (m, 1H), 1.87 (dd, *J* = 7.0, 1.5 Hz, 3H), 1.85 – 1.82 (m, 1H), 0.97 (s, 3H), 0.92 (s, 3H). **^{13}C NMR** (151 MHz, $CDCl_3$) δ 199.5, 142.8, 140.6, 131.6, 129.7, 127.2, 115.5, 61.6, 37.3, 32.6, 29.0, 27.4, 18.4. **HRMS** (ESI) $[M+H]^+$ Calcd for $C_{13}H_{19}O^+$: 191.1430, Found: 191.1427.

5a: 4,5-Epoxy-α-ionol

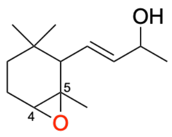

The 100 mL reaction mixture (200 mM potassium phosphate buffer, pH 7.9) in a 500 mL conical flask contained the $P450_{BM3}$ variant RP/H171L/I263G (2 μM), α-ionol (**5**) (97 mg, 5 mM added as a 200 mM solution stock in methanol), glucose dehydrogenase (GDH, 4 U/mL, 4 U/μL stock) and glucose (100 mM, 10 mL of a 1 M stock). The reaction was initiated by the addition of $NADP^+$ monosodium salt (40 μM, 1 mL of a 4.0 mM solution). After the flask was shaken at 120 rpm, 20 °C for 16 h, GC analysis showed 97% conversion of **5** with 54% selectivity for **5a**. The reaction mixture was extracted three times with 50 mL ethyl acetate; the combined extracts were washed with water and then brine, dried over Na_2SO_4 and the solvent was removed by rotary evaporation. The crude mixture was purified by silica gel column chromatography, eluting with a 5:1 mixture of petroleum ether (bp 40–60 °C) and ethyl acetate to give **5a** as a yellowish oil. The assignment of **5a** as 4,5-epoxy-α-ionol was consistent with literature data.[9]

^{1}H NMR (400 MHz, $CDCl_3$) δ 5.58 (ddd, J = 15.0, 6.0, 2.5 Hz, 1H), 5.54 – 5.41 (m, 1H), 4.41 – 4.26 (m, 1H), 2.96 (q, J = 2.0 Hz, 1H), 2.15 (d, J = 10.5 Hz, 1H), 2.01 – 1.93 (m, 1H), 1.91 – 1.79 (m, 1H), 1.34 (dt, J = 13.0, 6.5 Hz, 1H), 1.28 (d, J = 6.5 Hz, 3H), 1.17 (d, J = 14.0 Hz, 3H), 1.13 – 1.05 (m, 1H), 0.81 – 0.75 (m, 6H). **^{13}C NMR** (101 MHz, $CDCl_3$) δ 138.5 (d, J = 6.0 Hz), 127.7 (d, J = 5.5 Hz), 68.7 (d, J = 7.5 Hz), 60.2 (d, J = 1.5 Hz), 58.5 (d, J = 5.0 Hz), 53.7 (d, J = 2.5 Hz), 32.8 (d, J = 1.5 Hz), 31.2 (d, J = 5.0 Hz), 29.5 (d, J = 5.5 Hz), 23.7 (dd, J = 6.5, 4.0 Hz), 21.6, 21.2 (d, J = 3.5 Hz). **HRMS** (ESI) $[M+Na]^+$ Calcd for $C_{13}H_{22}NaO_2^+$: 233.1356, Found: 233.1355.

5b: *cis*-3-Hydroxy-α-ionol

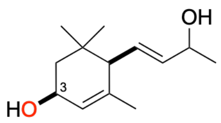

The 100 mL reaction mixture (200 mM potassium phosphate buffer, pH 7.9) in a 500 mL conical flask contained the P450$_{BM3}$ variant R19/F87A/S72W (2 μM), α-ionol (**5**) (97 mg, 5 mM added as a 200 mM solution stock in methanol), glucose dehydrogenase (GDH, 4 U/mL, 4 U/μL stock) and glucose (100 mM, 10 mL of a 1 M stock). The reaction was initiated by the addition of NADP$^+$ monosodium salt (40 μM, 1 mL of a 4.0 mM solution). After the flask was shaken at 120 rpm, 20 °C for 16 h, GC analysis showed 47% conversion of **5** with 45% selectivity for **5b**. The reaction mixture was extracted three times with 50 mL ethyl acetate; the combined extracts were washed with water and then brine, dried over Na_2SO_4 and the solvent was removed by rotary evaporation. The crude mixture was purified by silica gel column chromatography, eluting with a 5:1 mixture of petroleum ether (bp 40–60 °C) and ethyl acetate to give **5b** as a colorless oil. The assignment of **5b** as *cis*-3-hydroxy-α-ionol was consistent with literature data.[10]

^{1}H NMR (400 MHz, $CDCl_3$) δ 5.61 – 5.41 (m, 3H), 4.31 (q, *J* = 6.0 Hz, 1H), 4.25 – 4.16 (m, 1H), 2.07 (d, *J* = 8.5 Hz, 1H), 1.63 (dt, *J* = 6.5, 1.5 Hz, 4H), 1.39 – 1.32 (m, 1H), 1.27 (d, *J* = 6.5 Hz, 3H), 0.92 (s, 3H), 0.86 (s, 3H). **^{13}C NMR** (101 MHz, $CDCl_3$) δ 137.6, 136.4, 130.2, 124.7 (d, *J* = 3.5 Hz), 68.8, 66.7, 54.0, 40.8, 29.1 (d, *J* = 2.0 Hz), 27.0, 23.6 (d, *J* = 3.5 Hz), 22.4. **HRMS** (ESI) [M+Na]$^+$ Calcd for $C_{13}H_{22}NaO_2^+$: 233.1356, Found: 233.1355.

5c*:* *trans*-3-Hydroxy-α-ionol

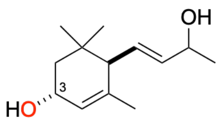

The 100 mL reaction mixture (200 mM potassium phosphate buffer, pH 7.9) in a 500 mL conical flask contained the $P450_{BM3}$ variant R19/F87I (2 μM), α-ionol (**5**) (97 mg, 5 mM added as a 200 mM solution stock in methanol), glucose dehydrogenase (GDH, 4 U/mL, 4 U/μL stock) and glucose (100 mM, 10 mL of a 1 M stock). The reaction was initiated by the addition of $NADP^+$ monosodium salt (40 μM, 1 mL of a 4.0 mM solution). After the flask was shaken at 120 rpm, 20 °C for 16 h, GC analysis showed 40% conversion of **5** with 62% selectivity for **5c**. The reaction mixture was extracted three times with 50 mL ethyl acetate; the combined extracts were washed with water and then brine, dried over Na_2SO_4 and the solvent was removed by rotary evaporation. The crude mixture was purified by silica gel column chromatography, eluting with a 5:1 mixture of petroleum ether (bp 40–60 °C) and ethyl acetate to give **5b** as a colorless oil. The assignment of **5c** as *trans*-3-hydroxy-α-ionol was consistent with literature data.[10, 11]

^{1}H NMR (400 MHz, $CDCl_3$) δ 5.61 – 5.52 (m, 2H), 5.36 (dddd, *J* = 15.5, 10.0, 3.5, 1.0 Hz, 1H), 4.32 (pd, *J* = 6.5, 1.0 Hz, 1H), 4.22 (dddd, *J* = 6.5, 6.0, 3.5, 2.0 Hz, 1H), 2.31 (dt, *J* = 10.0, 2.0 Hz, 1H), 1.81 (dd, *J* = 13.0, 6.0 Hz, 1H), 1.64 – 1.60 (m, 3H), 1.34 (dd, *J* = 13.5, 6.5 Hz, 1H), 1.28 (dt, *J* = 6.5, 1.5 Hz, 3H), 0.99 (d, *J* = 6.0 Hz, 3H), 0.83 (d, *J* = 8.0 Hz, 3H). **^{13}C NMR** (101 MHz, $CDCl_3$) δ 137.8, 137.6, 129.3 (d, *J* = 6.0 Hz), 124.8, 68.9, 66.0, 54.1, 44.6, 33.6, 29.5 (d, *J* = 3.5 Hz), 24.3 (d, *J* = 6.0 Hz), 23.8 (d, *J* = 5.5 Hz), 22.8 (d, *J* = 3.5 Hz). **HRMS** (ESI) $[M+Na]^+$ Calcd for $C_{13}H_{22}NaO_2^+$: 233.1517, Found: 233.1512.

5d*:* 13-Hydroxy-α-ionol

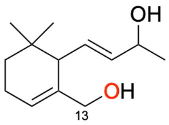

The 100 mL reaction mixture (200 mM potassium phosphate buffer, pH 7.9) in a 500 mL conical flask contained the $P450_{BM3}$ variant R19/A82M/I263G/A264G/A328G (2 μM), α-ionol (**5**) (97 mg, 5 mM added as a 200 mM solution stock in methanol), glucose dehydrogenase (GDH, 4 U/mL, 4 U/μL stock) and glucose (100 mM, 10 mL of a 1 M stock). The reaction was initiated by the addition of $NADP^+$ monosodium salt (40 μM, 1 mL of a 4.0 mM solution). After the flask was shaken at 120 rpm, 20 °C for 16 h, GC analysis showed 76% conversion of **5** with 70% selectivity for **5d**. The reaction mixture was extracted three times with 50 mL ethyl acetate; the combined extracts were washed with water and then brine, dried over Na_2SO_4 and the solvent was removed by rotary evaporation. The crude mixture was purified by silica gel column chromatography, eluting with a 5:1 mixture of petroleum ether (bp 40–60 °C) and ethyl acetate to give **5d** as a colorless oil.

^{1}H NMR (400 MHz, MeOD) δ 5.70 (t, J = 3.5 Hz, 1H), 5.58 – 5.41 (m, 2H), 4.23 (qd, J = 6.5, 3.5 Hz, 1H), 3.97 – 3.81 (m, 2H), 2.41 – 2.33 (m, 1H), 2.09 (dtq, J = 6.5, 3.5, 1.5 Hz, 2H), 1.58 – 1.48 (m, 1H), 1.23 (dd, J = 6.5, 1.0 Hz, 4H), 0.94 (d, J = 2.5 Hz, 3H), 0.87 (d, J = 10.0 Hz, 3H). **^{13}C NMR** (101 MHz, MeOD) δ 139.3 (d, J = 7.0 Hz), 137.5 (d, J = 9.0 Hz), 131.2 (d, J = 18.0 Hz), 123.1, 69.2 (d, J = 6.0 Hz), 65.7 (d, J = 5.5 Hz), 50.6 (d, J = 15.5 Hz), 32.7, 32.6 (d, J = 14.0 Hz), 28.3 (d, J = 12.0 Hz), 27.1 (d, J = 4.5 Hz), 23.9, 23.8. **HRMS** (ESI) $[M+H]^+$ Calcd for $C_{13}H_{23}O_2^+$: 211.1536, Found: 211.1537.

5e: 3-Oxo-α-ionol

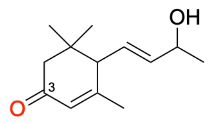

The 100 mL reaction mixture (200 mM potassium phosphate buffer, pH 7.9) in a 500 mL conical flask contained the P450$_{BM3}$ variant R19/F87A/S72W (2 μM), α-ionol (**5**) (97 mg, 5 mM added as a 200 mM solution stock in methanol), glucose dehydrogenase (GDH, 4 U/mL, 4 U/μL stock) and glucose (100 mM, 10 mL of a 1 M stock). The reaction was initiated by the addition of NADP$^+$ monosodium salt (40 μM, 1 mL of a 4.0 mM solution). After the flask was shaken at 120 rpm, 20 °C for 16 h, GC analysis showed 47% conversion of **5** with 16% selectivity for **5e**. The reaction mixture was extracted three times with 50 mL ethyl acetate; the combined extracts were washed with water and then brine, dried over Na_2SO_4 and the solvent was removed by rotary evaporation. The crude mixture was purified by silica gel column chromatography, eluting with a 5:1 mixture of petroleum ether (bp 40–60 °C) and ethyl acetate to give **5e** as a yellowish oil. The assignment of **5e** as 3-oxo-α-ionol was consistent with literature data.[11]

^{1}H NMR (500 MHz, $CDCl_3$) δ 5.90 (d, J = 1.5 Hz, 1H), 5.72 – 5.63 (m, 1H), 5.55 (dddd, J = 15.5, 9.0, 8.0, 1.0 Hz, 1H), 4.35 (p, J = 6.5 Hz, 1H), 2.52 (dd, J = 9.0, 3.5 Hz, 1H), 2.33 (dt, J = 16.5, 1.0 Hz, 1H), 2.08 (dq, J = 16.5, 1.0 Hz, 1H), 1.90 (dd, J = 7.0, 1.5 Hz, 3H), 1.29 (dd, J = 6.5, 3.0 Hz, 3H), 1.03 (d, J = 2.5 Hz, 3H), 0.96 (d, J = 9.0 Hz, 3H). **^{13}C NMR** (126 MHz, $CDCl_3$) δ 199.3 (d, J = 2.5 Hz), 162.0 (d, J = 7.5 Hz), 138.7 (d, J = 15.5 Hz), 126.8 (d, J = 5.0 Hz), 126.0 (d, J = 8.0 Hz), 68.5 (d, J = 7.5 Hz), 55.6 (d, J = 8.5 Hz), 47.6 (d, J = 7.5 Hz), 36.3 (d, J = 2.5 Hz), 28.0, 27.3 (d, J = 2.5 Hz), 23.8 (d, J = 5.5 Hz), 23.7 (d, J = 4.0 Hz). **HRMS** (ESI) $[M+Na]^+$ Calcd for $C_{13}H_{20}NaO_2^+$: 231.1512, Found: 231.1349.

5f: 3-Oxo-13-hydroxy-α-ionol

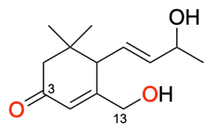

The 100 mL reaction mixture (200 mM potassium phosphate buffer, pH 7.9) in a 500 mL conical flask contained the $P450_{BM3}$ variant R19/F87A/A184I (2 μM), α-ionol (**5**) (97 mg, 5 mM added as a 200 mM solution stock in methanol), glucose dehydrogenase (GDH, 4 U/mL, 4 U/μL stock) and glucose (100 mM, 10 mL of a 1 M stock). The reaction was initiated by the addition of $NADP^+$ monosodium salt (40 μM, 1 mL of a 4.0 mM solution). After the flask was shaken at 120 rpm, 20 °C for 16 h, GC analysis showed 100% conversion of **5** with 34% selectivity for **5f**. The reaction mixture was extracted three times with 50 mL ethyl acetate; the combined extracts were washed with water and then brine, dried over Na_2SO_4 and the solvent was removed by rotary evaporation. The crude mixture was purified by silica gel column chromatography, eluting with a 5:1 mixture of petroleum ether (bp 40–60 °C) and ethyl acetate to give **5f** as a yellowish oil. The assignment of **5f** as 3-oxo-13-hydroxy-α-ionol was consistent with literature data.[12]

^{1}H NMR (400 MHz, $CDCl_3$) δ 6.20 – 6.13 (m, 1H), 5.69 – 5.61 (m, 1H), 5.60 – 5.49 (m, 1H), 4.32 (q, *J* = 6.0 Hz, 1H), 4.19 (dt, *J* = 20.5, 16.5 Hz, 2H), 2.56 (t, *J* = 8.5 Hz, 1H), 2.40 (dd, *J* = 17.0, 1.5 Hz, 1H), 2.12 (ddt, *J* = 17.0, 4.5, 1.0 Hz, 1H), 1.27 (dd, *J* = 6.5, 2.0 Hz, 3H), 1.02 (d, *J* = 3.5 Hz, 3H), 0.97 (d, *J* = 8.0 Hz, 3H). **^{13}C NMR** (101 MHz, $CDCl_3$) δ 199.7, 164.6, 138.8 (d, *J* = 21.5 Hz), 126.8, 122.3 (d, *J* = 10.5 Hz), 68.4, 63.9 (d, *J* = 6.5 Hz), 51.2, 48.2 (d, *J* = 12.0 Hz), 36.3, 27.8, 27.2 (d, *J* = 3.5 Hz), 23.7 (d, *J* = 2.5 Hz). **LRMS** (ESI) $[M+Na]^+$ Calcd for $C_{13}H_{20}NaO_3^+$: 247.2, Found: 247.2.

6a: 4,5-Epoxy-α-ionone

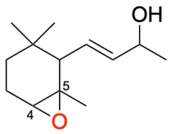

The 100 mL reaction mixture (200 mM potassium phosphate buffer, pH 7.9) in a 500 mL conical flask contained the $P450_{BM3}$ variant RT2/I263A/A330W (2 μM), α-ionone (**6**) (95 mg, 5 mM added as a 200 mM solution stock in methanol), glucose dehydrogenase (GDH, 4 U/mL, 4 U/μL stock) and glucose (100 mM, 10 mL of a 1 M stock). The reaction was initiated by the addition of $NADP^+$ monosodium salt (40 μM, 1 mL of a 4.0 mM solution). After the flask was shaken at 120 rpm, 20 °C for 16 h, GC analysis showed 50% conversion of **6** with 76% selectivity for **6a**. The reaction mixture was extracted three times with 50 mL ethyl acetate; the combined extracts were washed with water and then brine, dried over Na_2SO_4 and the solvent was removed by rotary evaporation. The crude mixture was purified by silica gel column chromatography, eluting with a 5:1 mixture of petroleum ether (bp 40–60 °C) and ethyl acetate to give **6a** as a yellowish oil. The assignment of **6a** as 4,5-epoxy-α-ionone was consistent with literature data.[14]

^{1}H NMR (500 MHz, $CDCl_3$) δ 6.63 (dd, J = 15.5, 11.5 Hz, 1H), 6.11 (d, J = 15.5 Hz, 1H), 2.97 (t, J = 2.0 Hz, 1H), 2.30 (d, J = 11.5 Hz, 1H), 2.25 (s, 3H), 1.99 (ddt, J = 15.5, 5.5, 2.0 Hz, 1H), 1.90 – 1.83 (m, 1H), 1.40 – 1.29 (m, 1H), 1.16 (s, 3H), 1.10 (ddd, J = 13.5, 6.0, 2.5 Hz, 1H), 0.82 (s, 3H), 0.76 (s, 3H). **^{13}C NMR** (126 MHz, $CDCl_3$) δ 197.8, 145.2, 134.2, 59.8, 57.9, 54.1, 32.6, 31.8, 29.6, 27.8, 23.6, 21.5, 21.5. **HRMS** (ESI) $[M+Na]^+$ Calcd for $C_{13}H_{20}NaO_2^+$: 231.1356, Found: 231.1351.

6b: *cis*-3-Hydroxy-α-ionone

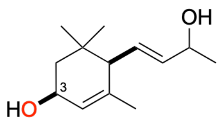

The 100 mL reaction mixture (200 mM potassium phosphate buffer, pH 7.9) in a 500 mL conical flask contained the $P450_{BM3}$ variant R19/F87A/A328L (2 μM), α-ionone (**6**) (95 mg, 5 mM added as a 200 mM solution stock in methanol), glucose dehydrogenase (GDH, 4 U/mL, 4 U/μL stock) and glucose (100 mM, 10 mL of a 1 M stock). The reaction was initiated by the addition of $NADP^+$ monosodium salt (40 μM, 1 mL of a 4.0 mM solution). After the flask was shaken at 120 rpm, 20 °C for 16 h, GC analysis showed 91% conversion of **6** with 70% selectivity for **6b**. The reaction mixture was extracted three times with 50 mL ethyl acetate; the combined extracts were washed with water and then brine, dried over Na_2SO_4 and the solvent was removed by rotary evaporation. The crude mixture was purified by silica gel column chromatography, eluting with a 5:1 mixture of petroleum ether (bp 40–60 °C) and ethyl acetate to give **6b** as a yellowish oil. The assignment of **6b** as *cis*-3-hydroxy-α-ionone was consistent with literature data.[13]

^{1}H NMR (500 MHz, $CDCl_3$) δ 6.62 (dd, J = 16.0, 9.5 Hz, 1H), 6.06 (dd, J = 16.0, 1.0 Hz, 1H), 5.57 (dq, J = 2.5, 1.5 Hz, 1H), 4.23 (tdd, J = 8.0, 4.5, 2.0 Hz, 1H), 2.24 (m, 4H), 1.67 (ddt, J = 13.0, 6.5, 1.5 Hz, 1H), 1.61 (t, J = 1.5 Hz, 3H), 1.38 (dd, J = 13.0, 10.0 Hz, 1H), 0.95 (s, 3H), 0.86 (s, 3H). **^{13}C NMR** (126 MHz, $CDCl_3$) δ 198.6, 148.0, 135.4, 132.8, 126.6, 66.5, 54.4, 40.7, 35.1, 29.2, 27.2, 27.1, 22.5. **HRMS** (ESI) $[M+Na]^+$ Calcd for $C_{13}H_{20}NaO_2^+$: 231.1356, Found: 231.1351.

6c: *trans*-3-Hydroxy-α-ionone

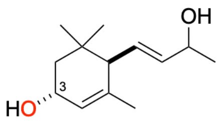

The 100 mL reaction mixture (200 mM potassium phosphate buffer, pH 7.9) in a 500 mL conical flask contained the $P450_{BM3}$ variant R19/A184I/T260G (2 μM), α-ionone (**6**) (95 mg, 5 mM added as a 200 mM solution stock in methanol), glucose dehydrogenase (GDH, 4 U/mL, 4 U/μL stock) and glucose (100 mM, 10 mL of a 1 M stock). The reaction was initiated by the addition of $NADP^+$ monosodium salt (40 μM, 1 mL of a 4.0 mM solution). After the flask was shaken at 120 rpm, 20 °C for 16 h, GC analysis showed 46% conversion of **6** with 58% selectivity for **6c**. The reaction mixture was extracted three times with 50 mL ethyl acetate; the combined extracts were washed with water and then brine, dried over Na_2SO_4 and the solvent was removed by rotary evaporation. The crude mixture was purified by silica gel column chromatography, eluting with a 5:1 mixture of petroleum ether (bp 40–60 °C) and ethyl acetate to give **6c** as a yellowish oil. The assignment of **6c** as *trans*-3-hydroxy-α-ionone was consistent with literature data.[13]

^{1}H NMR (500 MHz, $CDCl_3$) δ 6.55 – 6.47 (m, 1H), 6.07 (d, J = 16.0 Hz, 1H), 5.60 (dt, J = 3.0, 1.5 Hz, 1H), 4.27 – 4.20 (m, 1H), 2.47 (d, J = 10.0 Hz, 1H), 2.23 (s, 3H), 1.80 (ddd, J = 13.5, 6.0, 1.5 Hz, 1H), 1.61 – 1.56 (m, 3H), 1.37 (dd, J = 13.5, 6.5 Hz, 1H), 0.99 (s, 3H), 0.85 (s, 3H). **^{13}C NMR** (126 MHz, $CDCl_3$) δ 198.2, 147.4, 135.3, 133.7, 126.0, 65.4, 54.3, 43.9, 34.0, 29.4, 27.2, 24.7, 22.7. **HRMS** (ESI) $[M+Na]^+$ Calcd for $C_{13}H_{20}NaO_2^+$: 231.1356, Found: 231.1350.

6d: 3-Oxo-α-ionone

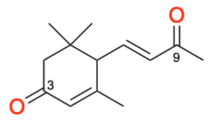

The 100 mL reaction mixture (200 mM potassium phosphate buffer, pH 7.9) in a 500 mL conical flask contained the P450$_{BM3}$ variant R19/F87A/A184I/S270G (2 μM), α-ionone (**6**) (95 mg, 5 mM added as a 200 mM solution stock in methanol), glucose dehydrogenase (GDH, 4 U/mL, 4 U/μL stock) and glucose (100 mM, 10 mL of a 1 M stock). The reaction was initiated by the addition of NADP$^+$ monosodium salt (40 μM, 1 mL of a 4.0 mM solution). After the flask was shaken at 120 rpm, 20 °C for 16 h, GC analysis showed 100% conversion of **6** with 48% selectivity for **6d**. The reaction mixture was extracted three times with 50 mL ethyl acetate; the combined extracts were washed with water and then brine, dried over Na_2SO_4 and the solvent was removed by rotary evaporation. The crude mixture was purified by silica gel column chromatography, eluting with a 5:1 mixture of petroleum ether (bp 40–60 °C) and ethyl acetate to give **6d** as a yellowish oil. The assignment of **6d/5g** as 3-oxo-α-ionone was consistent with literature data.[13]

^{1}H NMR (400 MHz, $CDCl_3$) δ 6.67 (dd, *J* = 16.0, 9.5 Hz, 1H), 6.18 (d, *J* = 16.0 Hz, 1H), 5.98 (d, *J* = 1.5 Hz, 1H), 2.71 (d, *J* = 10.0 Hz, 1H), 2.39 – 2.33 (m, 1H), 2.28 (s, 3H), 2.15 (d, *J* = 17.0 Hz, 1H), 1.89 (d, *J* = 1.5 Hz, 3H), 1.08 (s, 3H), 1.00 (s, 3H). **^{13}C NMR** (101 MHz, $CDCl_3$) δ 198.4, 197.7, 159.3, 143.7, 133.9, 127.1, 55.6, 47.5, 36.8, 28.1, 27.7, 27.5, 23.7. **HRMS** (ESI) [M+Na]$^+$ Calcd for $C_{13}H_{19}O_2^+$: 207.1380, Found: 207.1376.

6e: 1-(7,7-Dimethyl-1,3,5,6,7,7a-hexahydroisobenzofuran-1-yl) propan-2-one (7,13-furan)

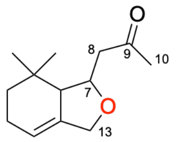

The 100 mL reaction mixture (200 mM potassium phosphate buffer, pH 7.9) in a 500 mL conical flask contained the $P450_{BM3}$ variant R19/A82M/I263G/A328G (2 μM), α-ionone (**6**) (95 mg, 5 mM added as a 200 mM solution stock in methanol), glucose dehydrogenase (GDH, 4 U/mL, 4 U/μL stock) and glucose (100 mM, 10 mL of a 1 M stock). The reaction was initiated by the addition of $NADP^+$ monosodium salt (40 μM, 1 mL of a 4.0 mM solution). After the flask was shaken at 120 rpm, 20 °C for 16 h, GC analysis showed 45% conversion of **6** with 28% selectivity for **6e**. The reaction mixture was extracted three times with 50 mL ethyl acetate; the combined extracts were washed with water and then brine, dried over Na_2SO_4 and the solvent was removed by rotary evaporation. The crude mixture was purified by silica gel column chromatography, eluting with a 5:1 mixture of petroleum ether (bp 40–60 °C) and ethyl acetate to give **6e** as a yellowish oil.

^{1}H NMR (500 MHz, $CDCl_3$) δ 5.47 (h, J = 2.5 Hz, 1H), 4.37 (ddq, J = 12.5, 3.0, 1.5 Hz, 1H), 4.17 (dp, J = 12.5, 2.0 Hz, 1H), 4.01 (ddd, J = 9.5, 7.5, 4.5 Hz, 1H), 2.75 – 2.72 (m, 2H), 2.23 (s, 3H), 2.08 – 2.03 (m, 3H), 1.42 – 1.31 (m, 2H), 1.02 (s, 3H), 0.81 (s, 3H). **^{13}C NMR** (126 MHz, $CDCl_3$) δ 207.4, 138.8, 116.0, 76.6, 69.6, 54.3, 50.4, 37.8, 31.1, 30.6, 30.3, 23.1, 19.4. **HRMS** (ESI) $[M+H]^+$ Calcd for $C_{13}H_{21}O_2^+$: 209.1536, Found: 209.1537.

7: (*E*/*E*)-Megastigmatriene

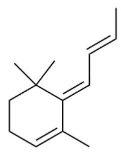

β-Ionone (250 mg) was dissolved in 20 mL THF with 20 mg of $NaBH_4$ and stirred for 30 minutes. The formation of (*E*/*E*)-megastigmatriene (**7**) was completed after the addition of 5 mL of 1 M HCl and stirring for a further 3 h, achieving 99% conversion and 84% yield. The assignment of **7** as (*E*/*E*)-megastigmatriene was consistent with literature data.[15]

^{1}H NMR (400 MHz, MeOD) δ 6.71 (ddq, *J* = 15.0, 11.5, 1.5 Hz, 1H), 6.03 (d, *J* = 11.5 Hz, 1H), 5.76 – 5.57 (m, 2H), 2.08 (tdd, *J* = 6.0, 4.0, 2.0 Hz, 2H), 1.80 (dq, *J* = 5.0, 1.5 Hz, 6H), 1.47 (t, *J* = 6.0 Hz, 2H), 1.25 (s, 6H). **^{13}C NMR** (101 MHz, MeOD) δ 143.4, 135.0, 131.3, 131.0, 127.8, 125.4, 41.7, 35.8, 29.4, 23.7, 21.9, 18.7. **HRMS** (ESI) $[M+H]^+$ Calcd for $C_{13}H_{21}^+$: 177.1638, Found: 177.1639.

7a: 4-Hydroxy-β-ionol

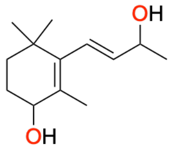

The 100 mL reaction mixture (200 mM potassium phosphate buffer, pH 7.9) in a 500 mL conical flask contained the P450$_{BM3}$ variant R19/F87I (2 μM), (*E*/*E*)-megastigmatriene (**7**) (88 mg, 5 mM added as a 200 mM solution stock in methanol), glucose dehydrogenase (GDH, 4 U/mL, 4 U/μL stock) and glucose (100 mM, 10 mL of a 1 M stock). The reaction was initiated by the addition of $NADP^+$ monosodium salt (40 μM, 1 mL of a 4.0 mM solution). After the flask was shaken at 120 rpm, 20 °C for 16 h, GC analysis showed 95% conversion of **7** with 34% selectivity for **7a**. The reaction mixture was extracted three times with 50 mL ethyl acetate; the combined extracts were washed with water and then brine, dried over Na_2SO_4 and the solvent was removed by rotary evaporation. The crude mixture was purified by silica gel column chromatography, eluting with a 5:1 mixture of petroleum ether (bp 40–60 °C) and ethyl acetate to give **7a** as a yellowish oil. The assignment of **7a** as 4-hydroxy-β-ionol was consistent with literature data.[16]

^{1}H NMR (500 MHz, $CDCl_3$) δ 6.02 (d, *J* = 16.0 Hz, 1H), 5.52 (dd, *J* = 16.0, 6.5 Hz, 1H), 4.37 (p, *J* = 6.5 Hz, 1H), 3.97 (d, *J* = 5.0 Hz, 1H), 1.87 (tdd, *J* = 11.5, 5.0, 2.5 Hz, 1H), 1.78 (s, 3H), 1.65 – 1.58 (m, 2H), 1.40 (ddd, *J* = 13.5, 7.5, 3.0 Hz, 1H), 1.31 (d, *J* = 6.5 Hz, 3H), 1.00 (d, *J* = 2.5 Hz, 3H), 0.97 (d, *J* = 2.0 Hz, 3H). **^{13}C NMR** (126 MHz, $CDCl_3$) δ 141.0, 138.8, 129.6 (d, *J* = 2.5 Hz), 126.7 (d, *J* = 3.0 Hz), 70.1, 69.3, 34.6, 34.5, 29.0, 28.6 (d, *J* = 1.5 Hz), 27.3, 23.7, 18.4. **HRMS** (ESI) $[M+H]^+$ Calcd for $C_{13}H_{22}NaO_2^+$: 233.1512, Found: 233.1510.

7b: 4-Oxo-β-ionol

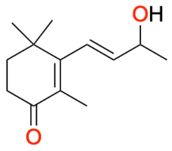

The 100 mL reaction mixture (200 mM potassium phosphate buffer, pH 7.9) in a 500 mL conical flask contained the P450$_{BM3}$ variant R19/F87I (2 μM), (*E*/*E*)-megastigmatriene (**7**) (88 mg, 5 mM added as a 200 mM solution stock in methanol), glucose dehydrogenase (GDH, 4 U/mL, 4 U/μL stock) and glucose (100 mM, 10 mL of a 1 M stock). The reaction was initiated by the addition of NADP$^+$ monosodium salt (40 μM, 1 mL of a 4.0 mM solution). After the flask was shaken at 120 rpm, 20 °C for 16 h, GC analysis showed 95% conversion of **7** with 10% selectivity for **7b**. The reaction mixture was extracted three times with 50 mL ethyl acetate; the combined extracts were washed with water and then brine, dried over Na_2SO_4 and the solvent was removed by rotary evaporation. The crude mixture was purified by silica gel column chromatography, eluting with a 5:1 mixture of petroleum ether (bp 40–60 °C) and ethyl acetate to give **7b** as a yellowish oil. The assignment of **7b** as 4-oxo-β-ionol was consistent with literature data.[16]

^{1}H NMR (500 MHz, $CDCl_3$) δ 6.22 (dt, *J* = 16.0, 1.0 Hz, 1H), 5.70 (dd, *J* = 16.0, 6.0 Hz, 1H), 4.50 – 4.41 (m, 1H), 2.50 (dd, *J* = 7.5, 6.5 Hz, 2H), 1.84 (dd, *J* = 7.5, 6.5 Hz, 2H), 1.80 (d, *J* = 1.0 Hz, 3H), 1.36 (d, *J* = 6.5 Hz, 3H), 1.15 (d, *J* = 2.0 Hz, 6H). **^{13}C NMR** (126 MHz, $CDCl_3$) δ 199.5, 160.7, 140.5, 130.2, 125.4, 68.8, 37.4, 35.6, 34.4, 27.5, 23.7, 13.5. **HRMS** (ESI) $[M+H]^+$ Calcd for $C_{13}H_{20}NaO_2^+$: 231.1356, Found: 231.1351.

7c: 3,4-Dihydroxy-β-ionol

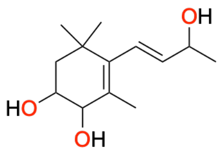

The 100 mL reaction mixture (200 mM potassium phosphate buffer, pH 7.9) in a 500 mL conical flask contained the $P450_{BM3}$ variant R19/F87I (2 μM), (*E*/*E*)-megastigmatriene (**7**) (88 mg, 5 mM added as a 200 mM solution stock in methanol), glucose dehydrogenase (GDH, 4 U/mL, 4 U/μL stock) and glucose (100 mM, 10 mL of a 1 M stock). The reaction was initiated by the addition of $NADP^+$ monosodium salt (40 μM, 1 mL of a 4.0 mM solution). After the flask was shaken at 120 rpm, 20 °C for 16 h, GC analysis showed 95% conversion of **7** with 28% selectivity for **7c**. The reaction mixture was extracted three times with 50 mL ethyl acetate; the combined extracts were washed with water and then brine, dried over Na_2SO_4 and the solvent was removed by rotary evaporation. The crude mixture was purified by silica gel column chromatography, eluting with a 5:1 mixture of petroleum ether (bp 40–60 °C) and ethyl acetate to give **7c** as a yellowish oil. The assignment of **7c** as 3,4-dihydroxy-β-ionol was consistent with literature data.[17]

^{1}H NMR (400 MHz, $CDCl_3$) δ 6.01 (d, *J* = 16.0 Hz, 1H), 5.55 (dd, *J* = 16.0, 6.5 Hz, 1H), 4.38 (tt, *J* = 6.5, 1.0 Hz, 1H), 3.92 (d, *J* = 4.0 Hz, 1H), 3.84 (dt, *J* = 12.5, 4.0 Hz, 1H), 1.84 (t, *J* = 1.5 Hz, 3H), 1.70 – 1.60 (m, 2H), 1.53 (ddd, *J* = 12.5, 4.0, 1.5 Hz, 1H), 1.31 (d, *J* = 6.5 Hz, 3H), 1.06 – 1.01 (m, 6H). **^{13}C NMR** (101 MHz, $CDCl_3$) δ 141.9, 139.3 (d, *J* = 5.0 Hz), 127.5, 126.0 (d, *J* = 4.0 Hz), 71.6, 69.1 (d, *J* = 5.0 Hz), 66.9, 41.3, 36.9, 30.0 (d, *J* = 3.0 Hz), 27.4, 23.7, 19.6. **HRMS** (ESI) $[M+H]^+$ Calcd for $C_{13}H_{22}NaO_3^+$: 249.1451, Found: 249.1454.

8: Megastigma-4,7*E*,9-triene-3-one (MEG2)

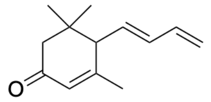

3-Oxo-α-ionol (**5e**, 29.6 mg) was heated with oxalic acid (6 equiv.) in butanol (4 mL) at 80 °C for 1 h and was fully converted to the terminal dehydration product megastigma-4,7*E*,9-triene-3-one (MEG2, **8**). Ethyl acetate (50 mL) was added to the mixture, which was washed twice with 50 mL of saturated aq. $NaHCO_3$ and then 50 mL brine, dried over Na_2SO_4, filtered, and the solvent was removed by rotary evaporation. The crude mixture was purified by silica gel column chromatography, eluting with a 5:1 mixture of petroleum ether (bp 40–60 °C) and ethyl acetate to give **8** (15.9 mg, 59%) as a yellowish oil. The assignment of **8** as megastigma-4,7*E*,9-triene-3-one (MEG2) was consistent with literature data.[18]

^{1}H NMR (500 MHz, MeOD) δ 6.42 (dt, *J* = 17.0, 10.5 Hz, 1H), 6.26 (dd, *J* = 15.0, 10.5 Hz, 1H), 5.91 (dq, *J* = 2.0, 1.0 Hz, 1H), 5.68 (dd, *J* = 15.0, 10.0 Hz, 1H), 5.27 – 5.20 (m, 1H), 5.13 – 5.06 (m, 1H), 2.73 (d, *J* = 9.5 Hz, 1H), 2.43 (d, *J* = 17.0 Hz, 1H), 2.12 – 2.02 (m, 1H), 1.96 (d, *J* = 1.5 Hz, 3H), 1.06 (s, 3H), 1.00 (s, 3H). **^{13}C NMR** (126 MHz, MeOD) δ 202.0, 165.7, 137.7, 136.3, 132.1, 126.1, 117.5, 57.1, 48.4, 37.4, 28.0, 27.4, 23.7. **HRMS** (ESI) $[M+H]^+$ Calcd for $C_{13}H_{19}O^+$: 191.1430, Found: 191.1431.

9-12: Megastigma-4,6*Z*,8*E*-triene-3-one (**MEG1**), megastigma-4,6*E*,8*E*-triene-3-one (**MEG3**), megastigma-4,6*E*,8*Z*-trien-3-one (**MEG4**), and megastigma-4,6*Z*,8*Z*-trien-3-one (**MEG5**)

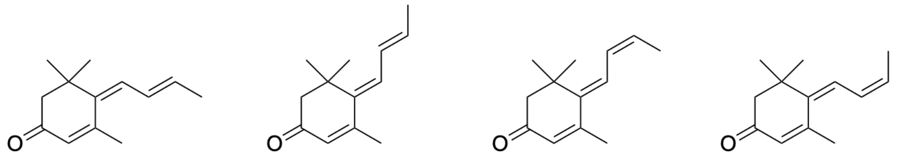

9, *Z*/*E* (MEG1) **10**, *E*/*E* (MEG3) **11**, *E*/*Z* (MEG4) **12**, *Z*/*Z* (MEG5)

Megastigma-4,7*E*,9-triene-3-one (MEG2, **8**, 15.9 mg) was treated with DBU (3 equiv.) at 40 °C in dimethoxyethane (10 mL) for 16 h and was fully converted to the mixture of **9**–**12**. The reaction mixture was quenched using 10 mL saturated NH_4Cl and extracted with dichloromethane followed by washing with 50 mL brine, dried over Na_2SO_4, filtered, and the solvent was removed by rotary evaporation. The crude mixture was purified by silica gel column chromatography, eluting with dichloromethane to give **9**–**12** (2.2 mg, 14%) as a yellowish oil. The assignments of **9**–**12** were consistent with literature data.[18]

^{1}H NMR (400 MHz, $CDCl_3$) δ 6.76 (ddq, *J* = 15.0, 11.5, 1.5 Hz, 1H), 6.60 – 6.44 (m, 1H), 6.01 – 5.82 (m, 3H), 2.36 (s, 2H), 2.32 – 2.24 (m, 3H), 2.08 (d, *J* = 1.0 Hz, 3H), 1.87 (ddd, *J* = 16.0, 7.0, 1.5 Hz, 5H), 1.35 (s, 6H), 1.19 (s, 3H). **^{13}C NMR** (151 MHz, $CDCl_3$) δ 199.4, 199.2, 155.6, 155.4, 140.9, 140.0, 137.2, 135.0, 132.8, 129.8, 129.2, 128.8, 128.6, 126.1, 54.2, 52.7, 40.6, 38.5, 30.0, 29.8, 28.3, 25.4, 22.5, 19.1, 18.8. **HRMS** (ESI) $[M+H]^+$ Calcd for $C_{13}H_{19}O^+$: 191.1430, Found: 191.1430.

1a – ^{1}H NMR (400 MHz, $CDCl_3$)

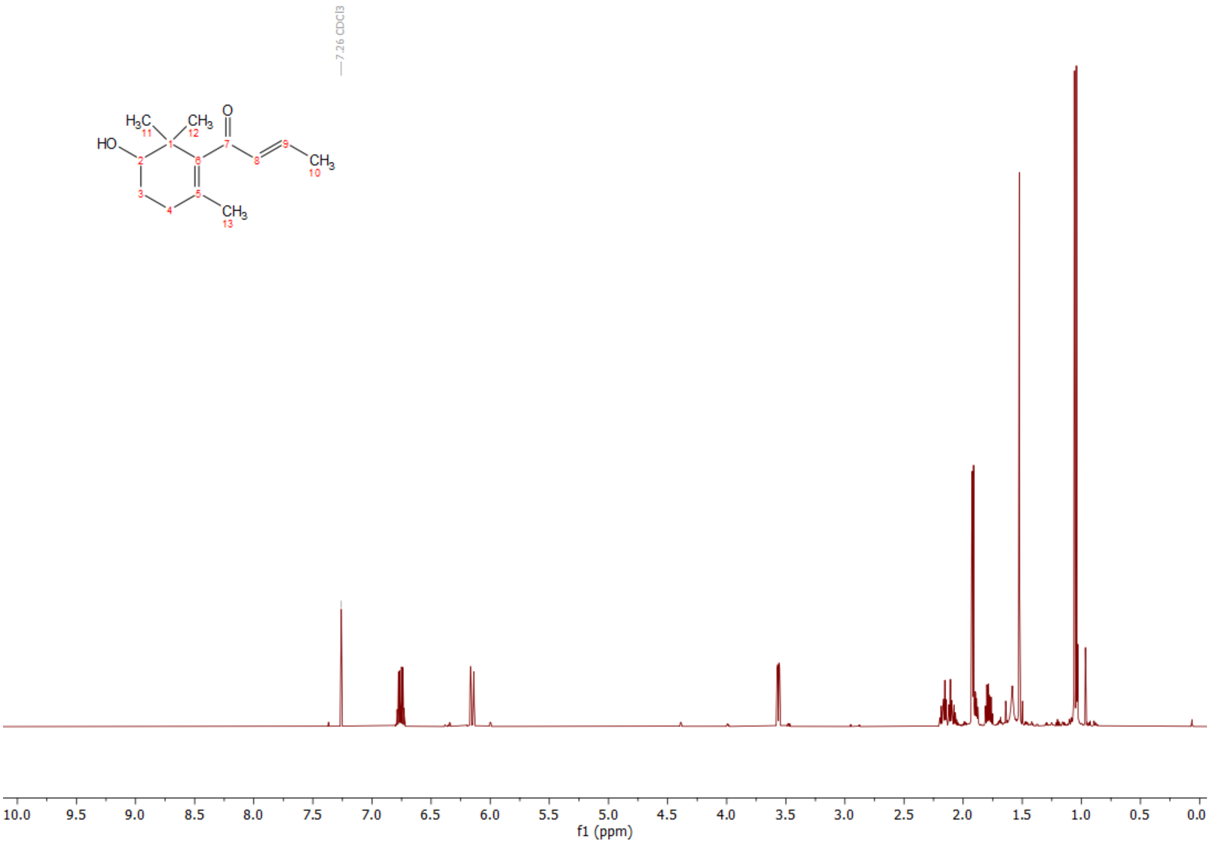

1a – ^{13}C NMR (101 MHz, $CDCl_3$)

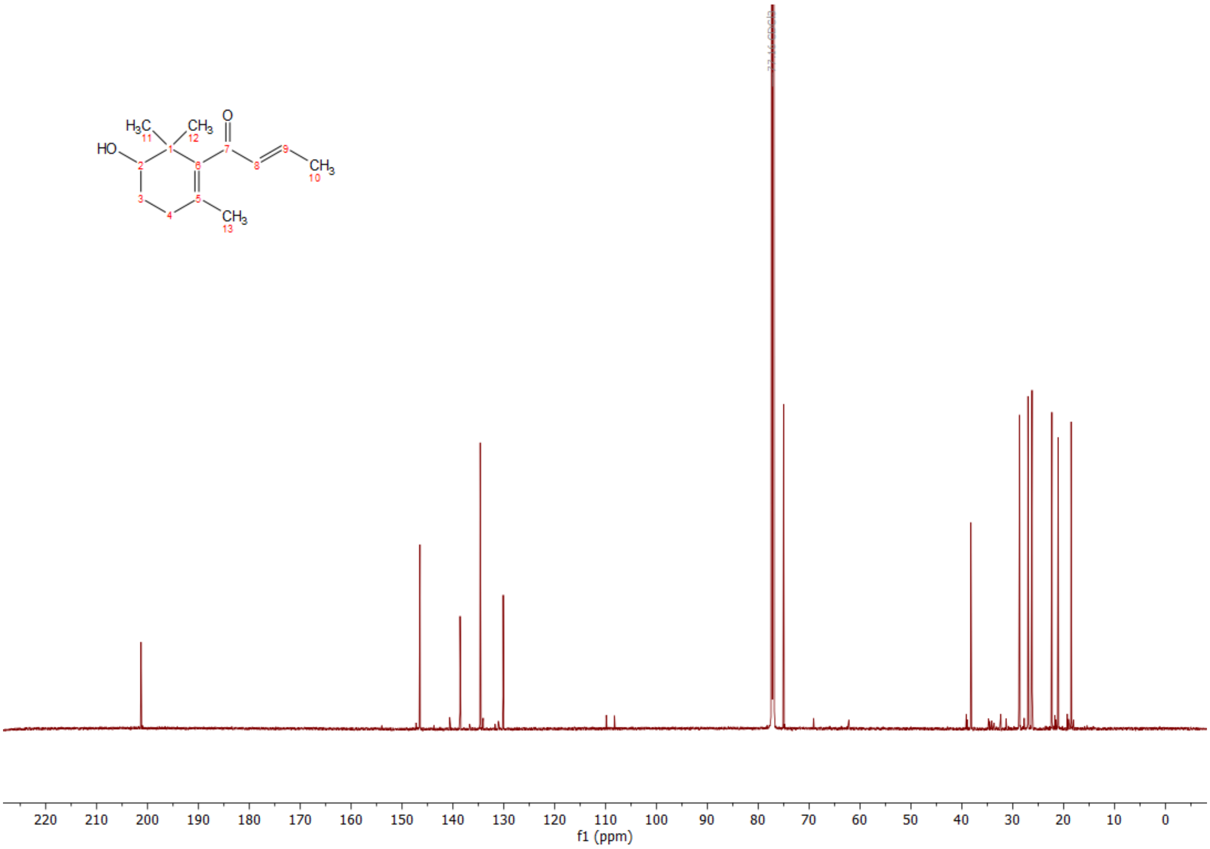

1b – ^{1}H NMR (500 MHz, $CDCl_3$)

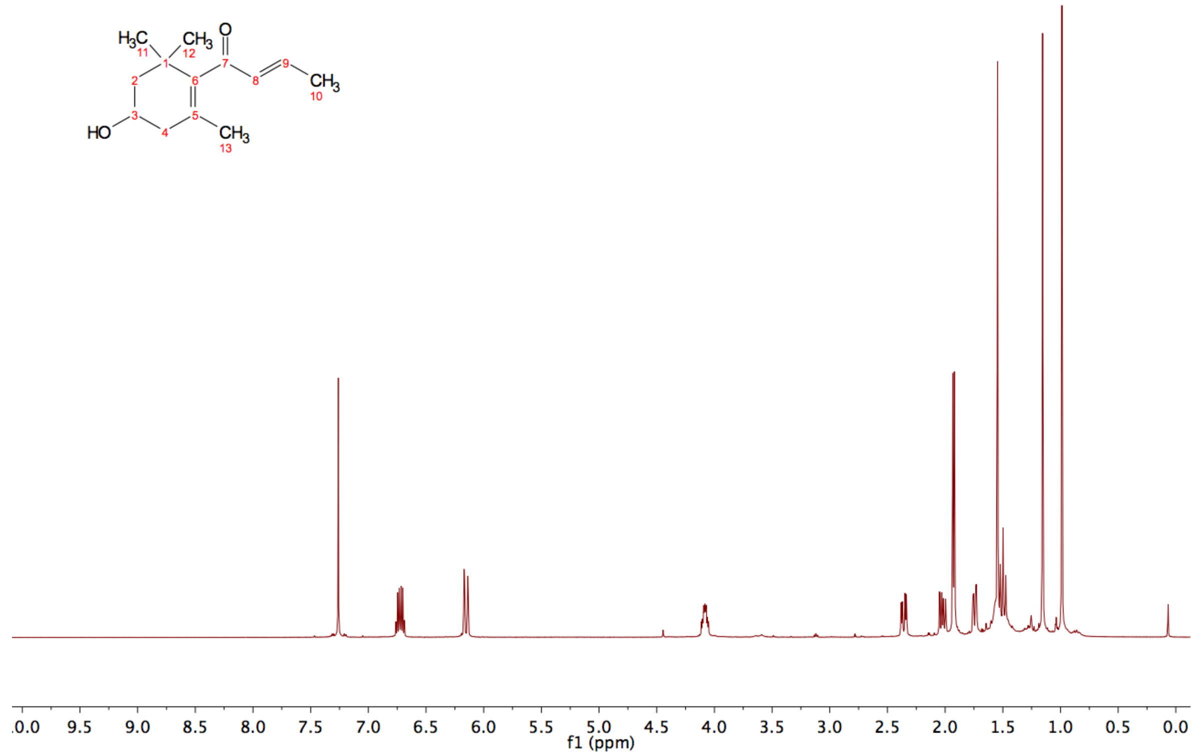

1b – ^{13}C NMR (126 MHz, $CDCl_3$)

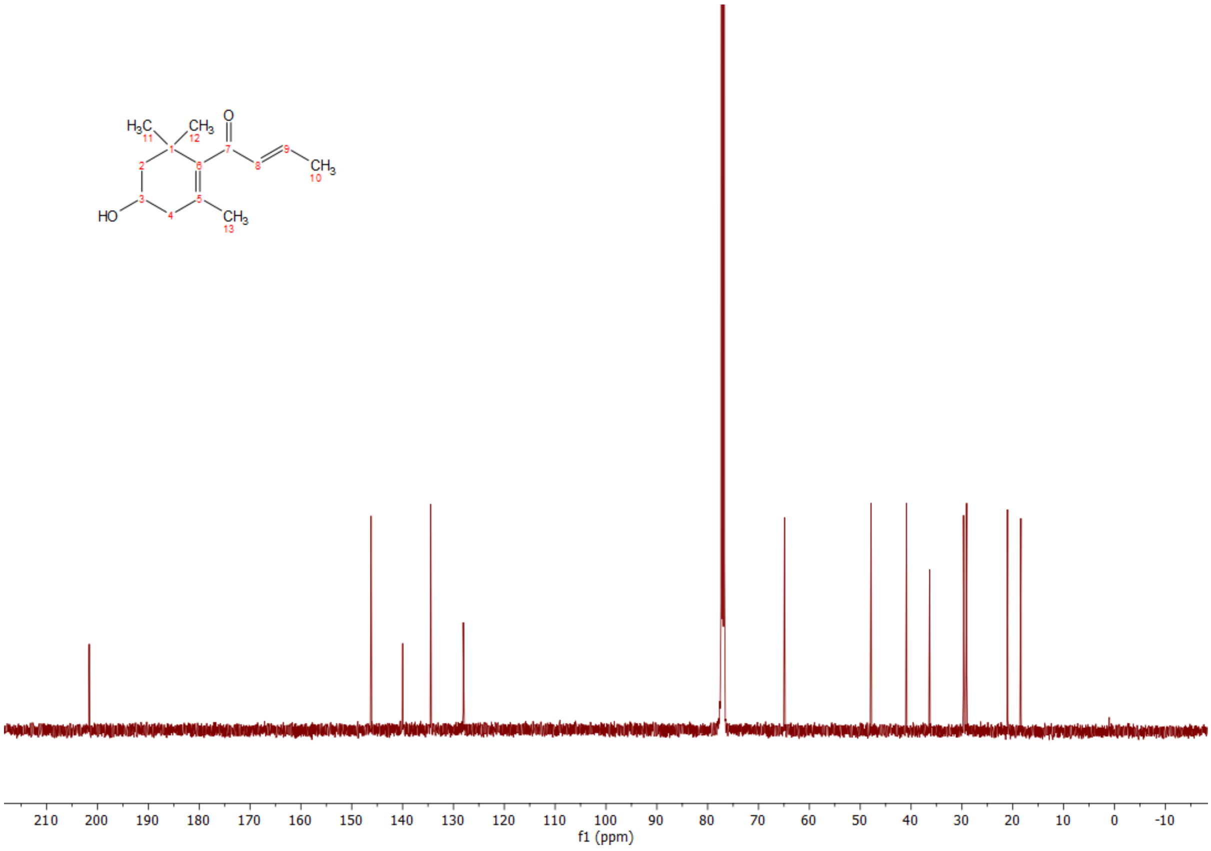

1c – ^{1}H NMR (400 MHz, $CDCl_3$)

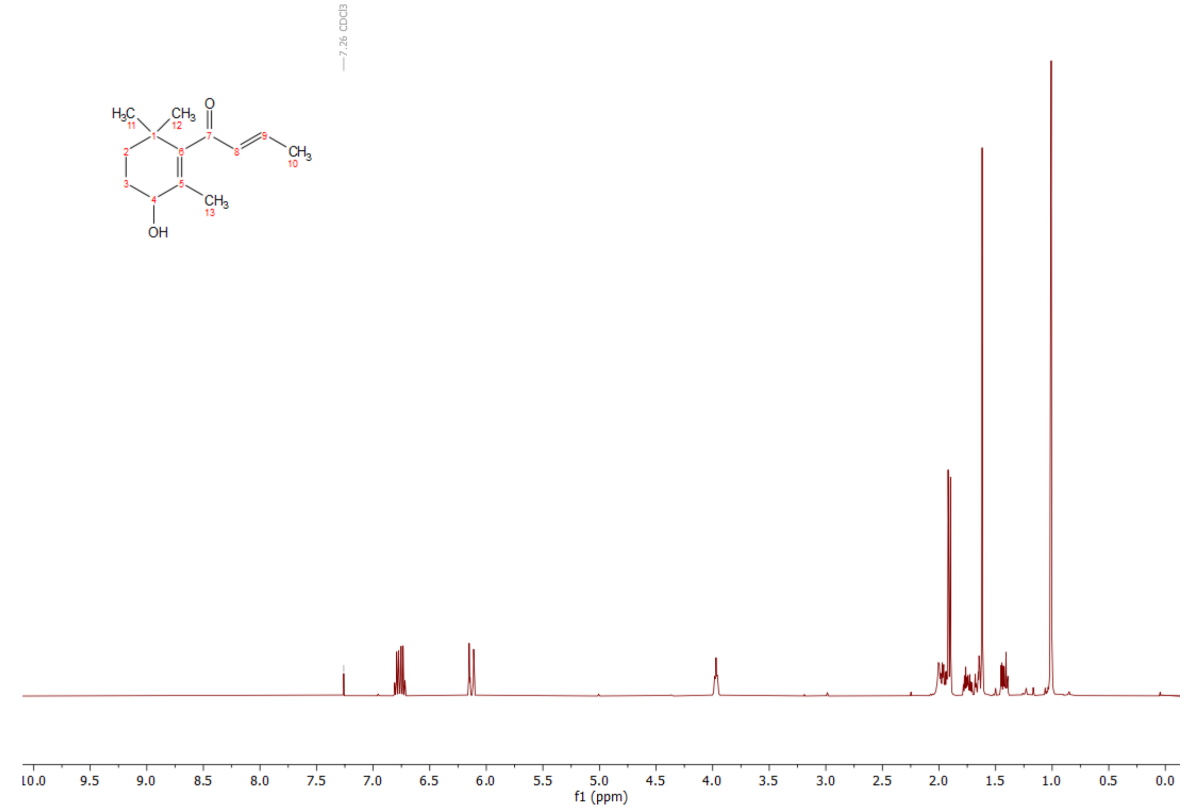

1c – ^{13}C NMR (101 MHz, $CDCl_3$)

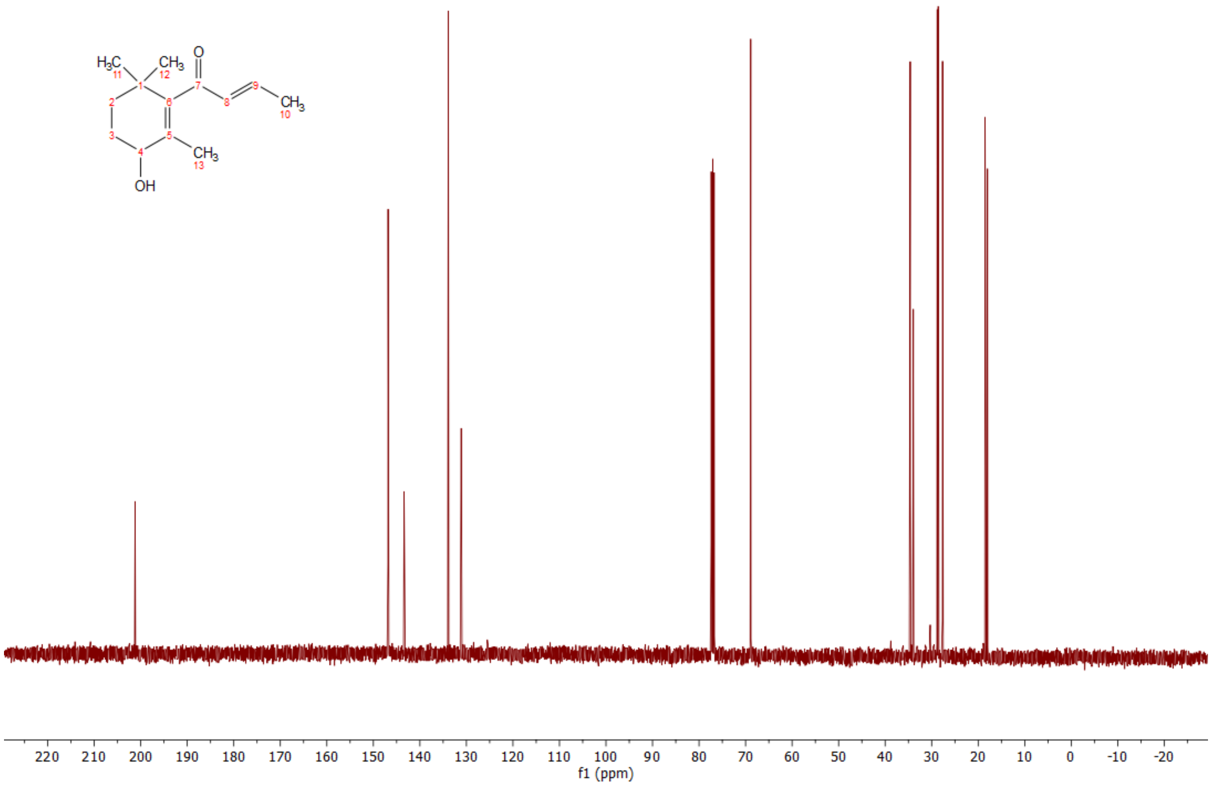

1d – ^{1}H NMR (400 MHz, $CDCl_3$)

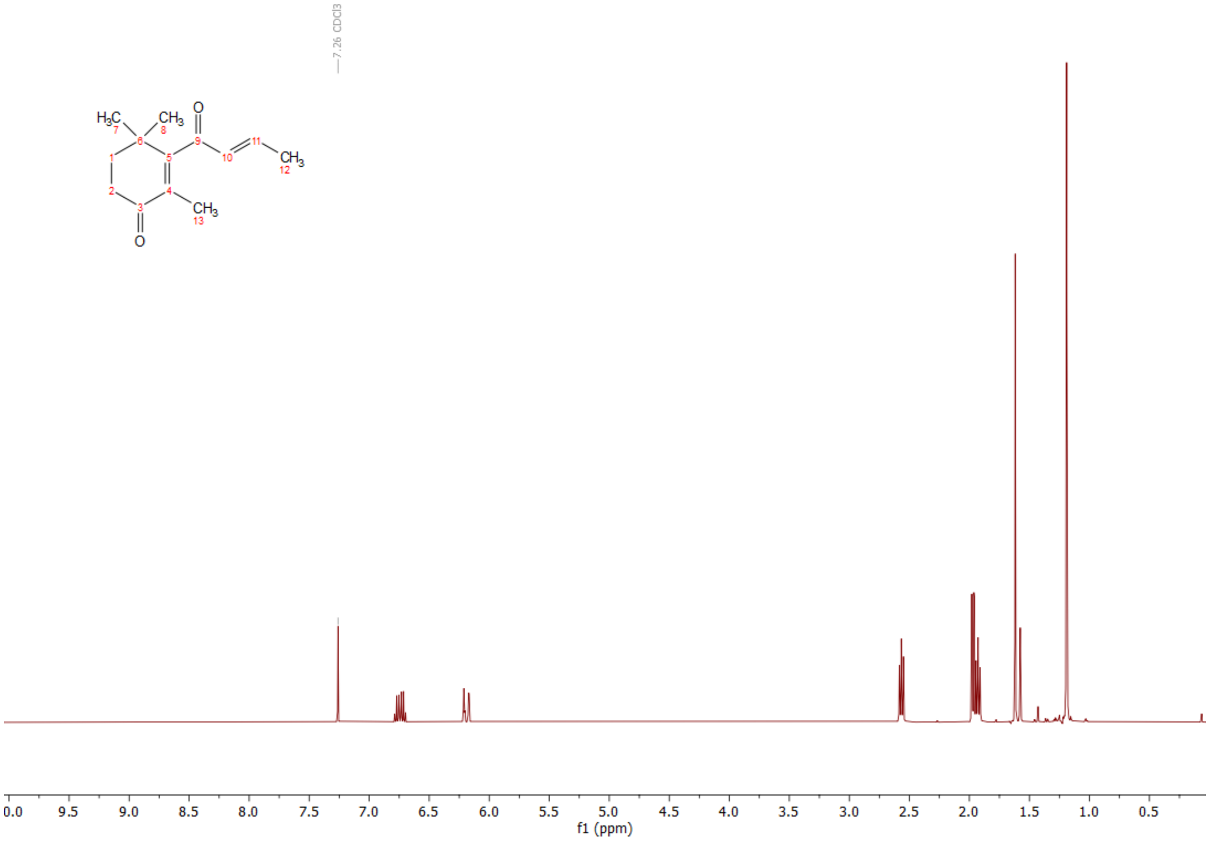

1d – ^{13}C NMR (126 MHz, $CDCl_3$)

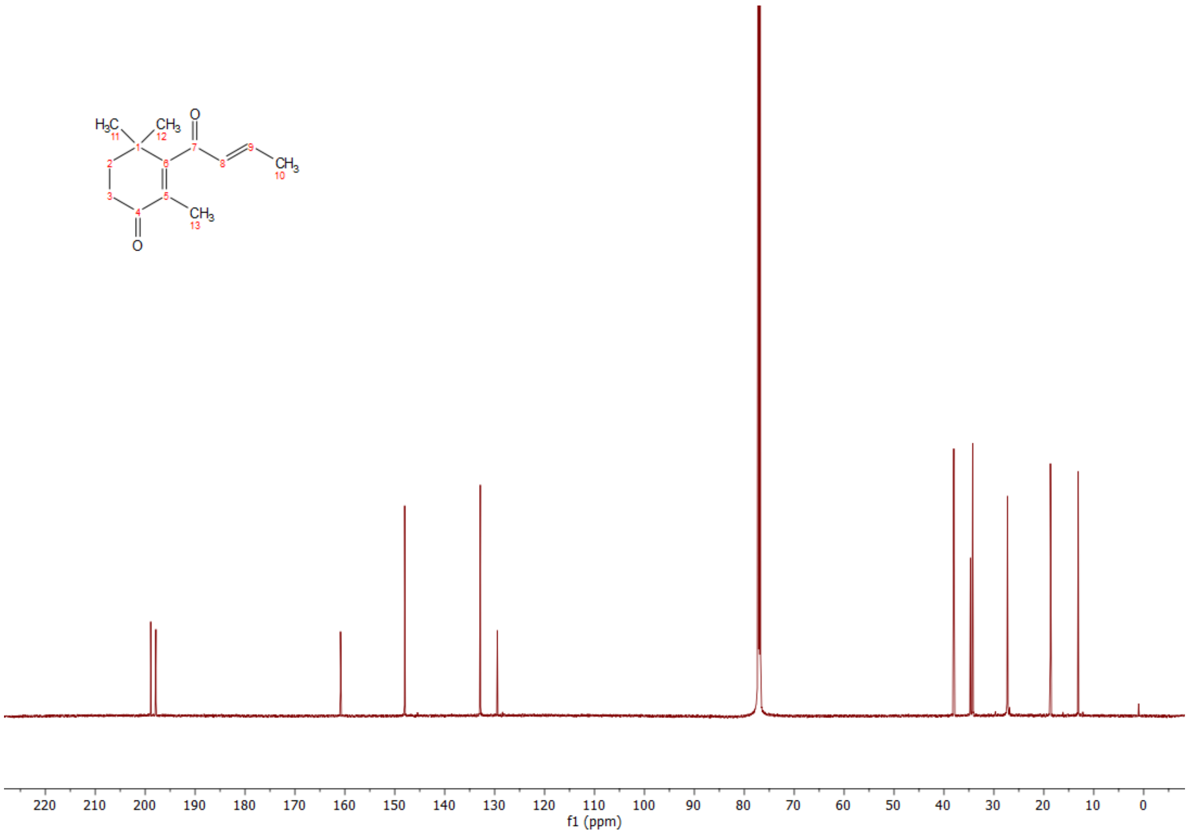

1e – ^{1}H NMR (400 MHz, $CDCl_3$)

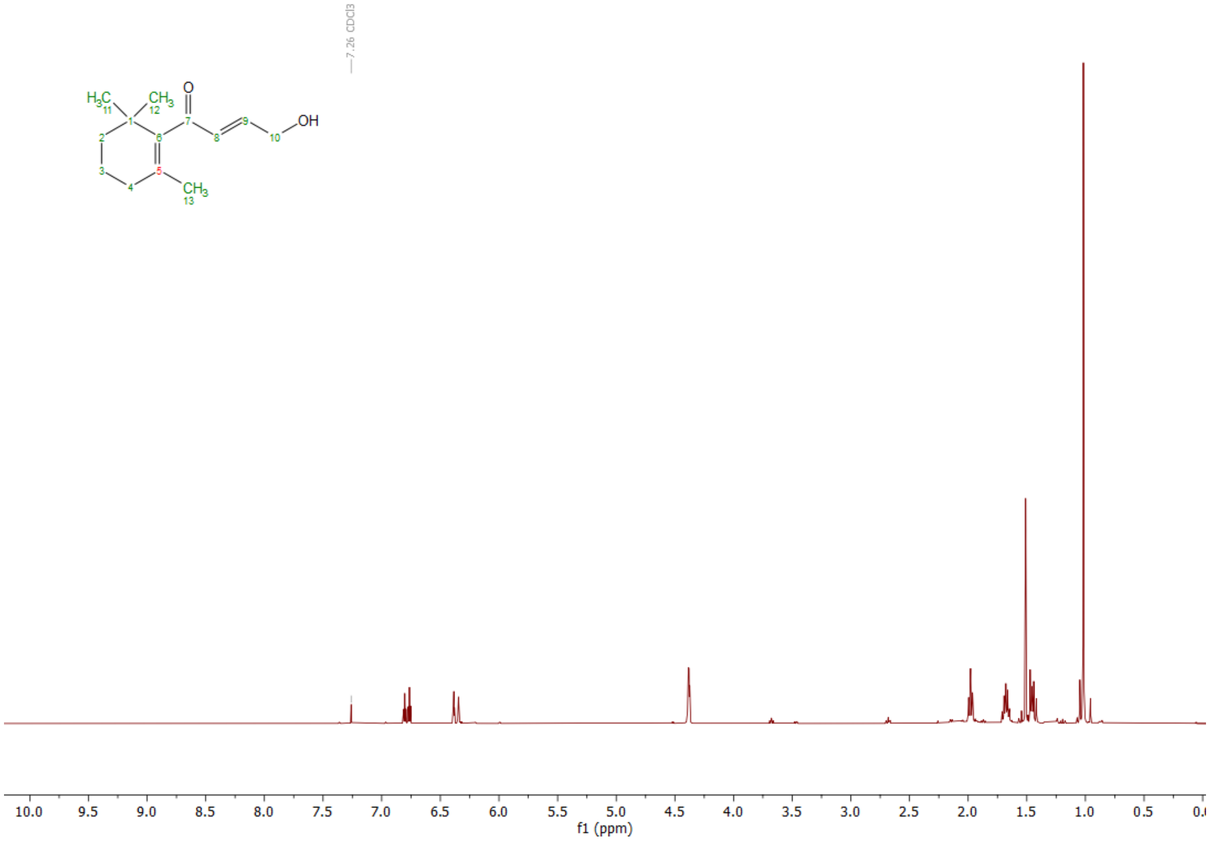

1e – ^{13}C NMR (101 MHz, $CDCl_3$)

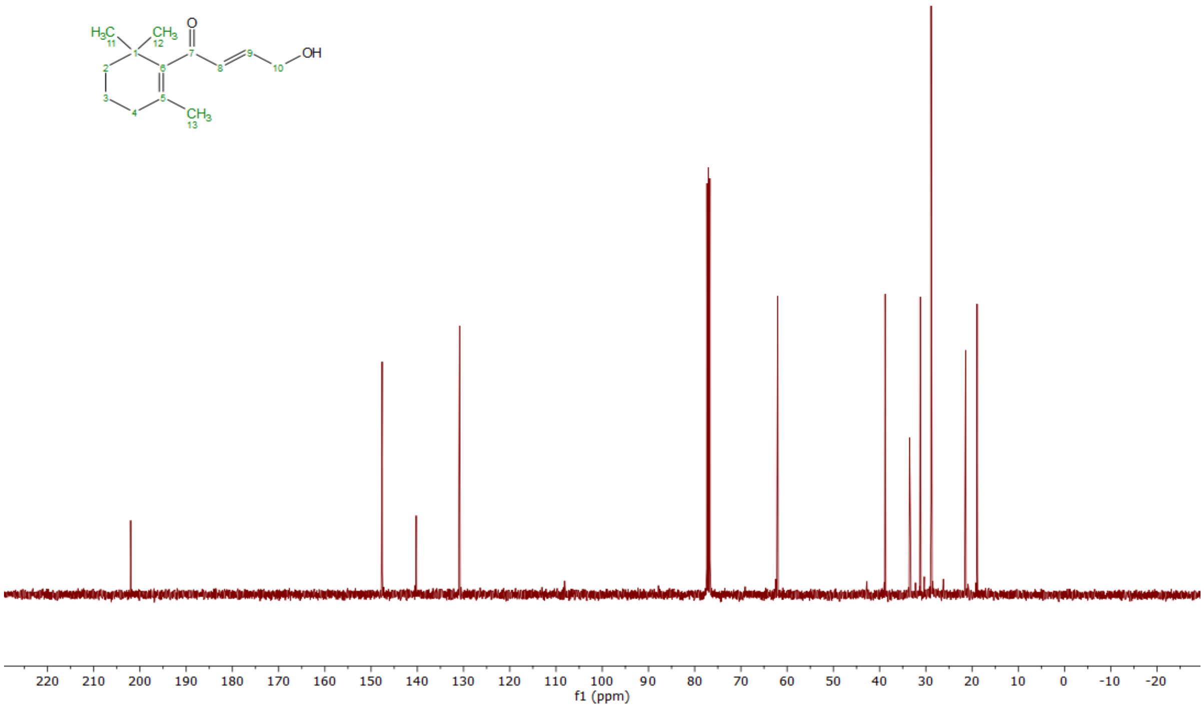

2 – ^{1}H NMR (400 MHz, $CDCl_3$)

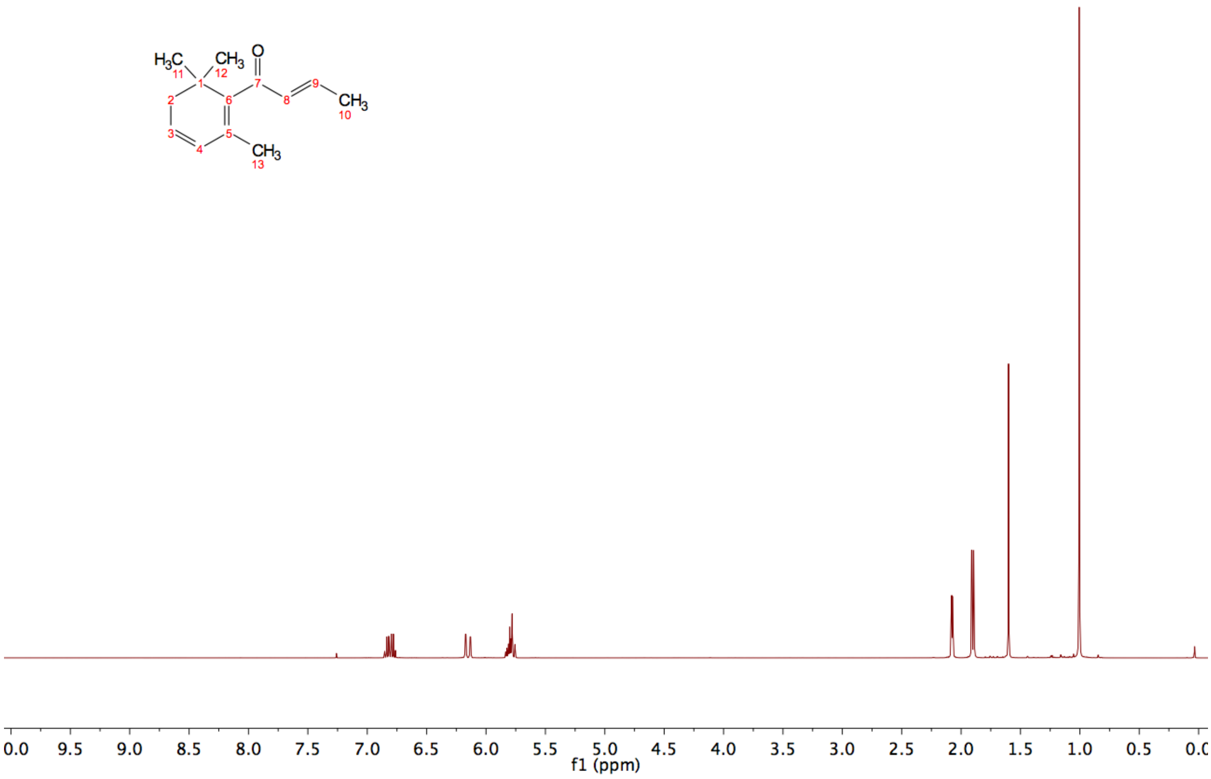

2 – ^{13}C NMR (101 MHz, $CDCl_3$)

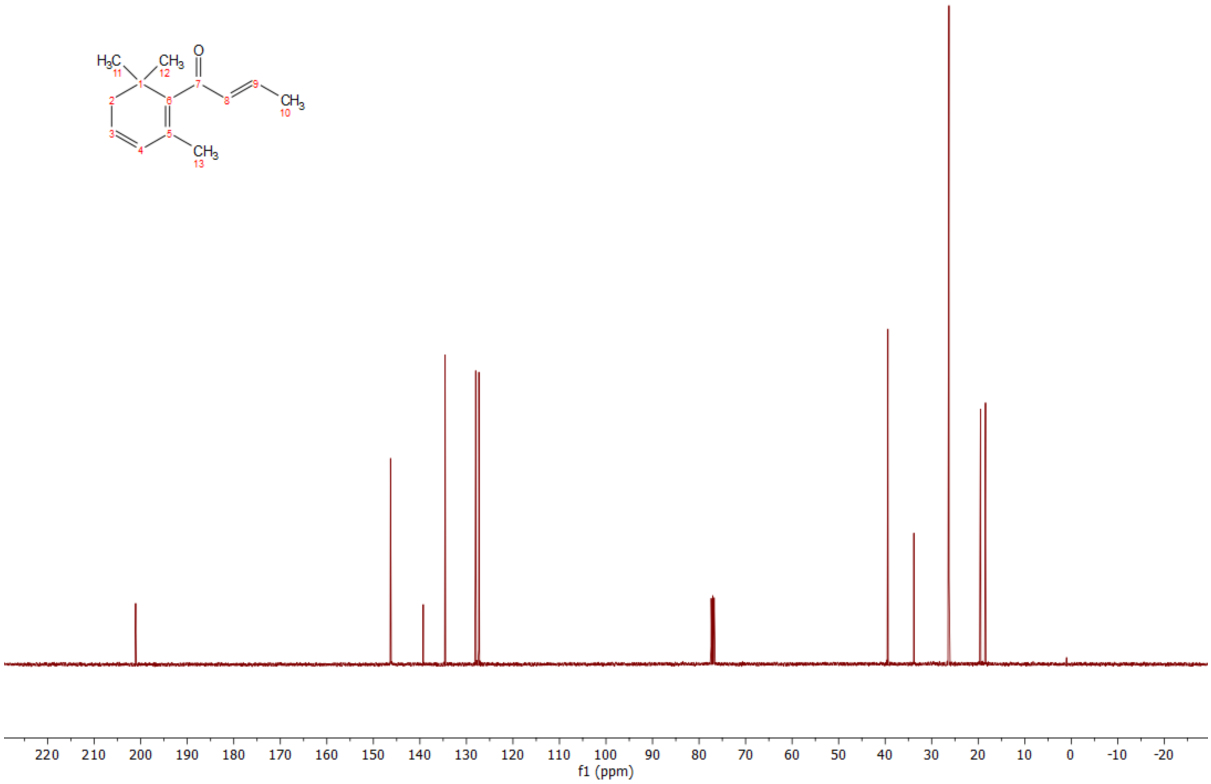

E1 – ^{1}H NMR (400 MHz, $CDCl_3$)

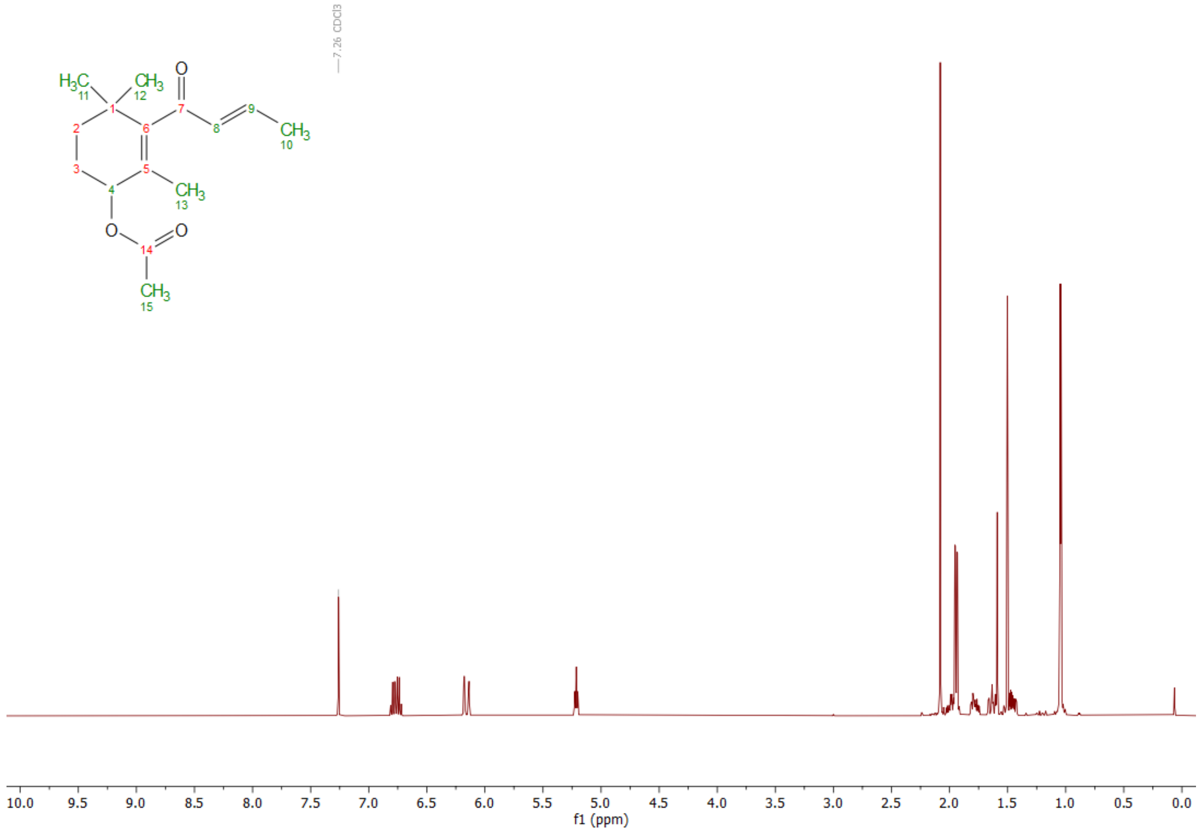

E1 – ^{13}C NMR (101 MHz, $CDCl_3$)

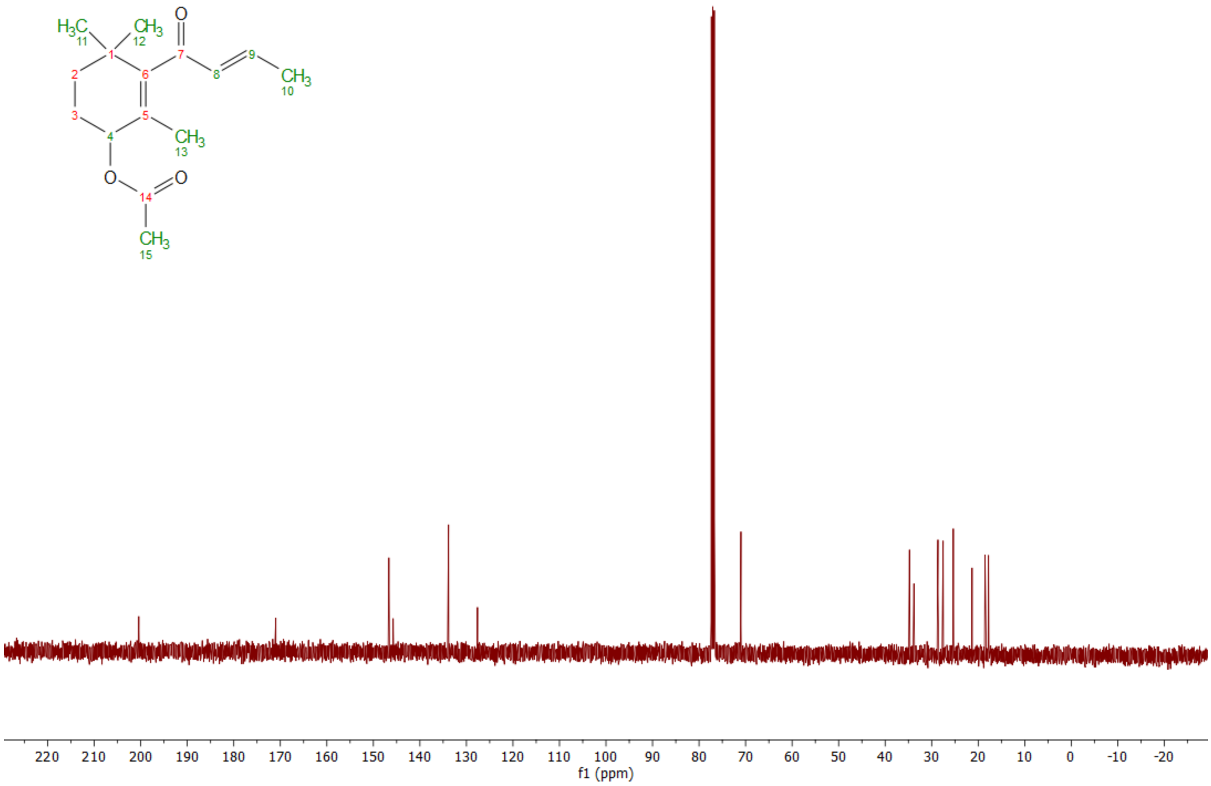

E2 – ^{1}H NMR (400 MHz, $CDCl_3$)

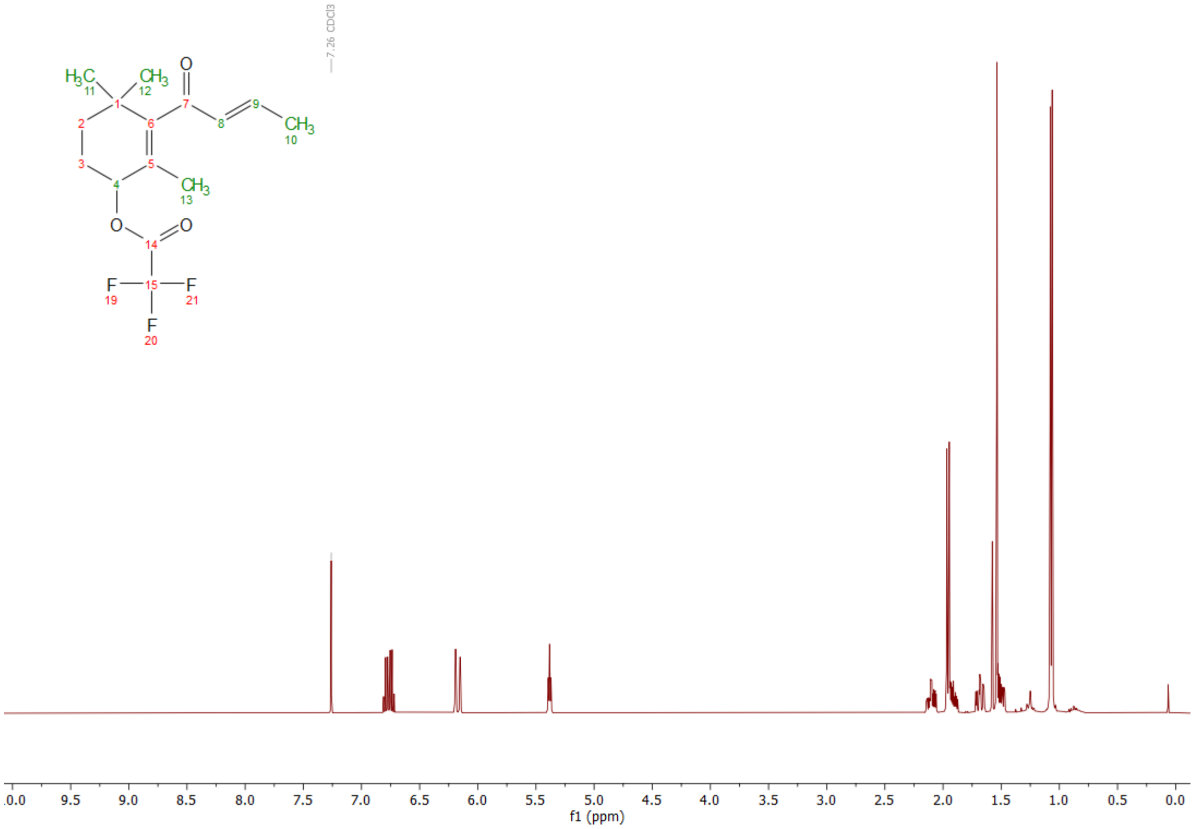

E2 – ^{13}C NMR (101 MHz, $CDCl_3$)

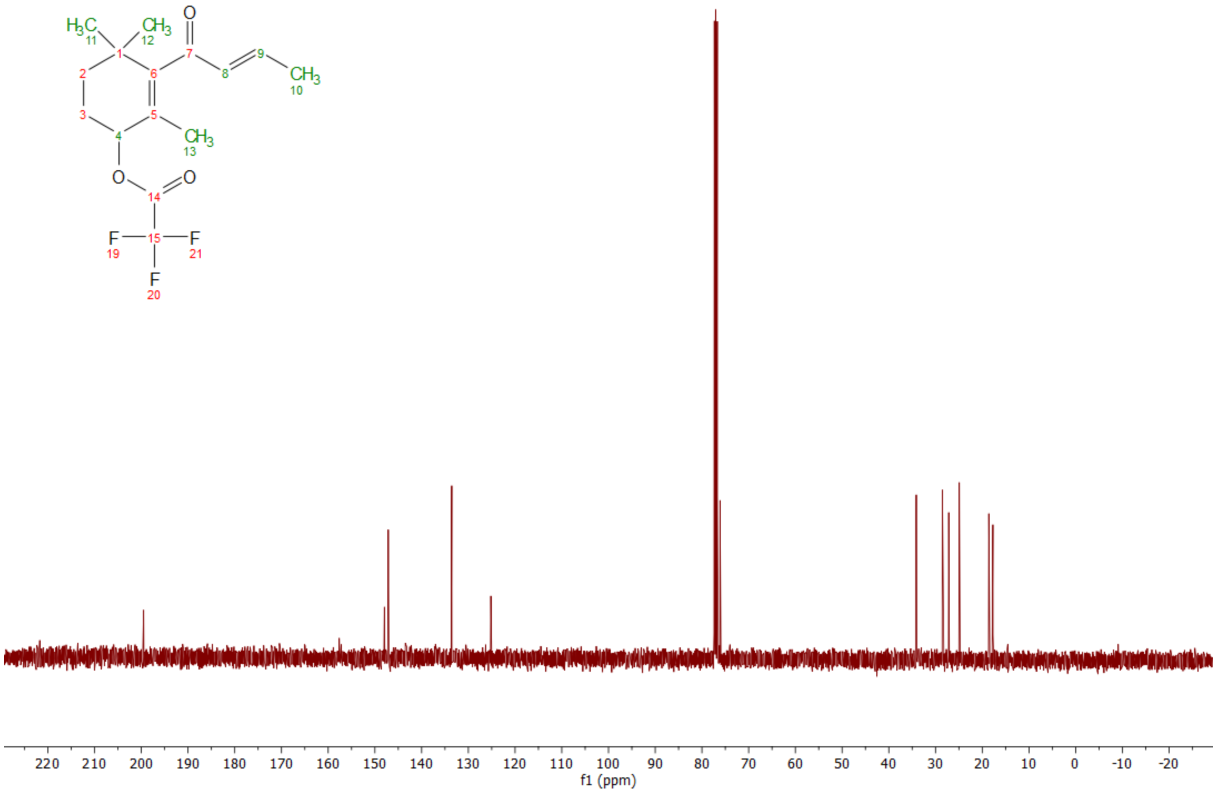

3a – ^{1}H NMR (400 MHz, $CDCl_3$)

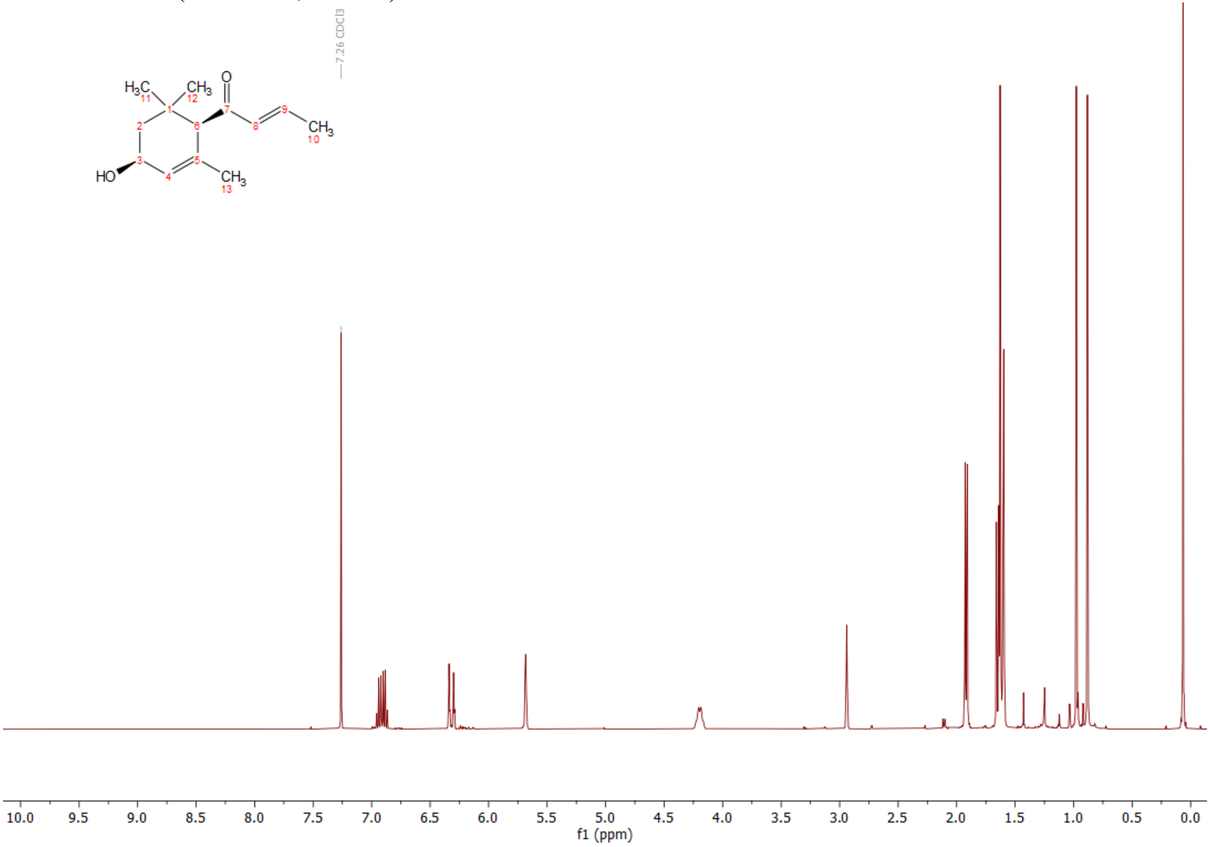

3a – ^{13}C NMR (126 MHz, $CDCl_3$)

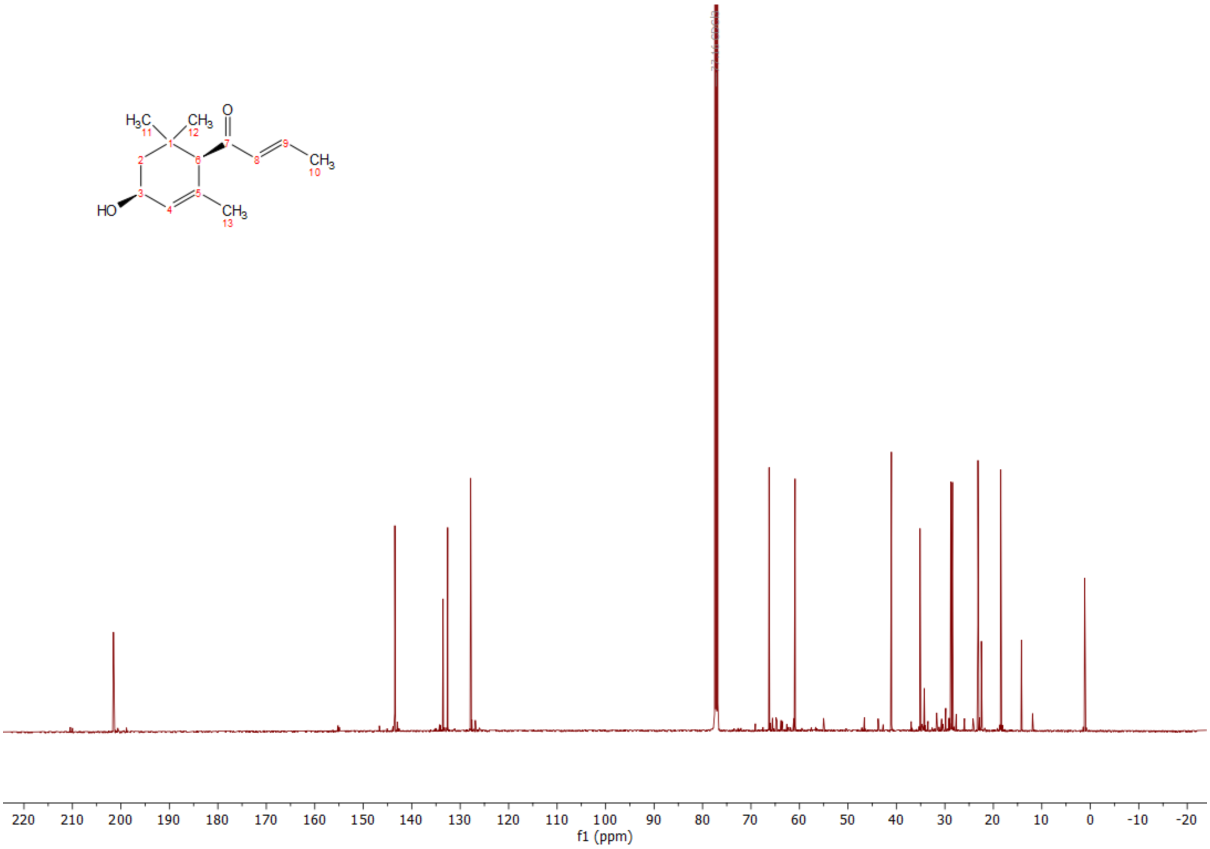

3b – ^{1}H NMR (500 MHz, $CDCl_3$)

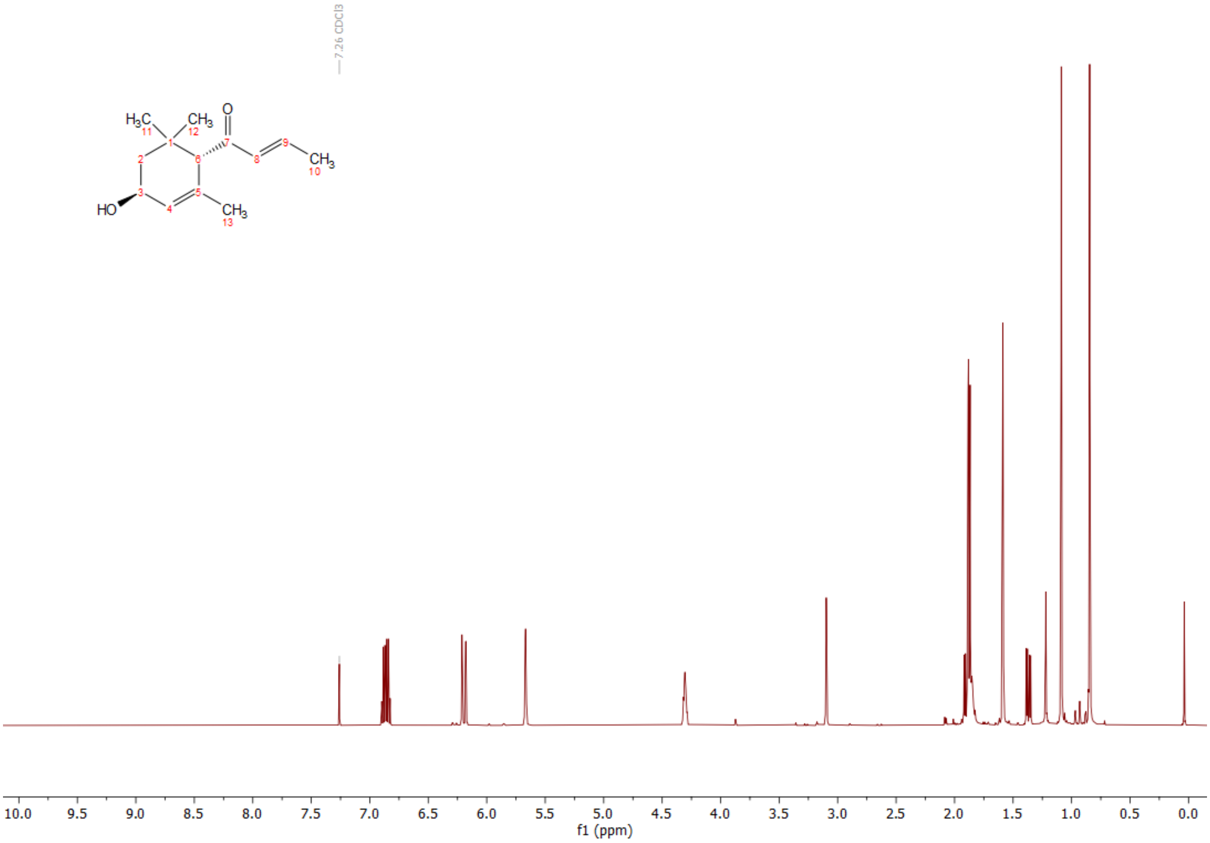

3b – ^{13}C NMR (126 MHz, $CDCl_3$)

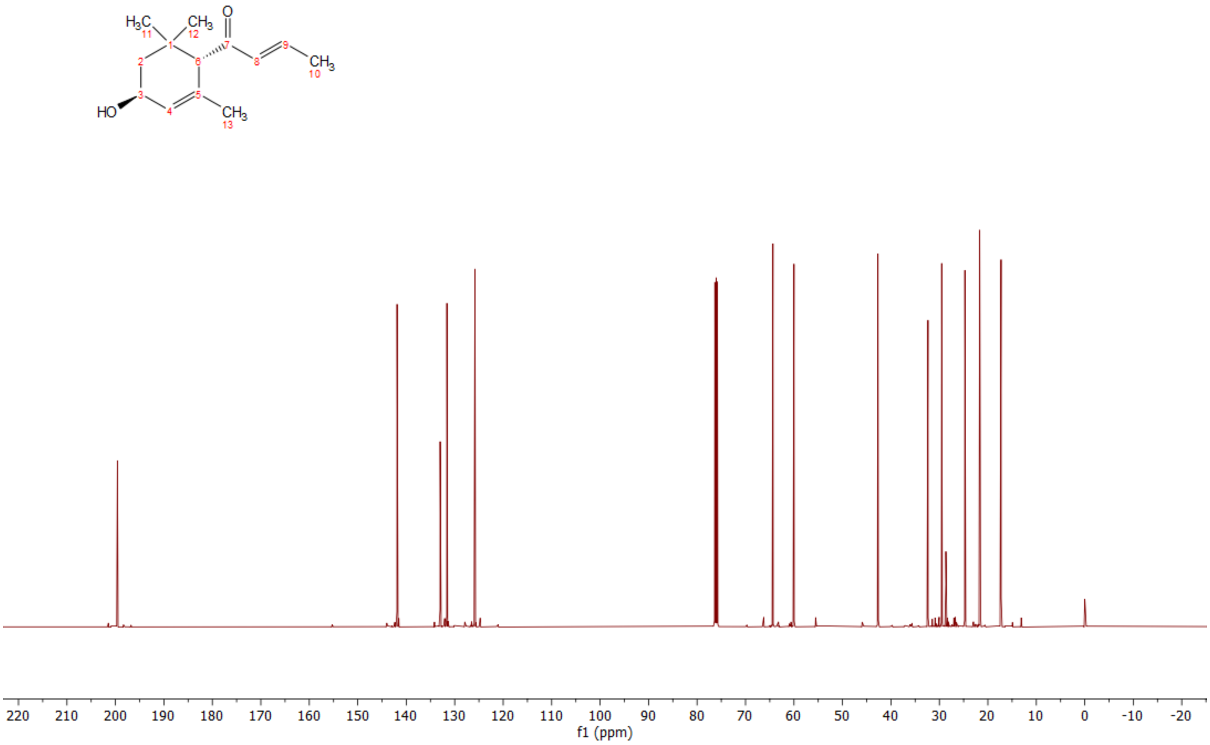

3c – ^{1}H NMR (400 MHz, $CDCl_3$)

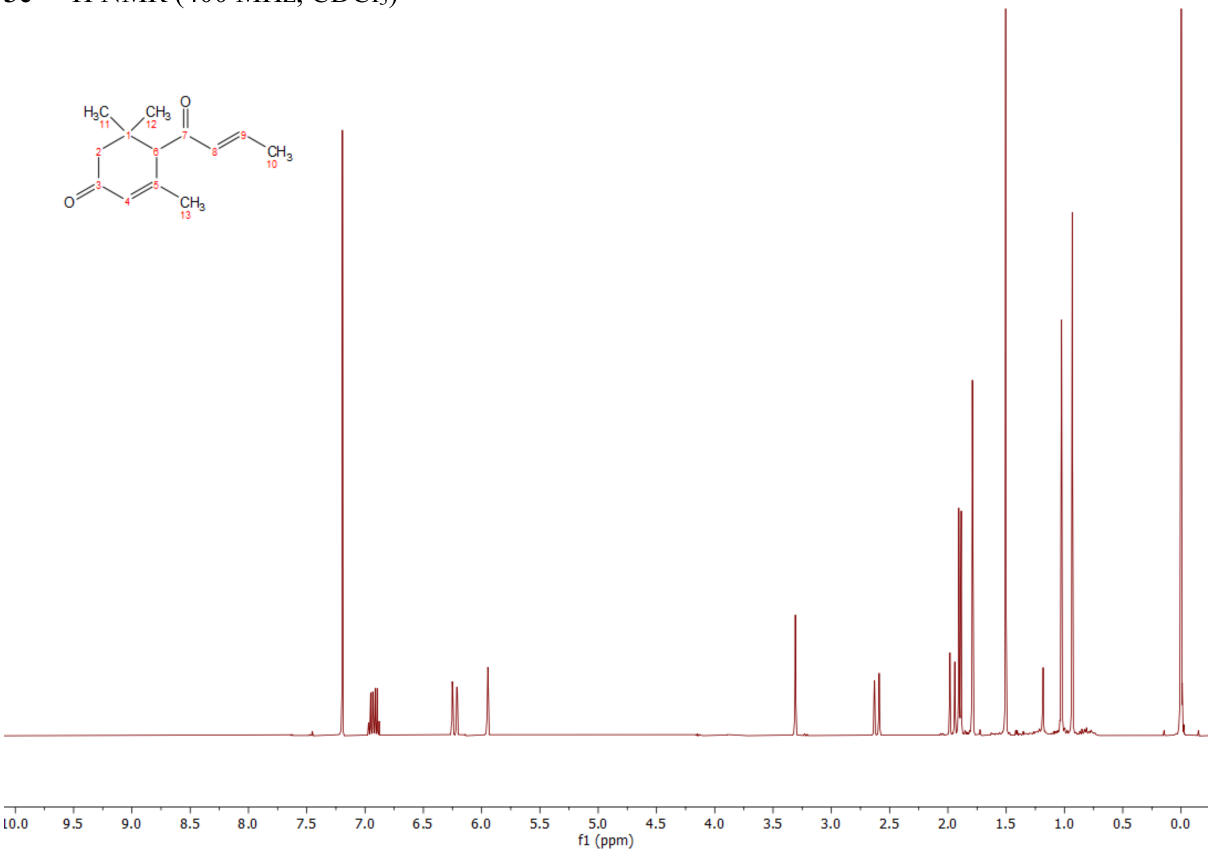

3c – ^{13}C NMR (101 MHz, $CDCl_3$)

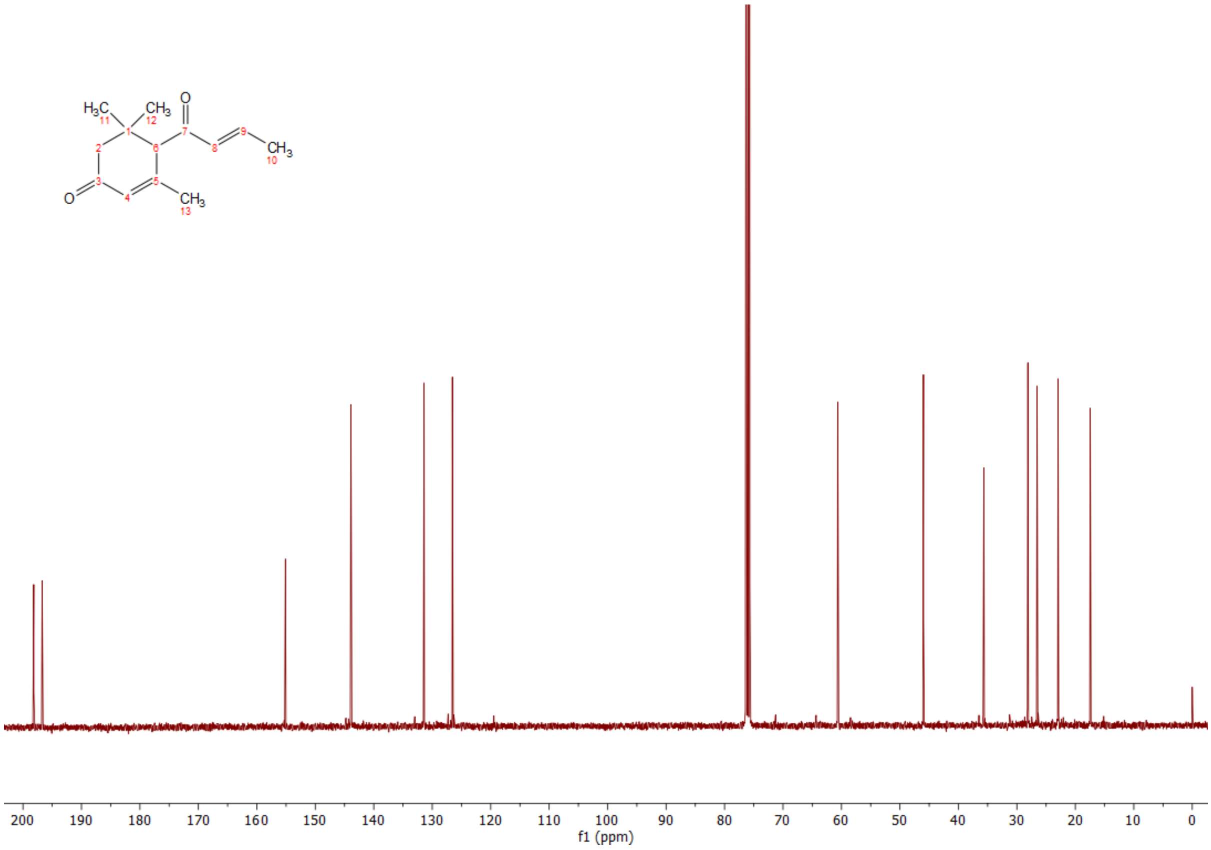

3d – ^{1}H NMR (400 MHz, CD_3OD)

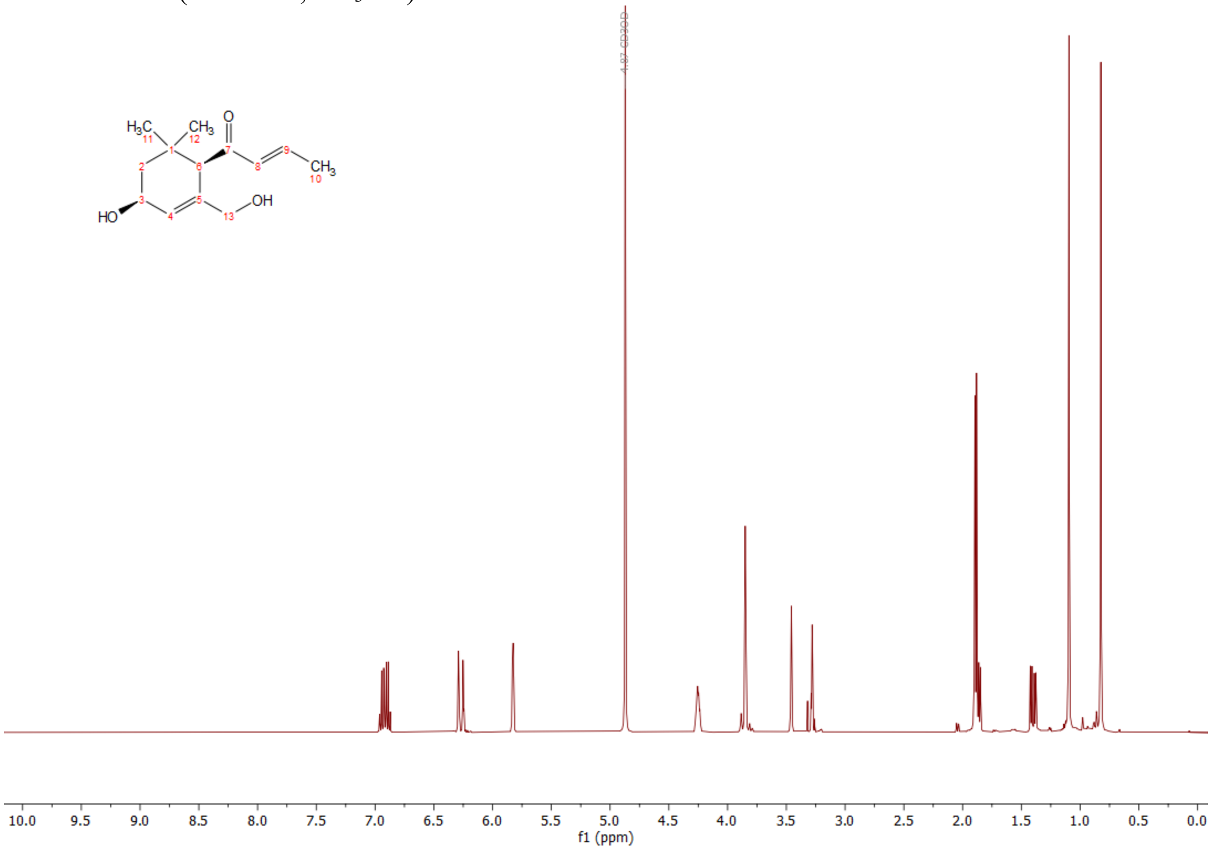

3d – ^{13}C NMR (101 MHz, CD_3OD)

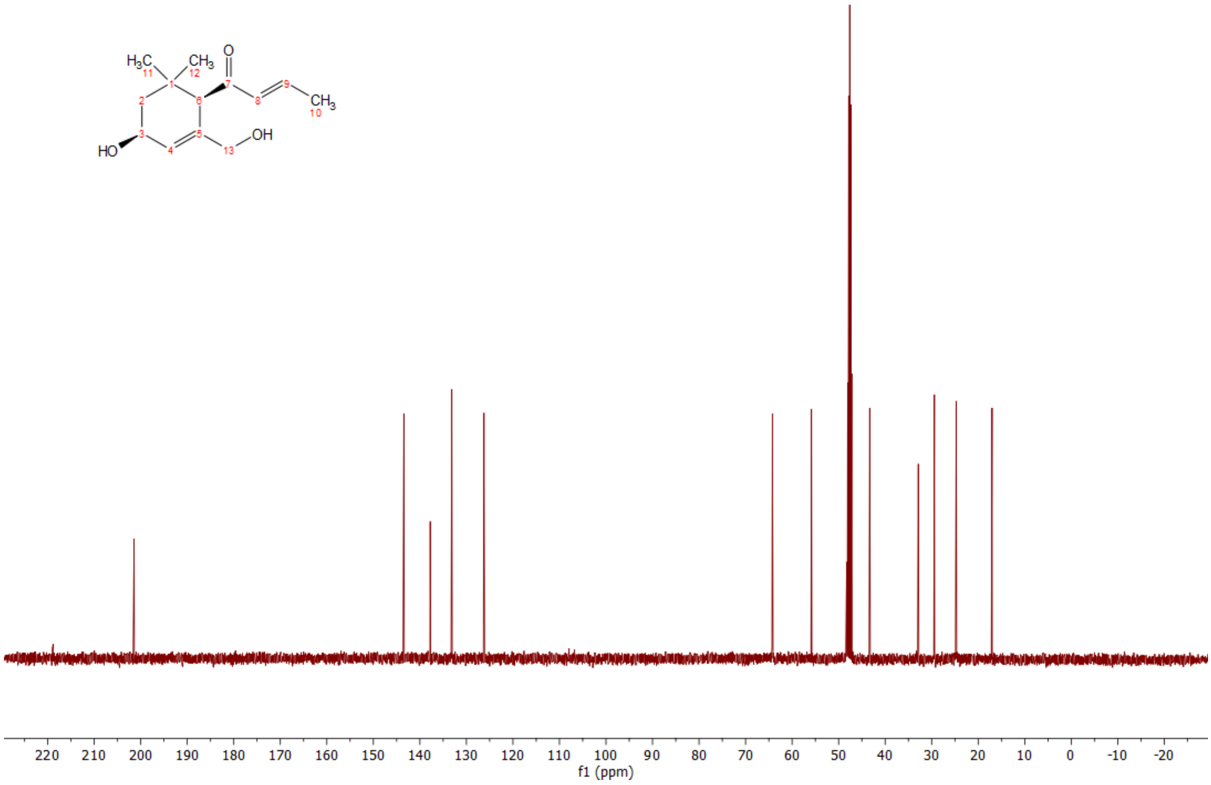

4 – ^{1}H NMR (500 MHz, $CDCl_3$)

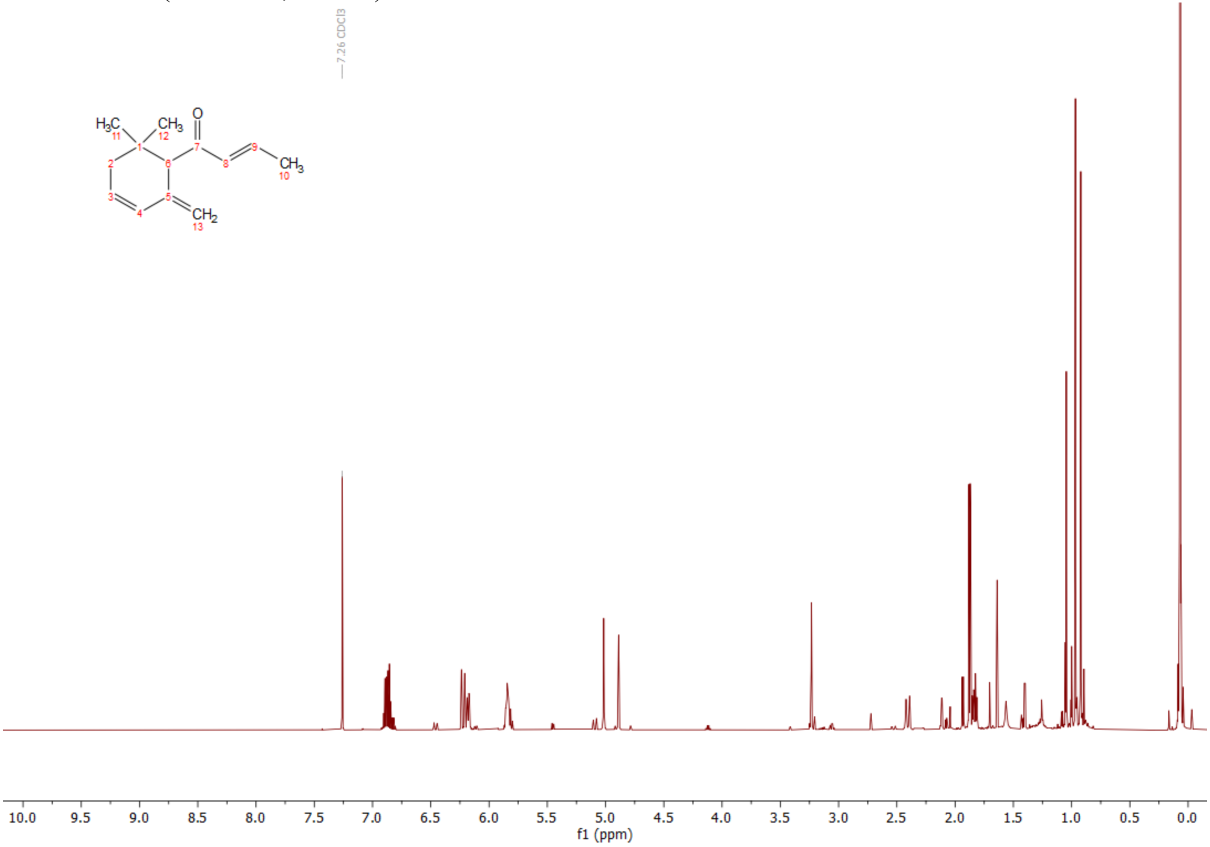

4 – ^{13}C NMR (126 MHz, $CDCl_3$)

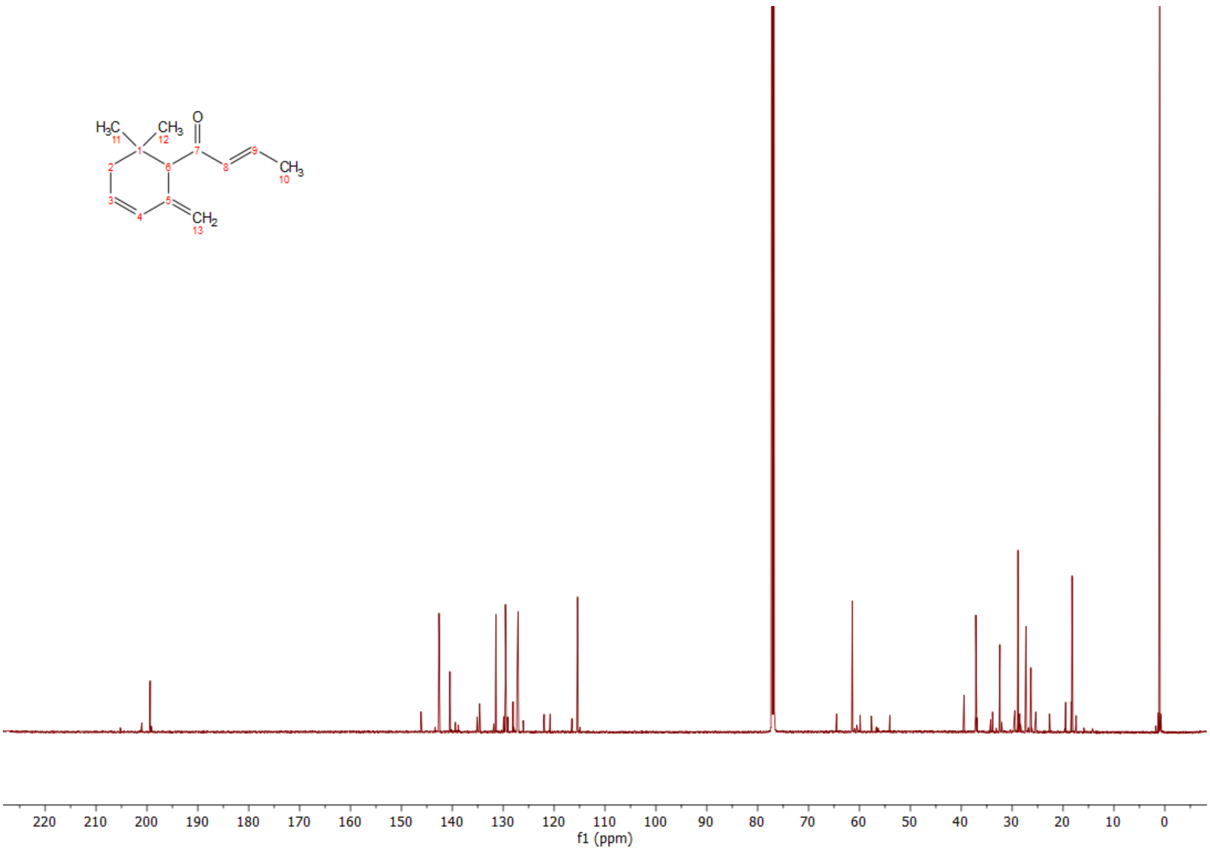

5a – ^{1}H NMR (400 MHz, $CDCl_3$)

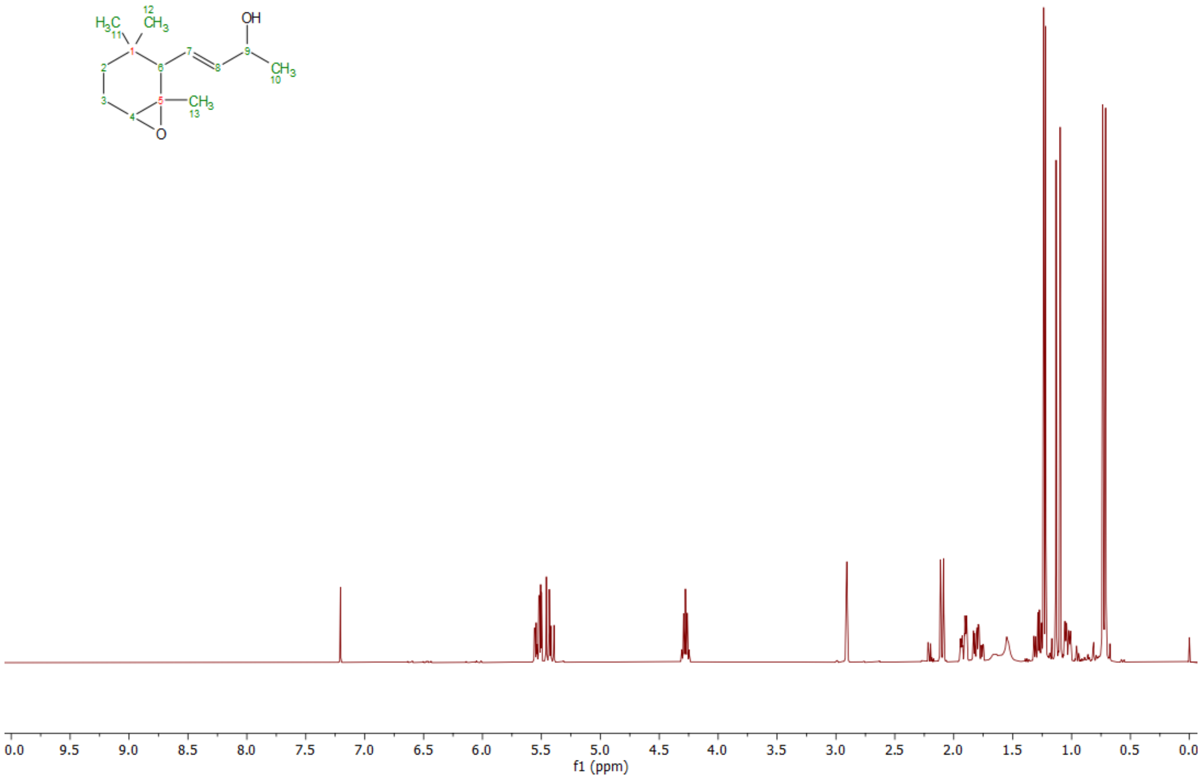

5a – ^{13}C NMR (101 MHz, $CDCl_3$)

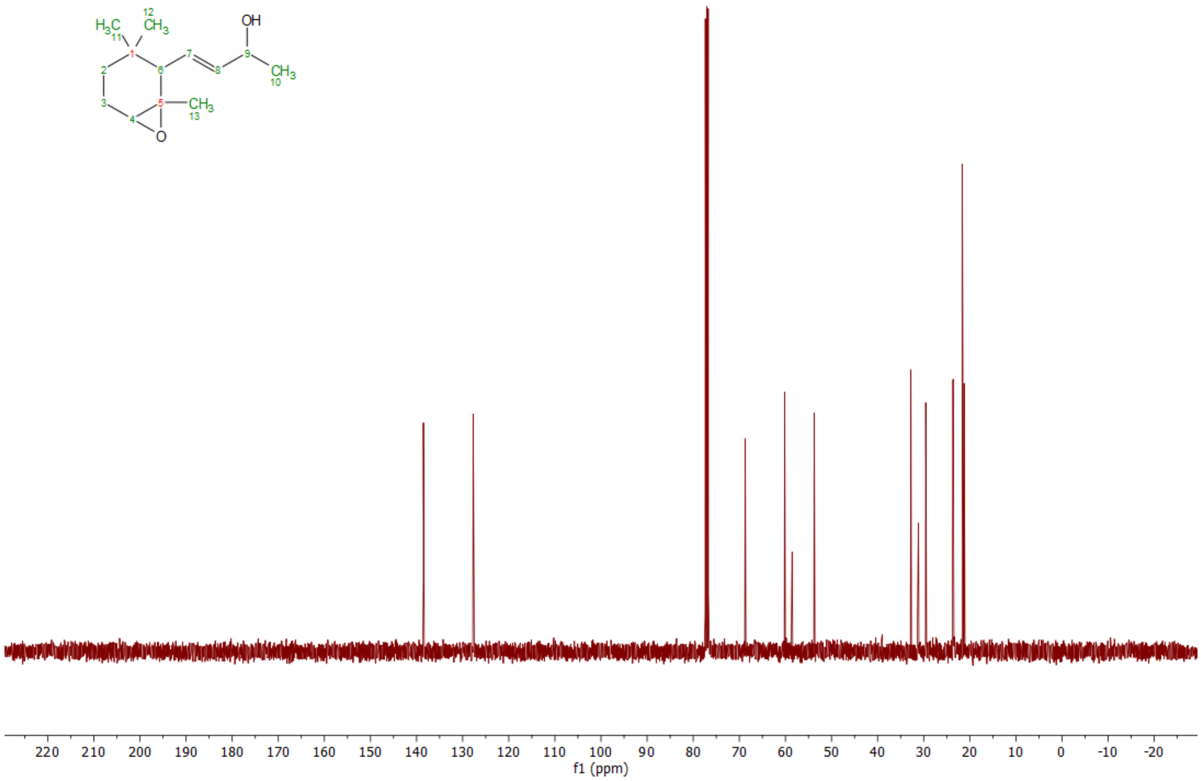

5b – ^{1}H NMR (400 MHz, $CDCl_3$)

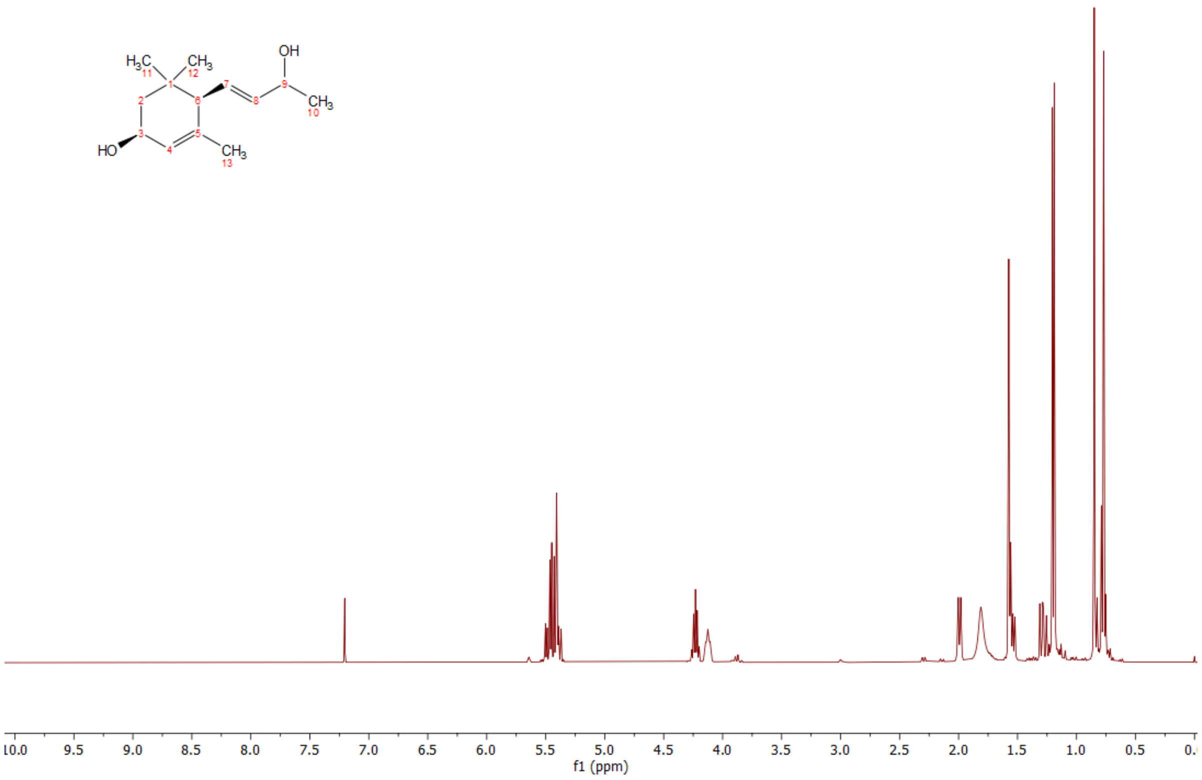

5b – ^{13}C NMR (101 MHz, $CDCl_3$)

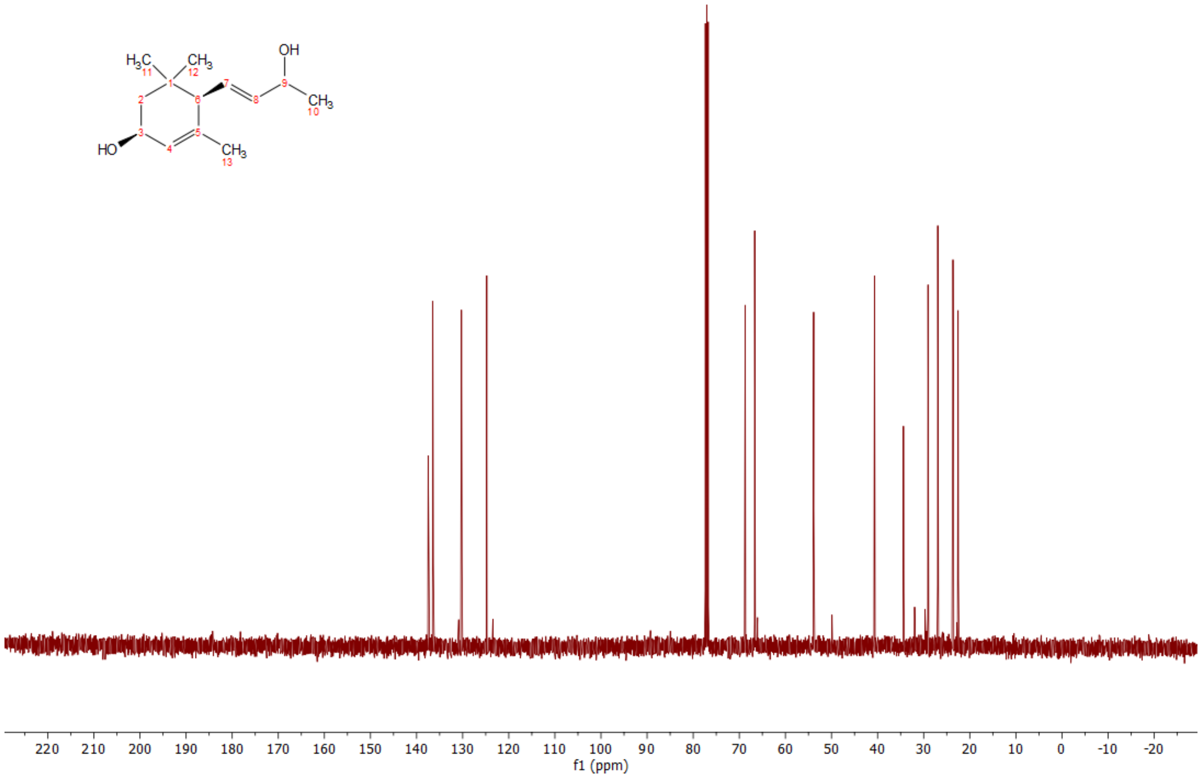

5c – ^{1}H NMR (400 MHz, $CDCl_3$)

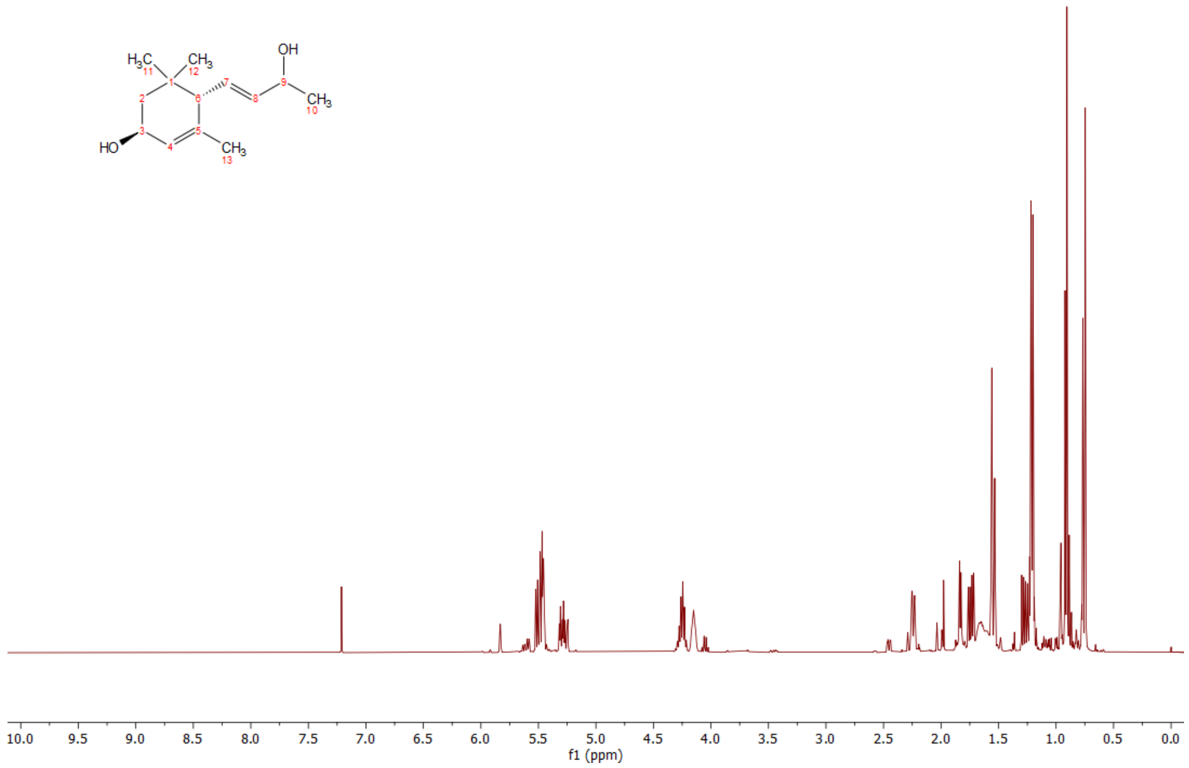

5c – ^{13}C NMR (101 MHz, $CDCl_3$)

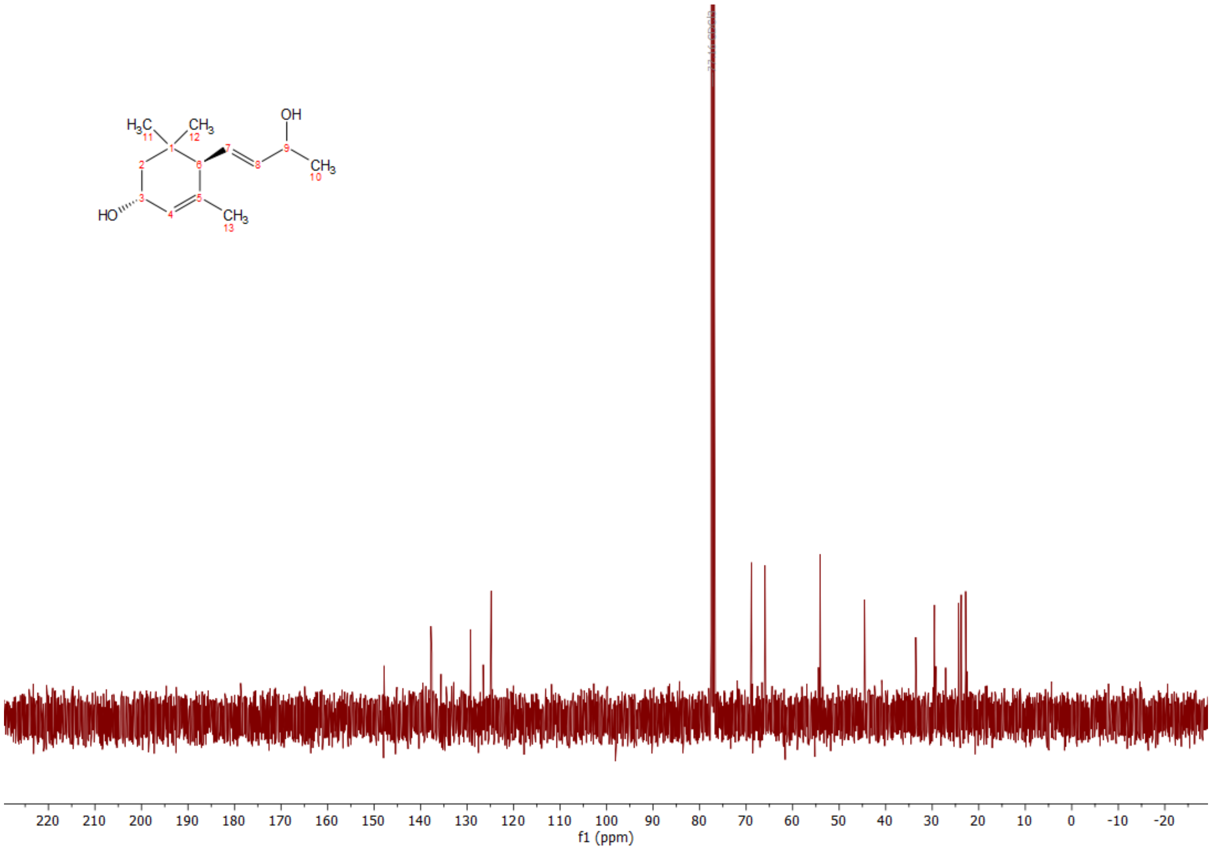

5d – ^{1}H NMR (400 MHz, CD_3OD)

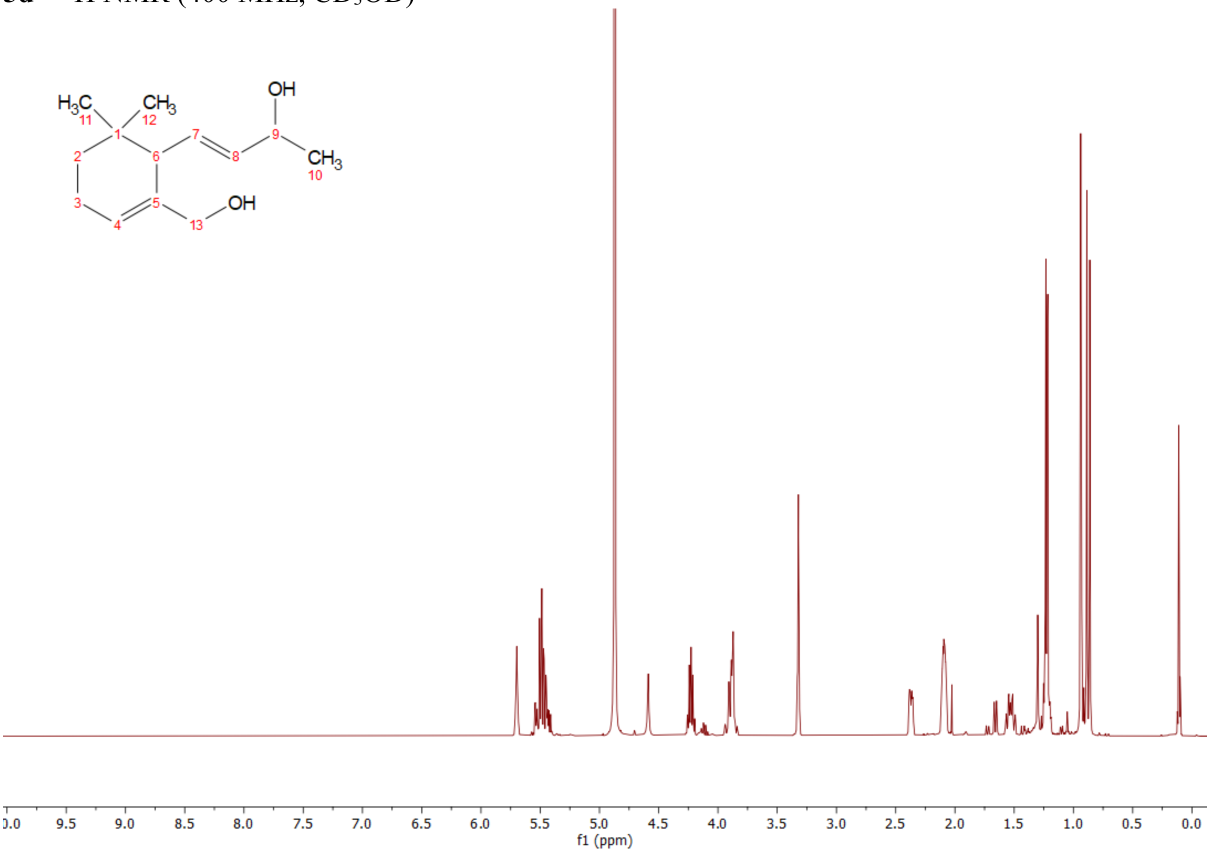

5d – ^{13}C NMR (101 MHz, CD_3OD)

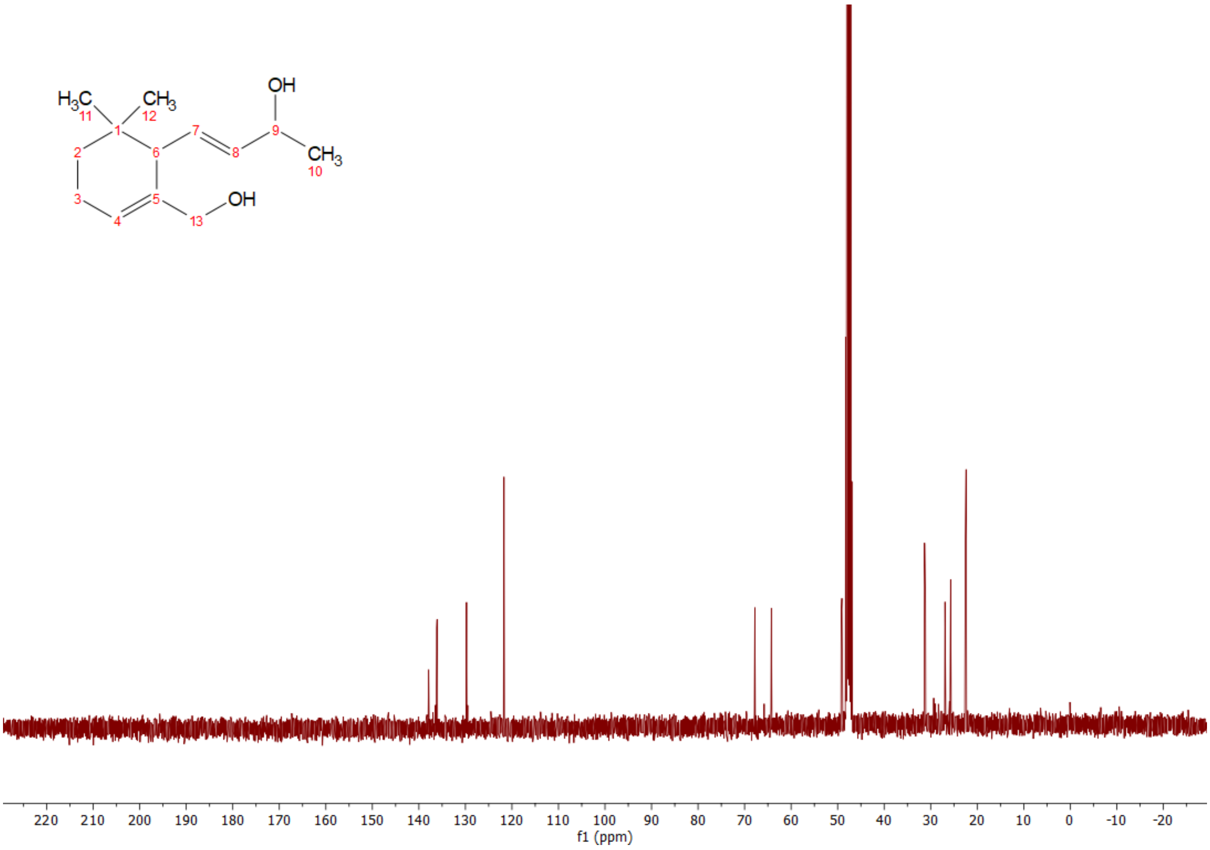

5e – ^{1}H NMR (500 MHz, $CDCl_3$)

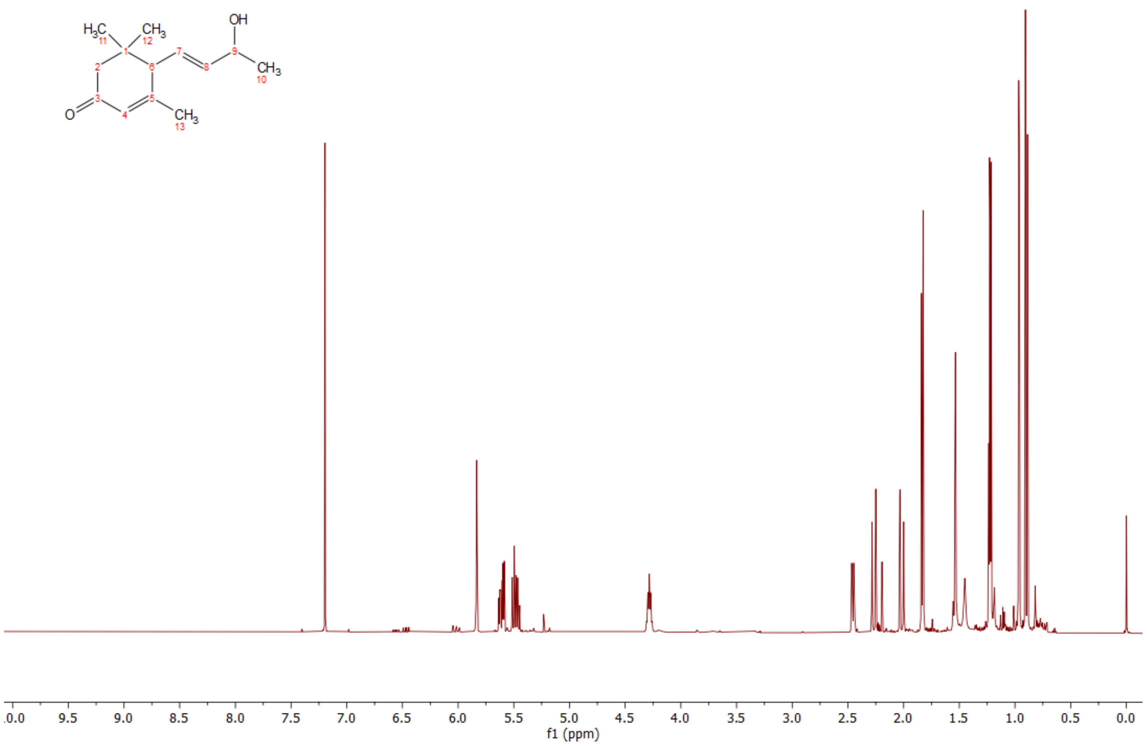

5e – ^{13}C NMR (126 MHz, $CDCl_3$)

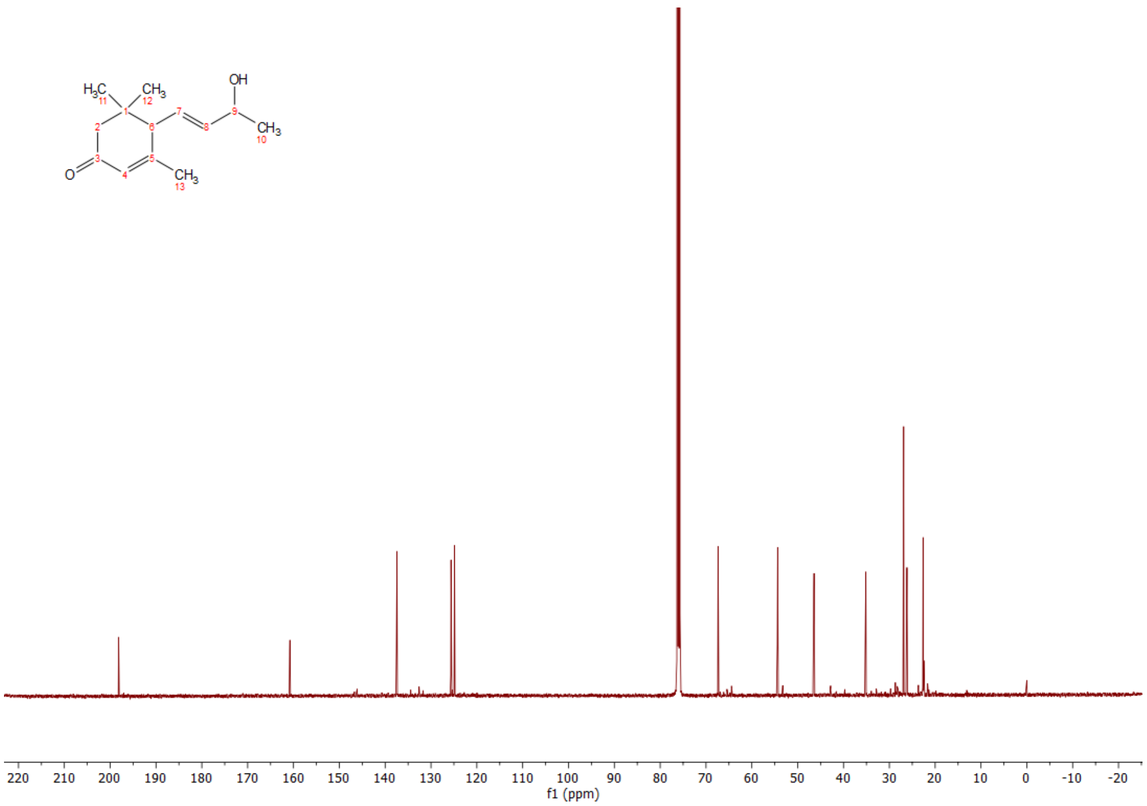

5f – ^{1}H NMR (400 MHz, $CDCl_3$)

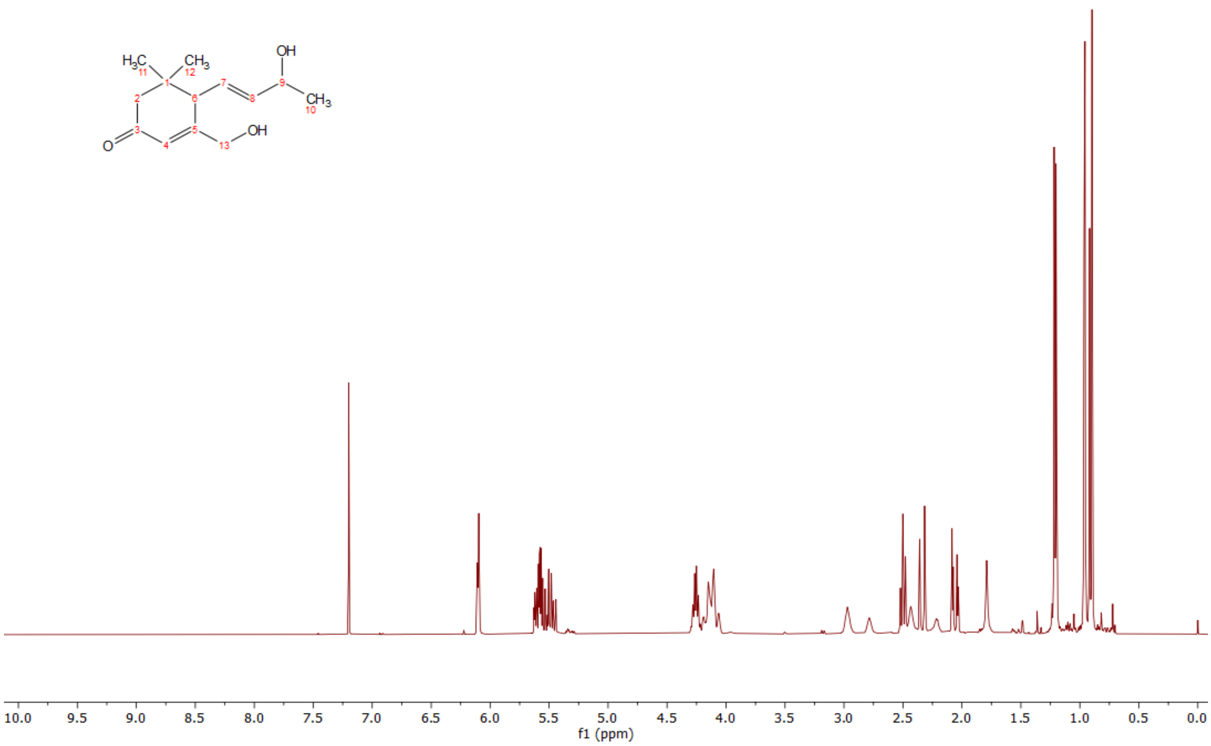

5f – ^{13}C NMR (101 MHz, $CDCl_3$)

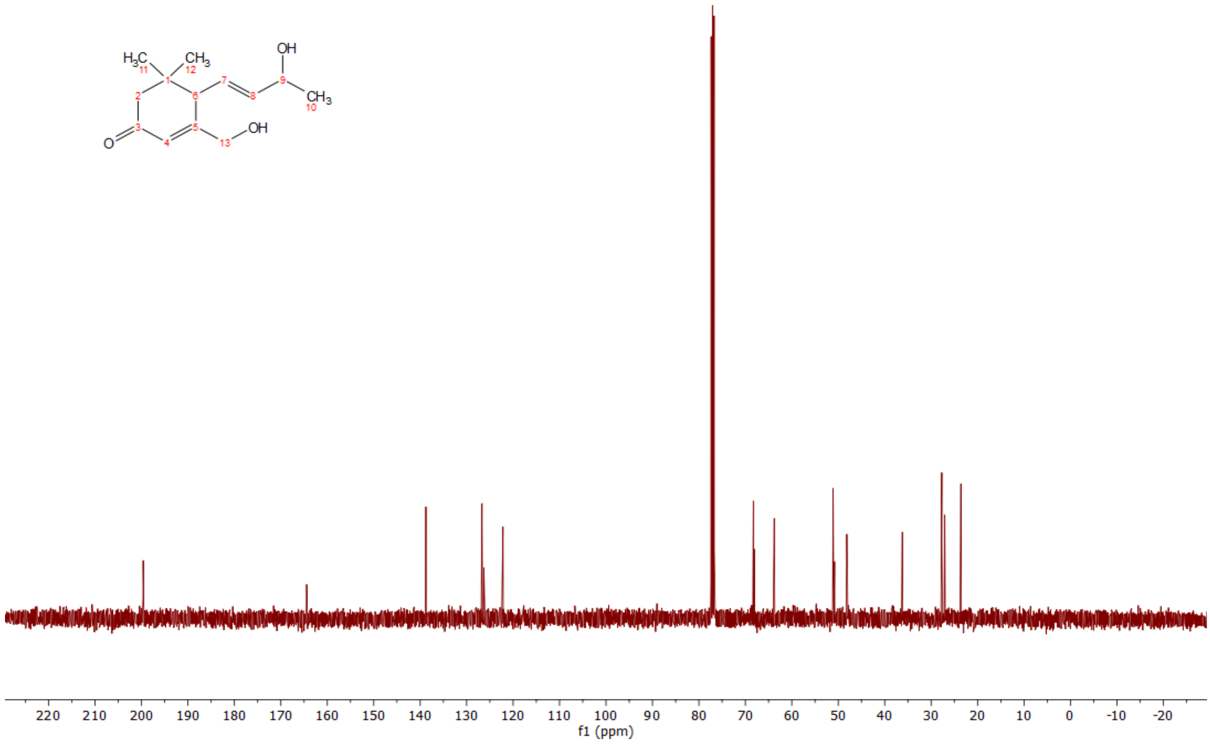

6a – ^{1}H NMR (500 MHz, $CDCl_3$)

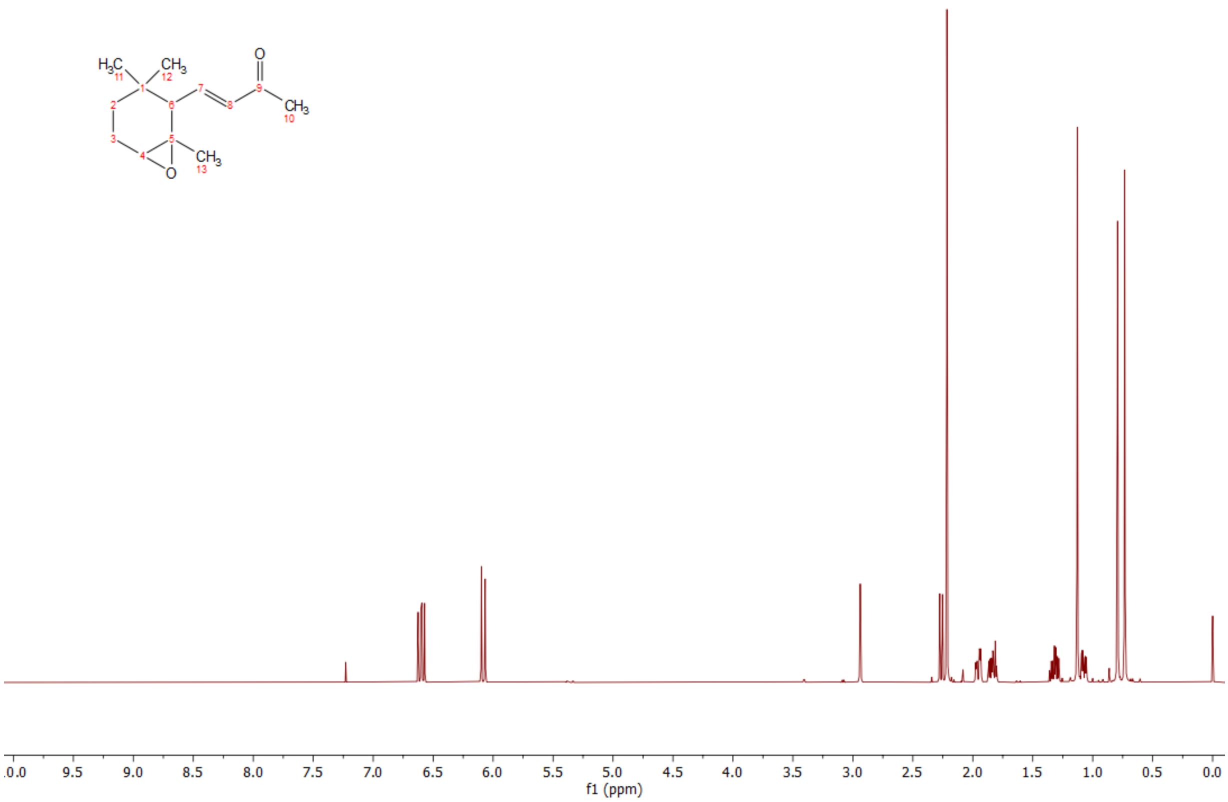

6a – ^{13}C NMR (126 MHz, $CDCl_3$)

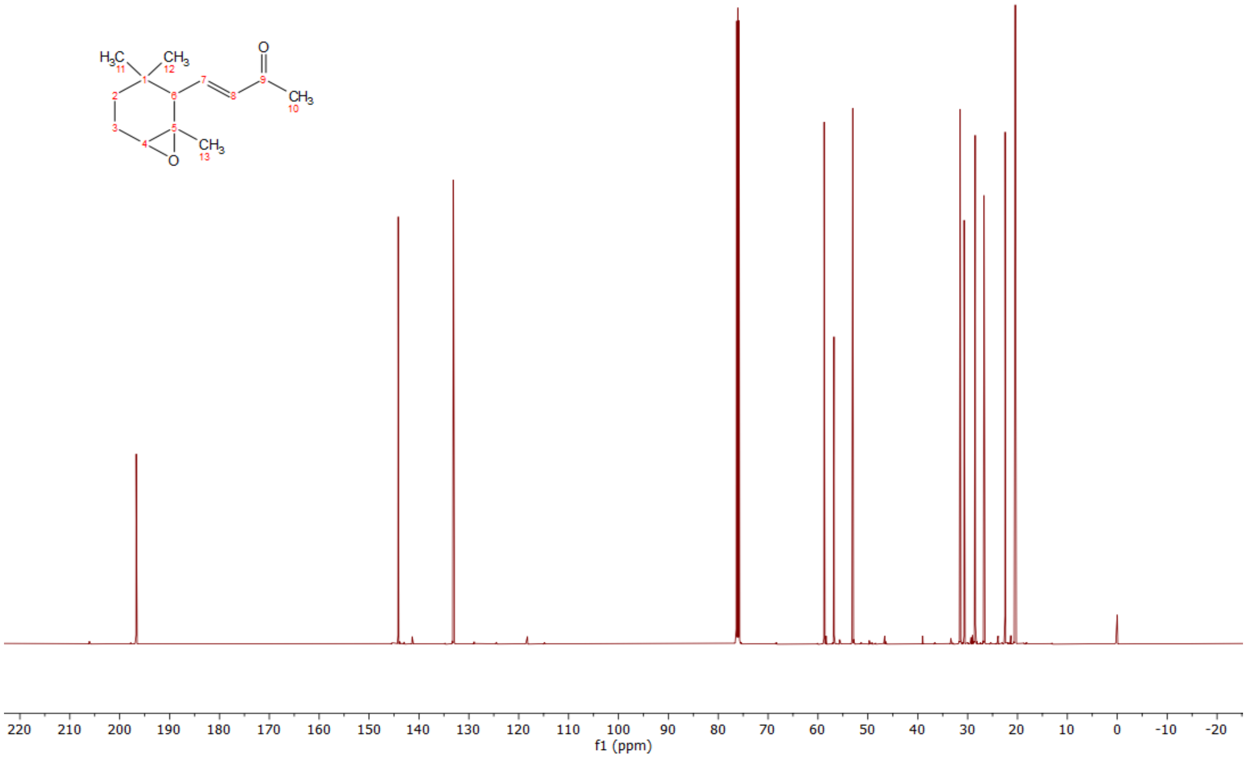

6b – ^{1}H NMR (500 MHz, $CDCl_3$)

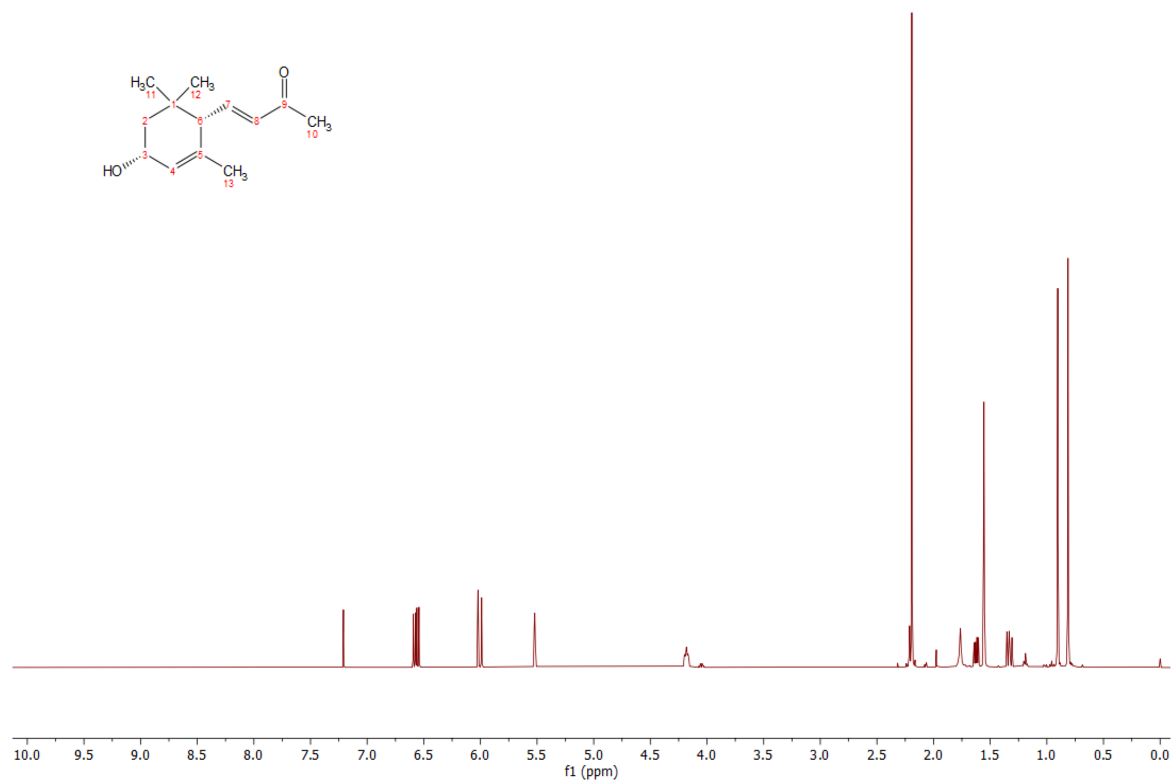

6b – ^{13}C NMR (126 MHz, $CDCl_3$)

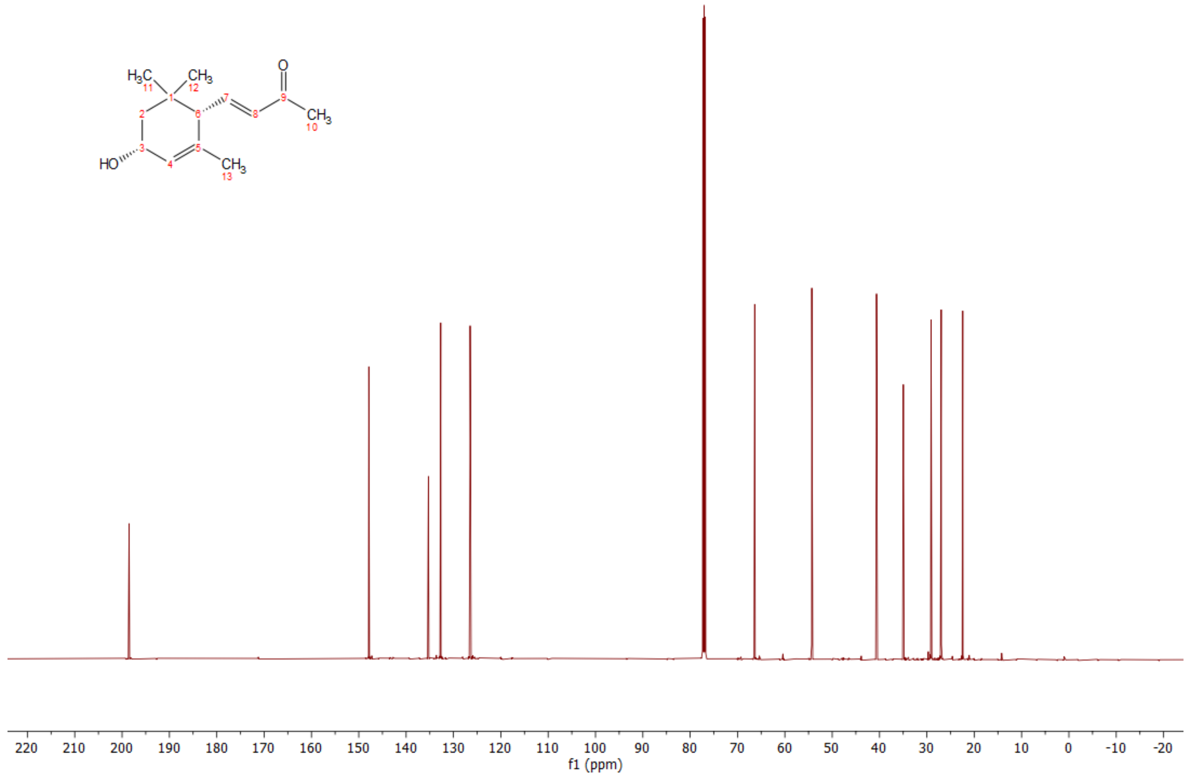

6c – ^{1}H NMR (500 MHz, $CDCl_3$)

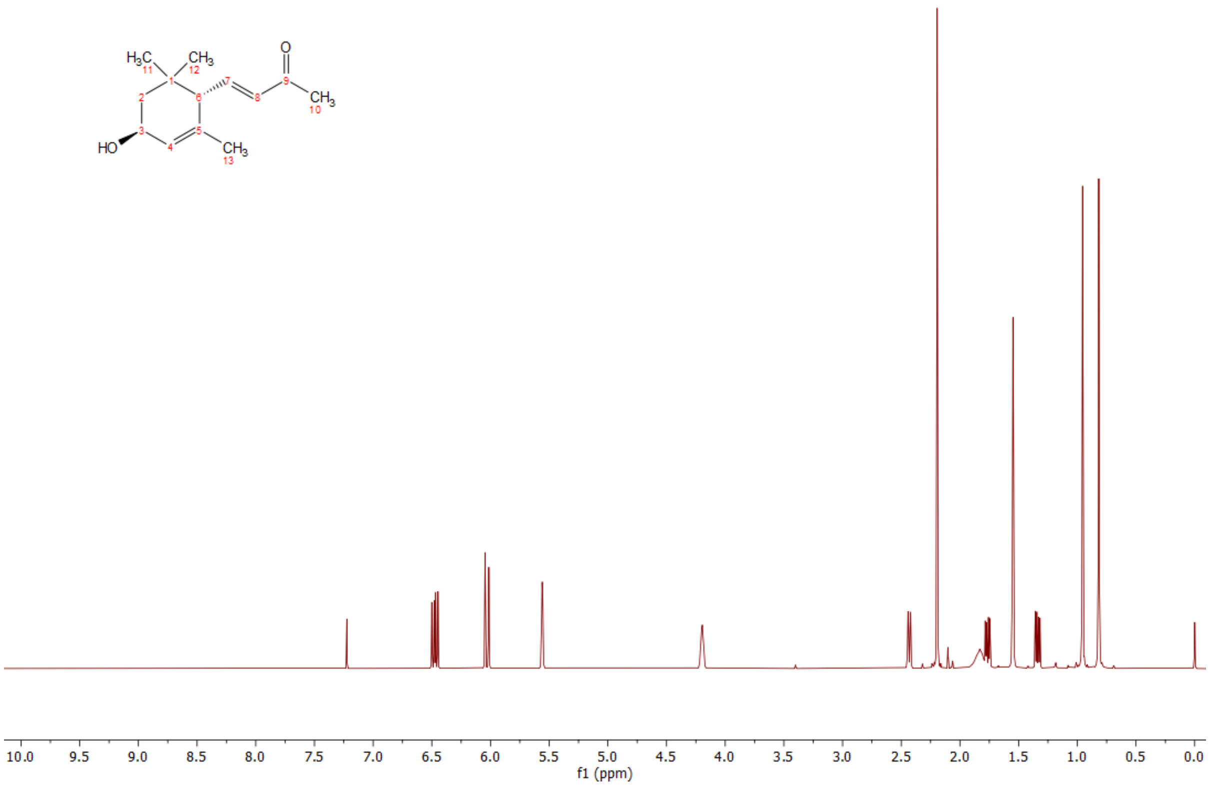

6c – ^{13}C NMR (126 MHz, $CDCl_3$)

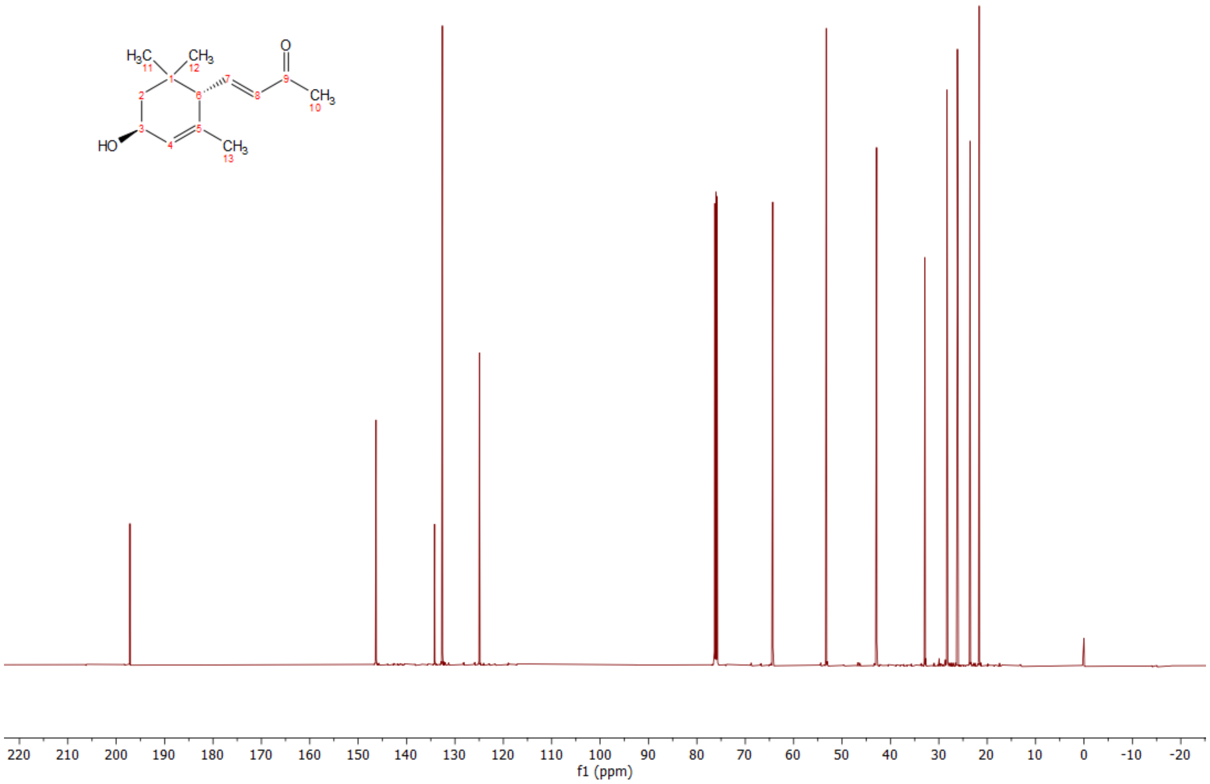

6d – ^{1}H NMR (400 MHz, CDCl3)

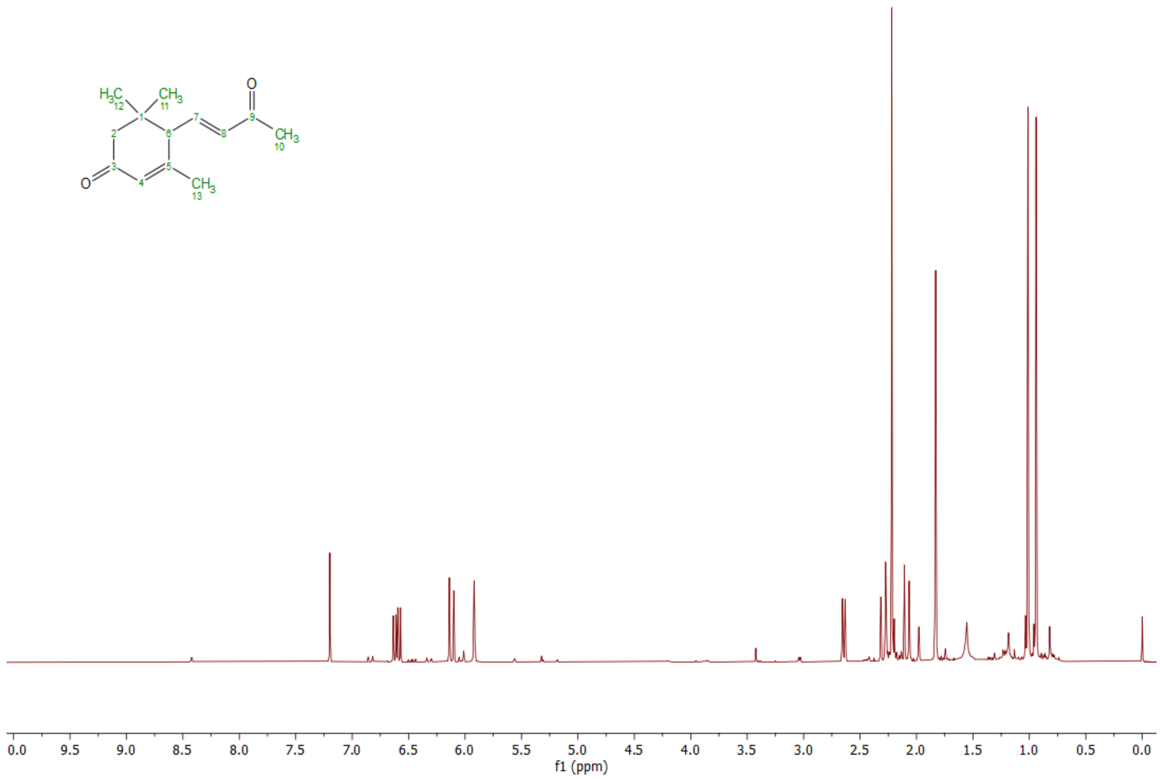

6d – ^{13}C NMR (101 MHz, CDCl3)

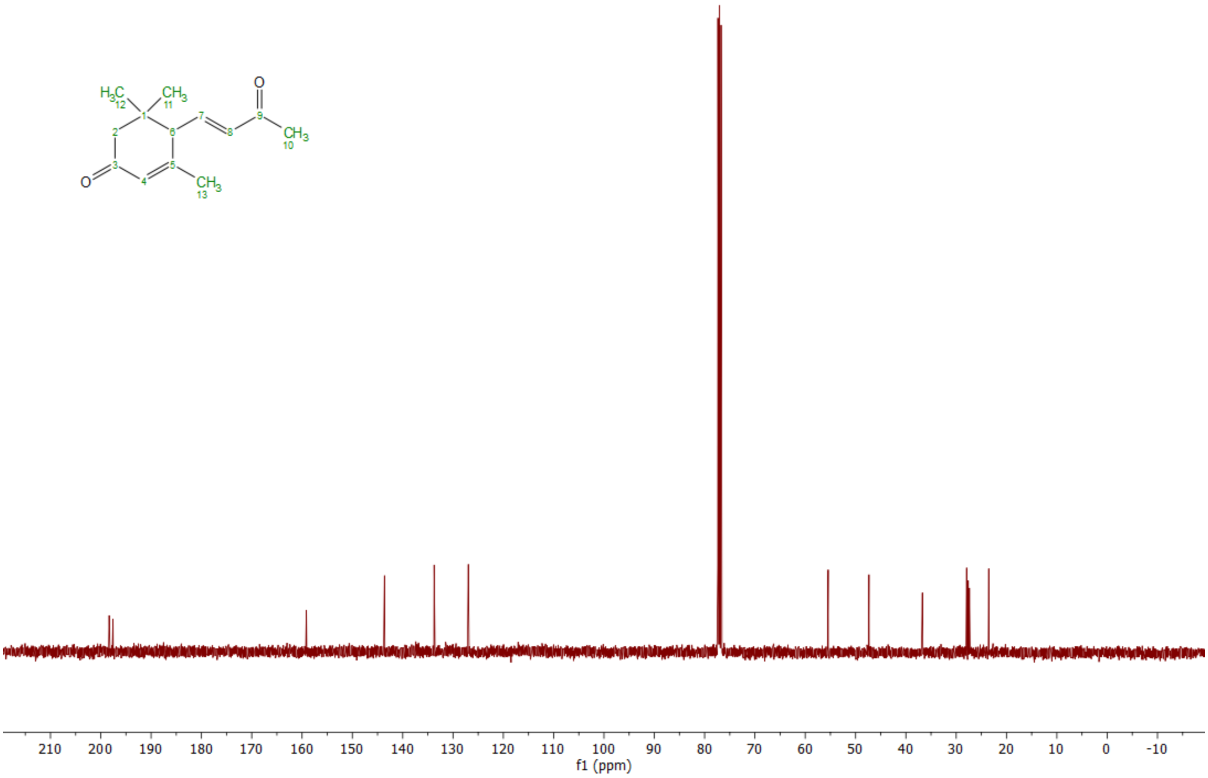

6e – ^{1}H NMR (500 MHz, $CDCl_3$)

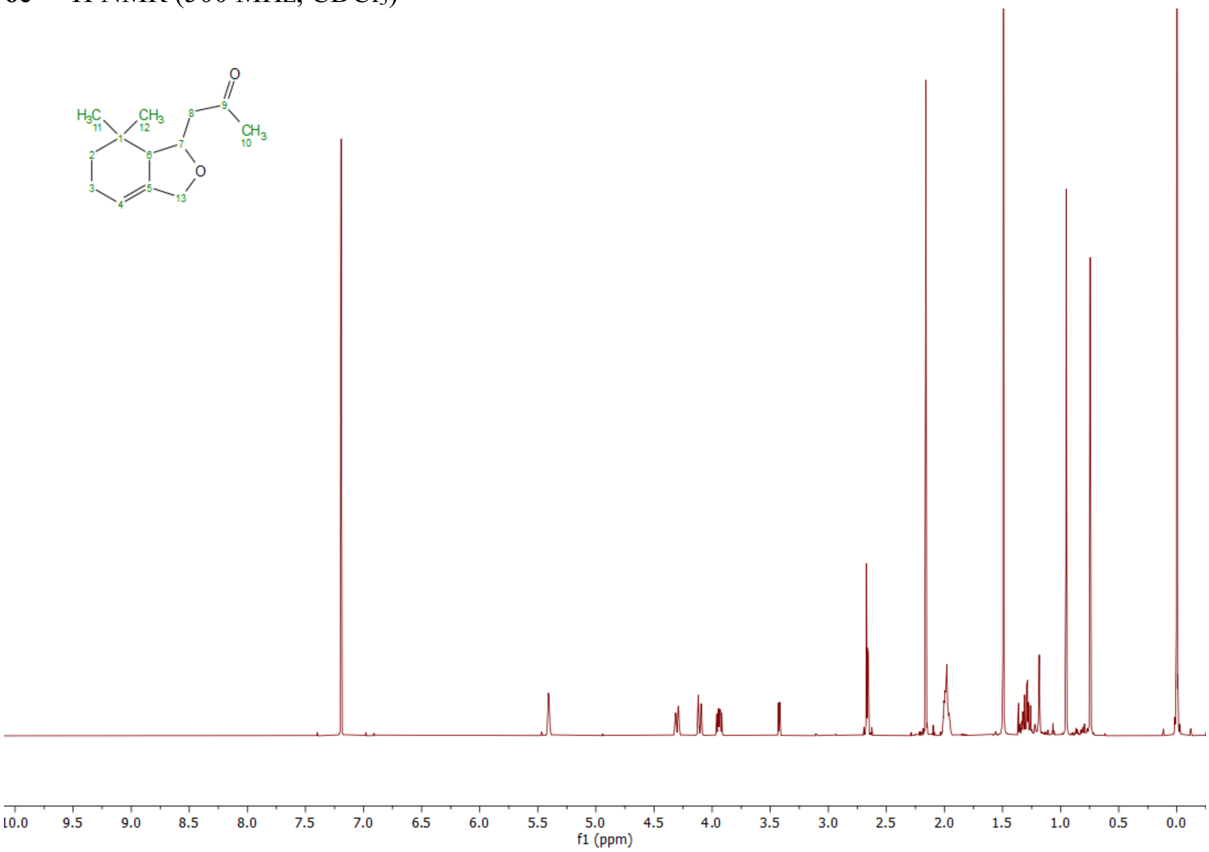

6e – ^{13}C NMR (126 MHz, $CDCl_3$)

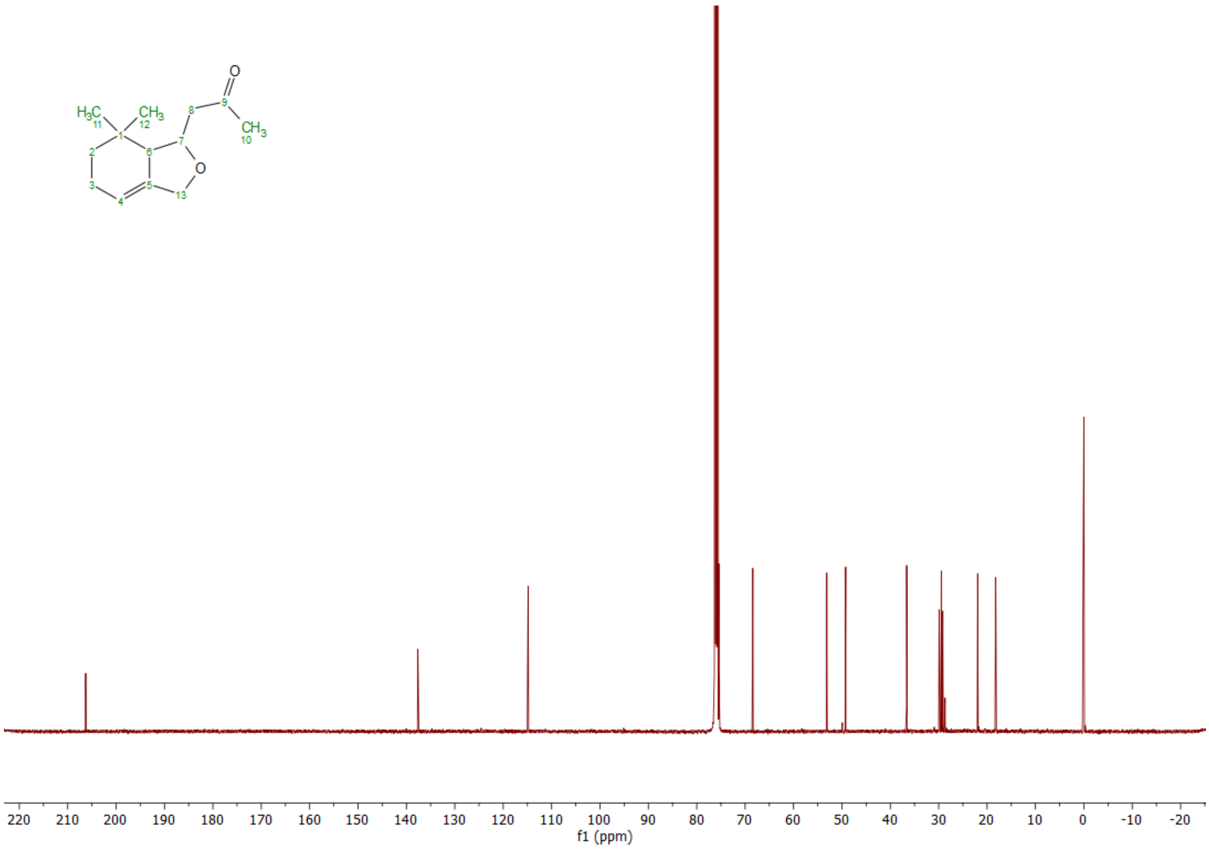

7 – ^{1}H NMR (400 MHz, CD_3OD)

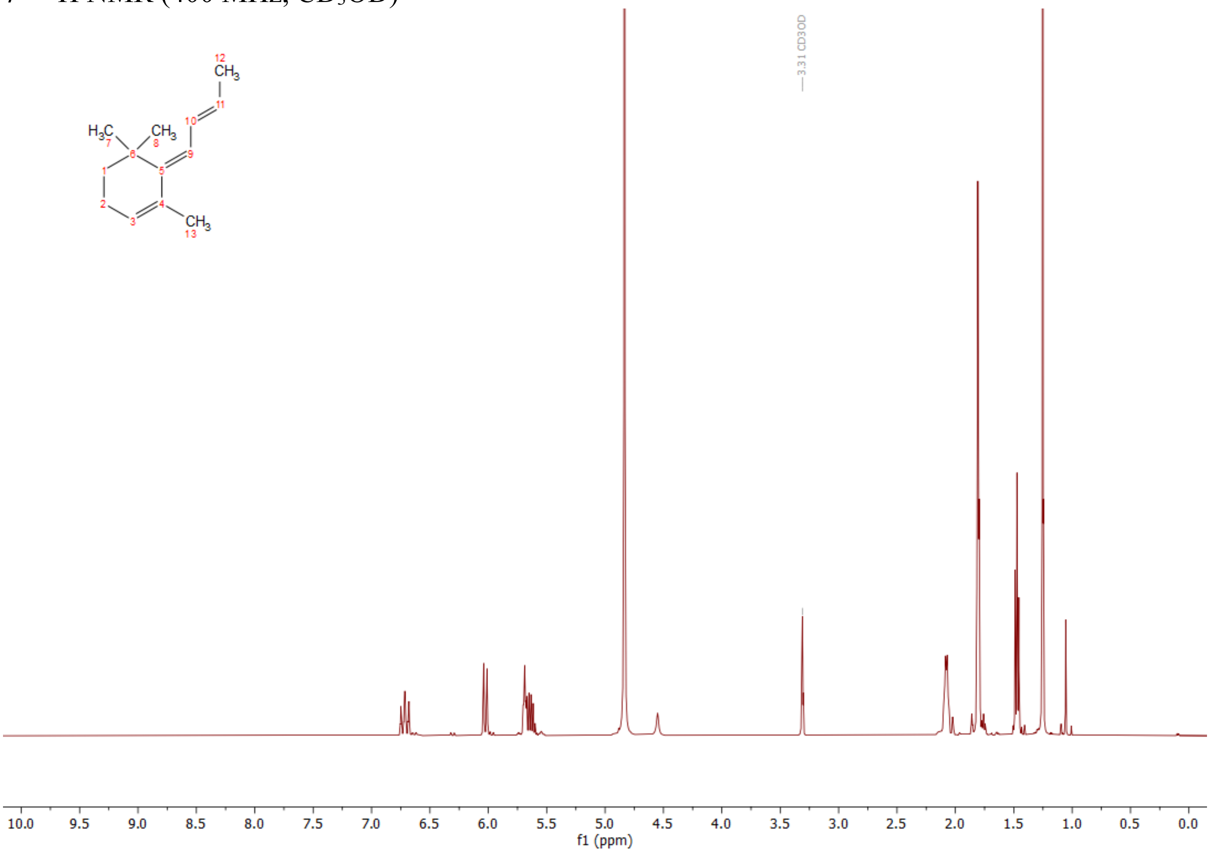

7 – ^{13}C NMR (101 MHz, CD_3OD)

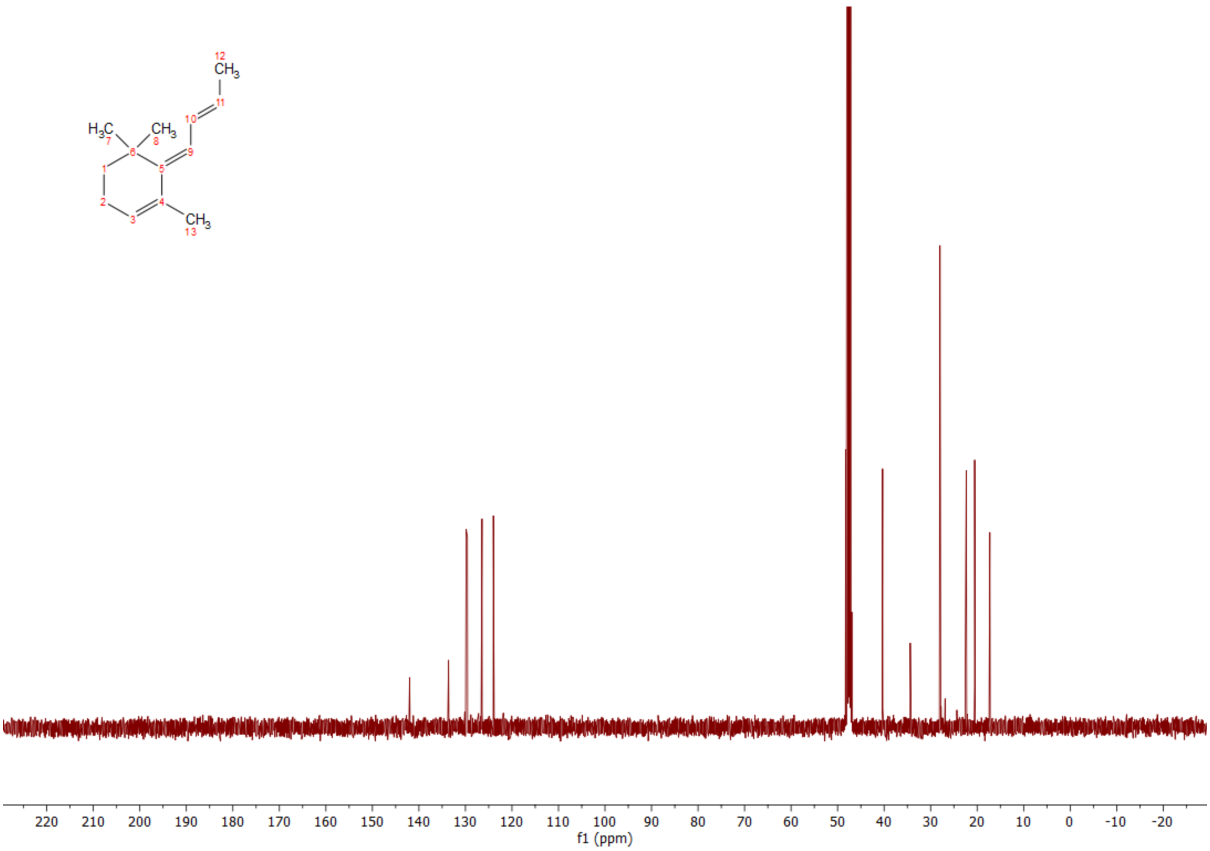

7a – ^{1}H NMR (500 MHz, CDCl$_3$)

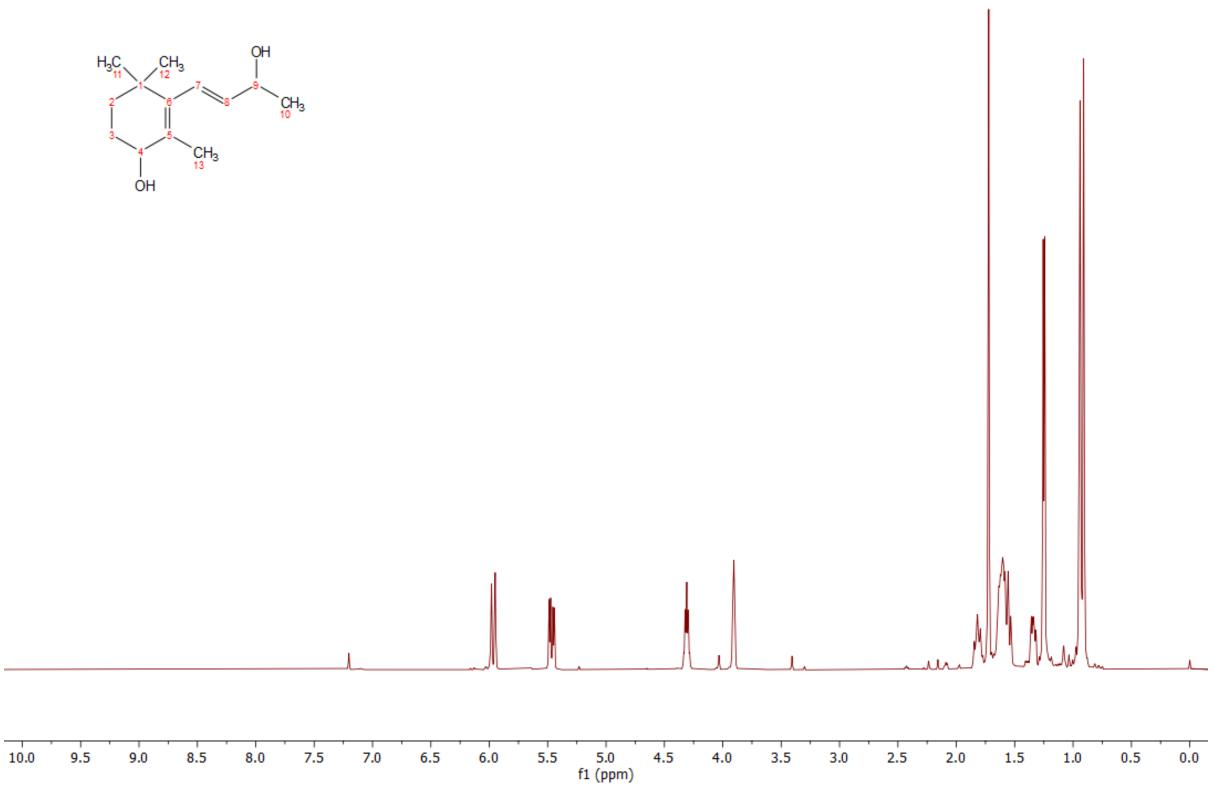

7a – ^{13}C NMR (126 MHz, CDCl$_3$)

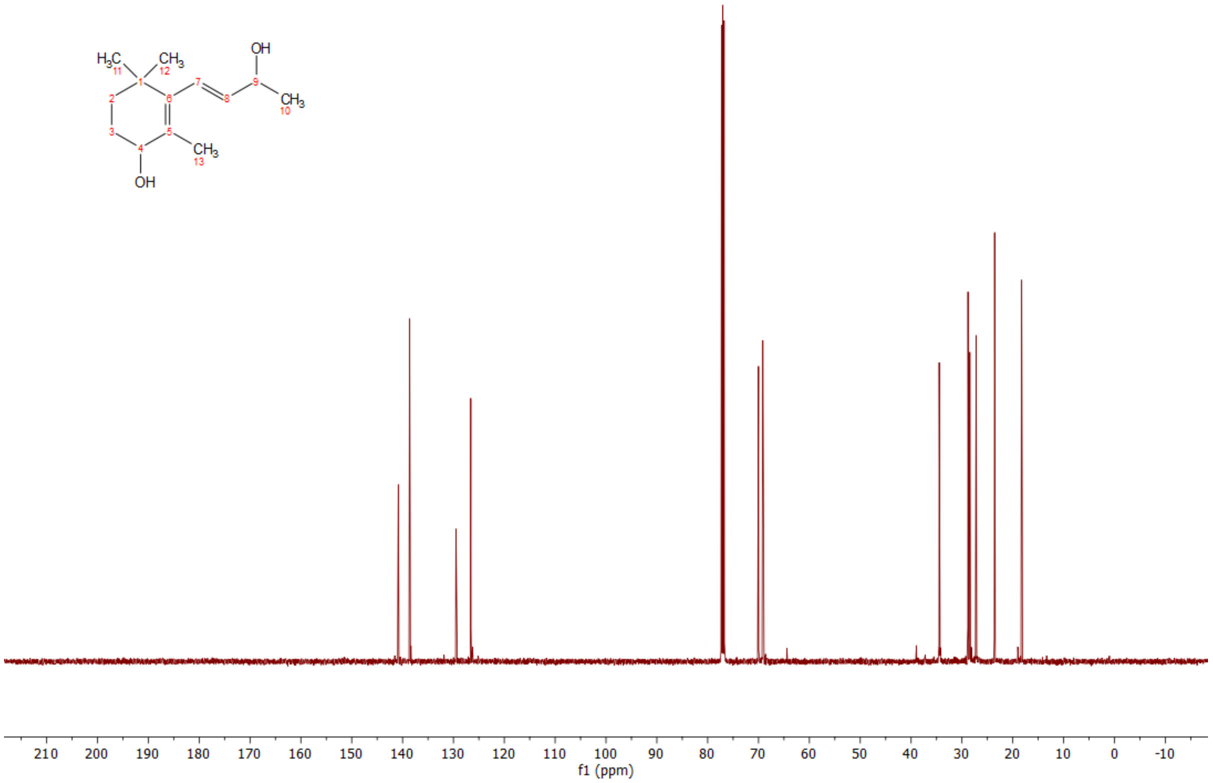

7b – ^{1}H NMR (400 MHz, $CDCl_3$)

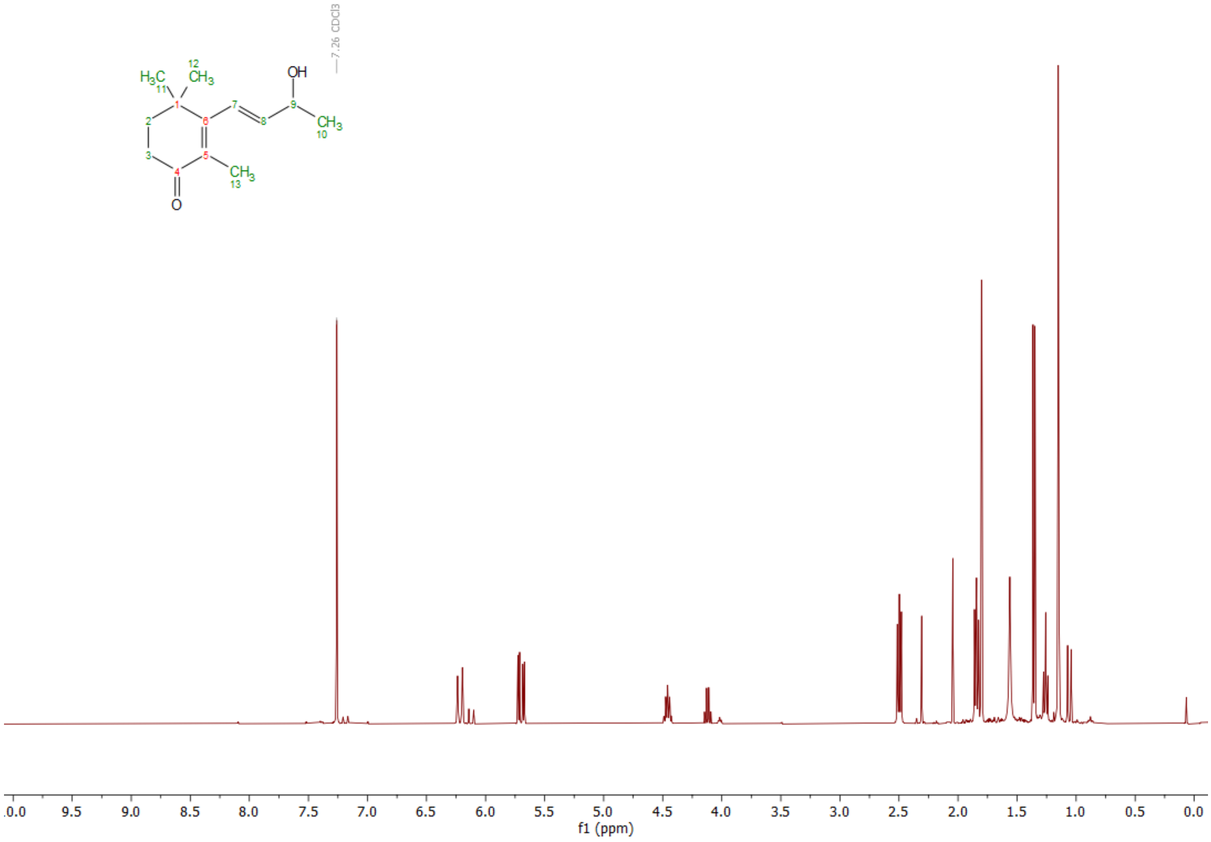

7b – ^{13}C NMR (101 MHz, $CDCl_3$)

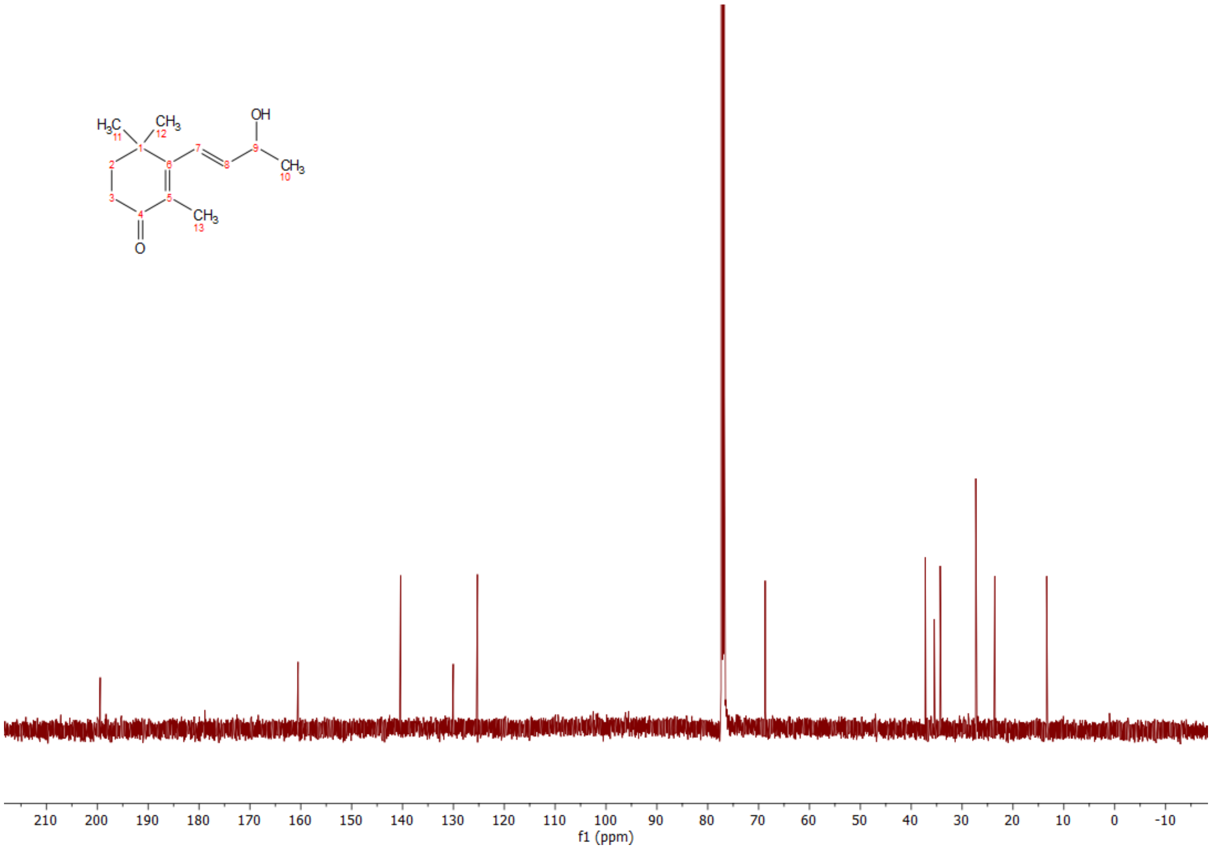

7c – ^{1}H NMR (400 MHz, $CDCl_3$)

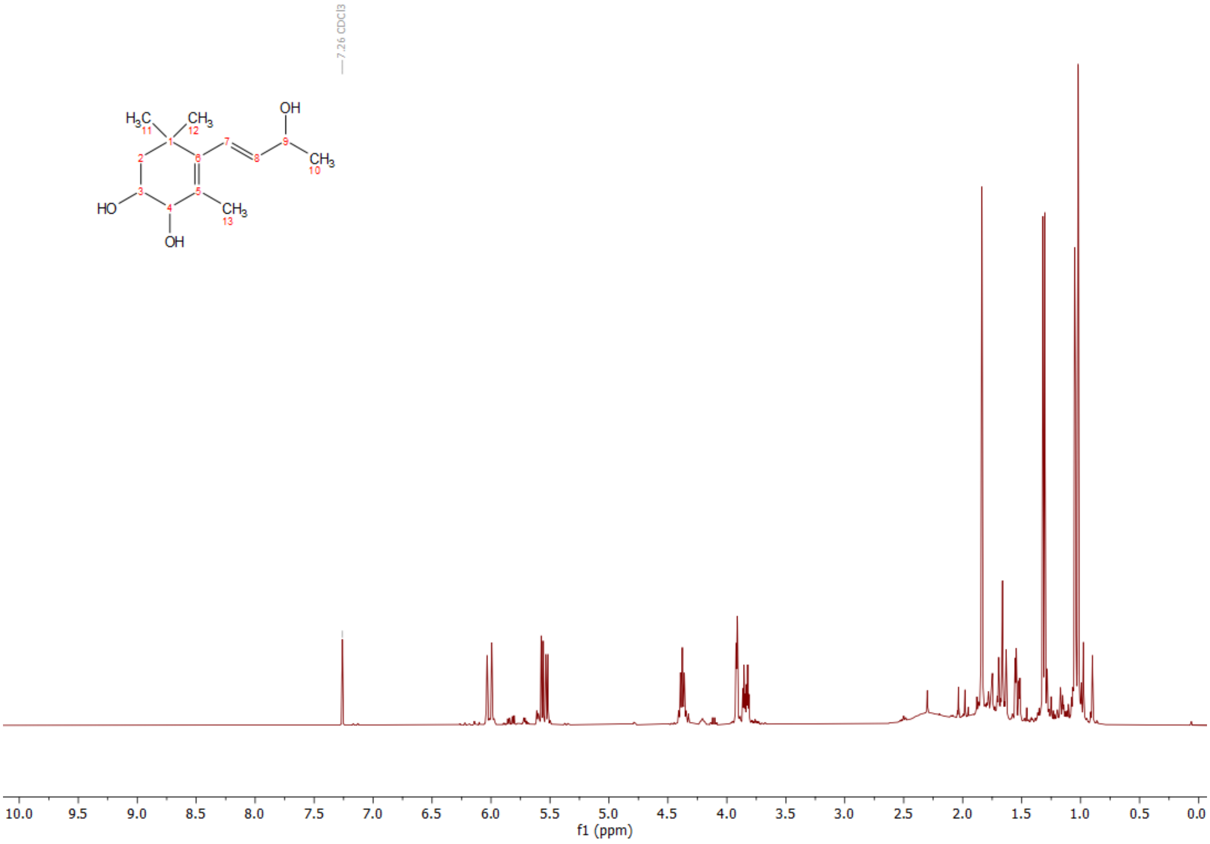

7c – ^{13}C NMR (101 MHz, $CDCl_3$)

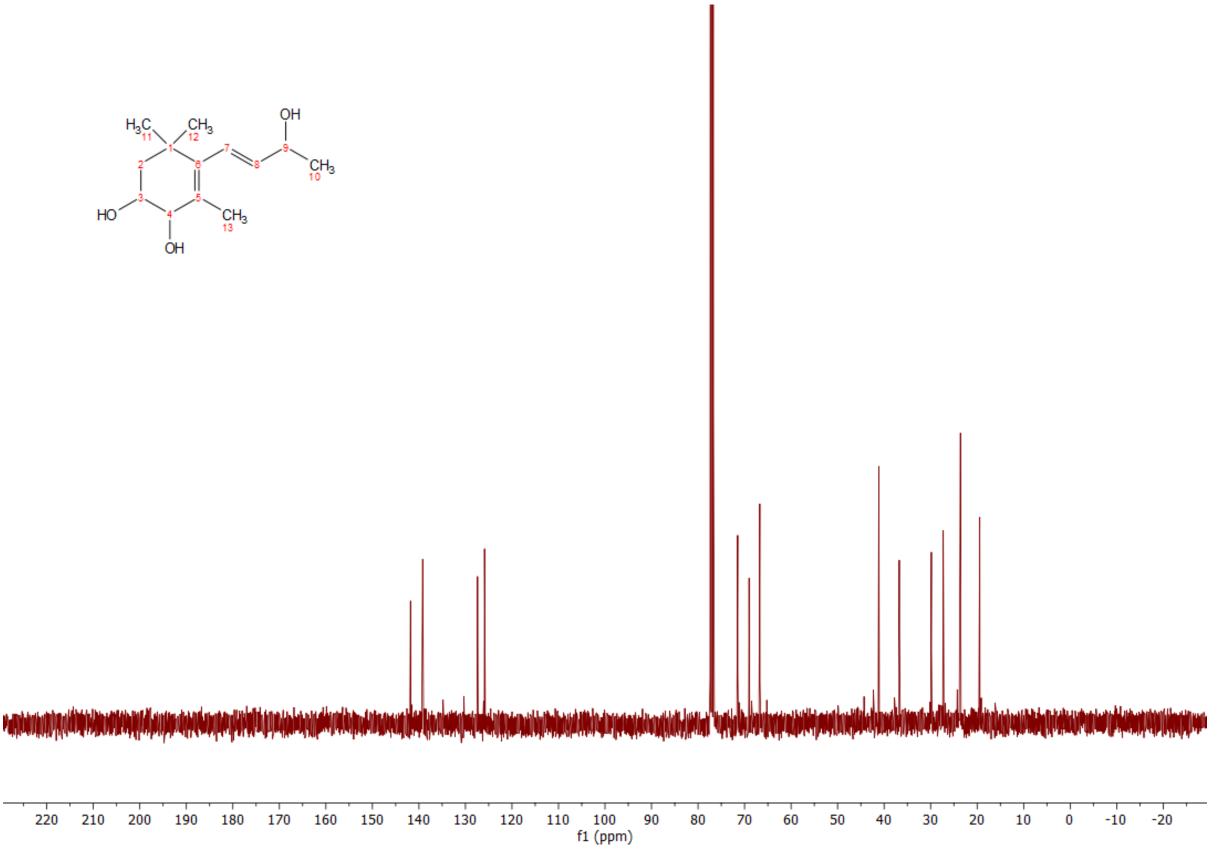

8 – ^{1}H NMR (500 MHz, CD_3OD)

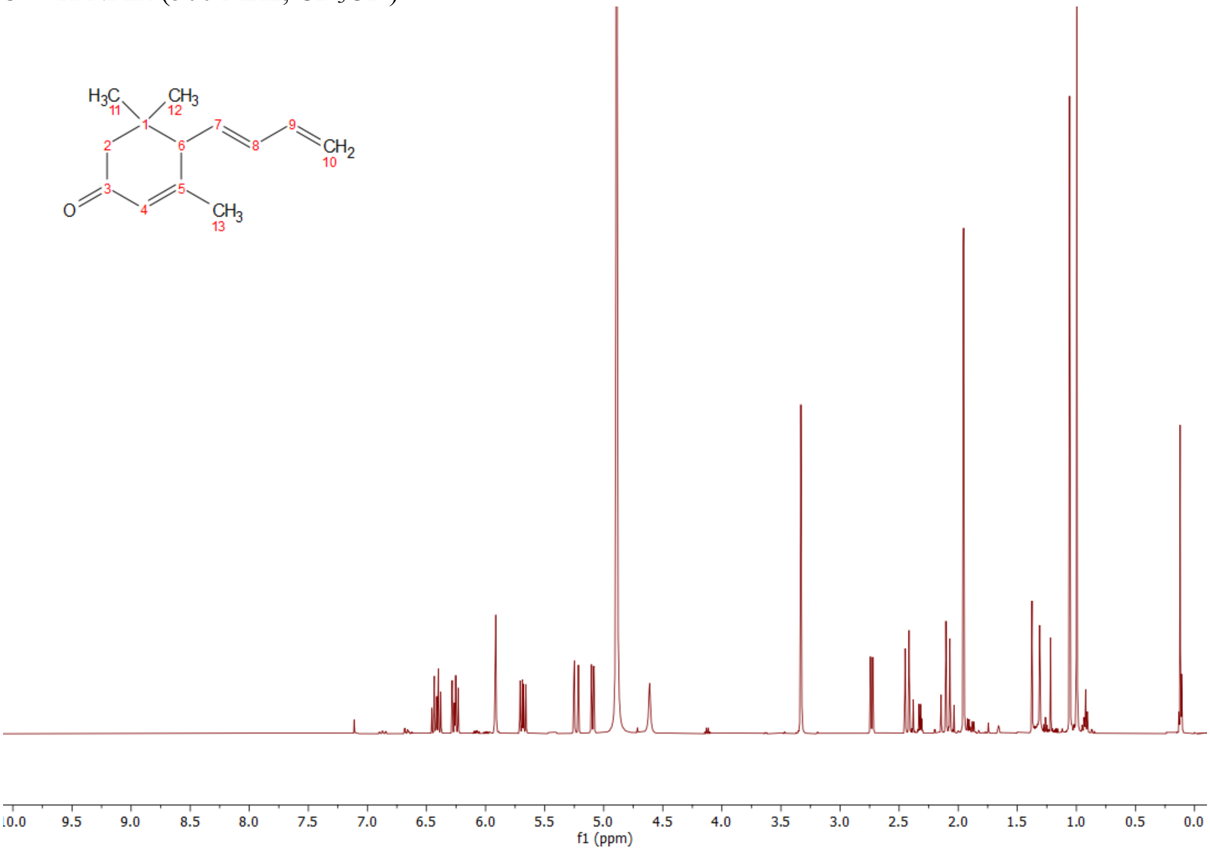

8 – ^{13}C NMR (126 MHz, CD_3OD)

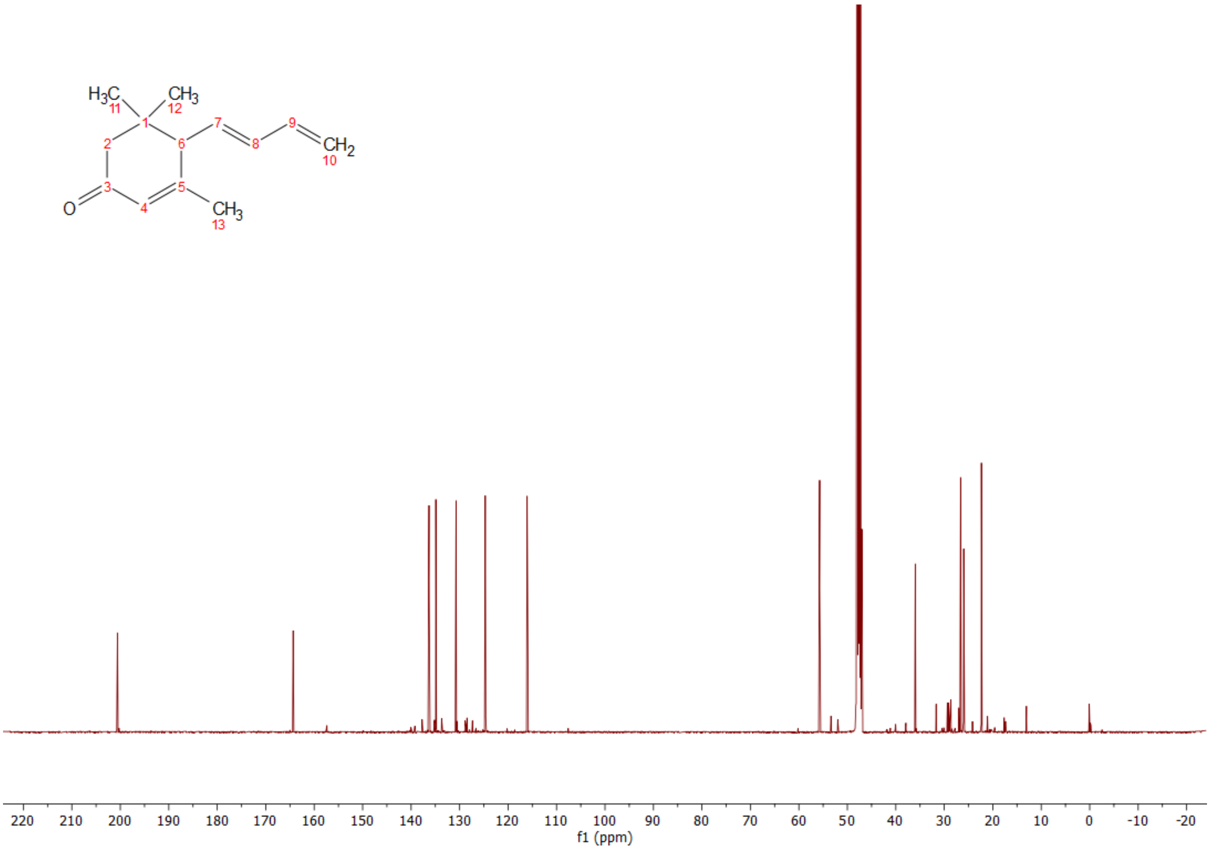

9–12 – ^{1}H NMR (400 MHz, $CDCl_3$)

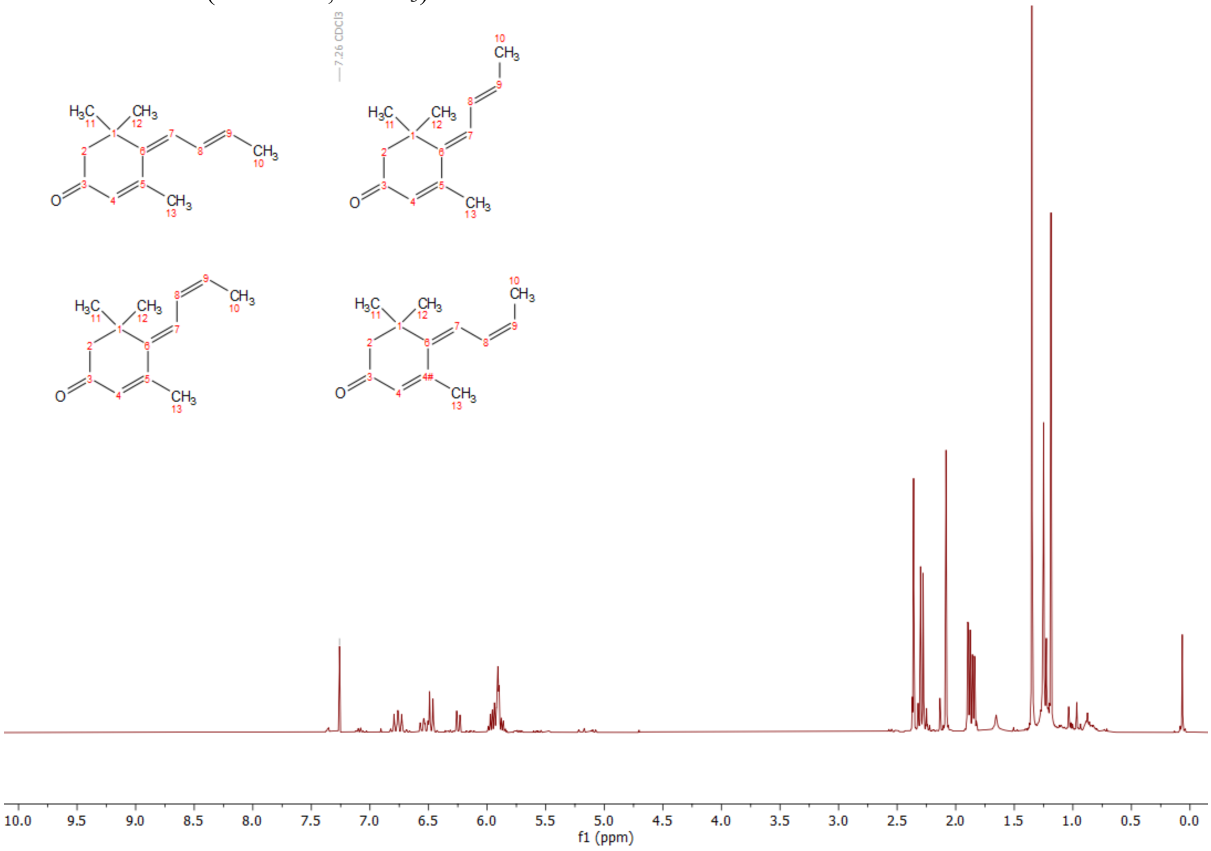

9–12 – ^{13}C NMR (126 MHz, $CDCl_3$)

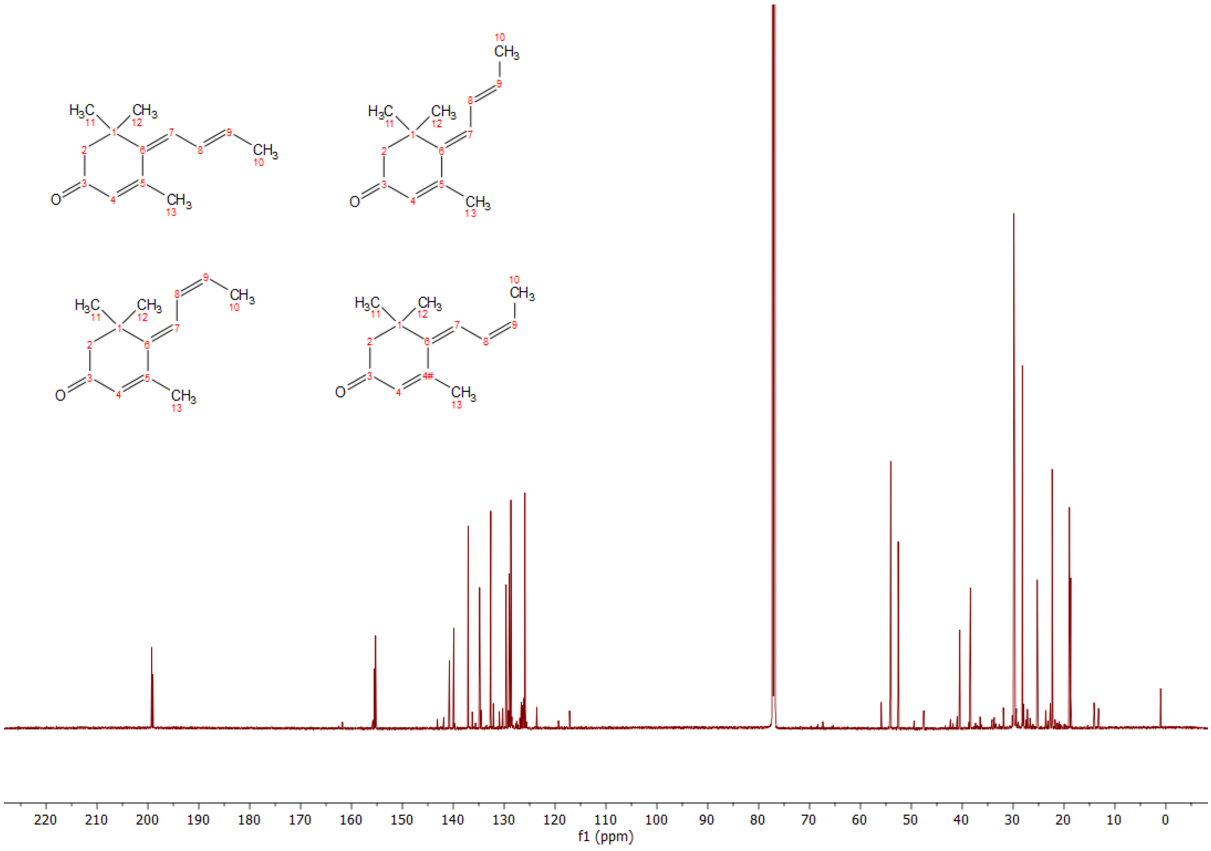

References

(1) Whitehouse, C. J. C.; Bell, S. G.; Tufton, H. G.; Kenny, R. J.; Ogilvie, L. C.; Wong, L. L. Evolved CYP102A1 (P450BM3) variants oxidise a range of non-natural substrates and offer new selectivity options. *Chem. Commun.* **2008**, 966–968. DOI: 10.1039/b718124h.

(2) Omura, T.; Sato, R. The Carbon Monoxide-Binding Pigment of Liver Micorsomes. *J. Biol. Chem.* **1964**, *239* (7), 2370–2378. DOI: 10.1016/S0021-9258(20)82244-3.

(3) Ma, M.; Bell, S. G.; Yang, W.; Hao, Y.; Rees, N. H.; Bartlam, M.; Zhou, W.; Wong, L. L.; Rao, Z. Structural Analysis of CYP101C1 from *Novosphingobium aromaticivorans* DSM12444. *Chembiochem* **2011**, *12* (1), 88–99. DOI: 10.1002/cbic.201000537.

(4) Clemente-Tejeda, D.; Lopez-Moreno, A.; Bermejo, F. A. Non-heme iron catalysis in C=C, C–H, and CH_2 oxidation reactions. Oxidative transformations on terpenoids catalyzed by Fe(bpmen)$(OTf)_2$. *Tetrahedron* **2013**, *69* (14), 2977–2986. DOI: 10.1016/j.tet.2013.02.013.

(5) Azzari, E.; Faggi, C.; Gelsomini, N.; Taddei, M. Transformation of α-Ionone and β-Ionone into α-Damascone and β-Damascone and β-Damascenone Using Allylsilane Chemistry. *J. Org. Chem.* **1990**, *55* (3), 1106–1108. DOI: 10.1021/jo00290a057.

(6) Litzenburger, M.; Bernhardt, R. Selective oxidation of carotenoid-derived aroma compounds by CYP260B1 and CYP267B1 from *Sorangium cellulosum* So ce56. *Appl. Microbiol. Biotechnol.* **2016**, *100* (10), 4447–4457. DOI: 10.1007/s00253-015-7269-7.

(7) Ohloff, G.; Rautenstrauch, V.; Schultee.Kh. Model Reactions for Biosynthesis of Compounds of Damascone Series and Their Synthetic Application. *Helv. Chim. Acta* **1973**, *56* (5), 1503–1513. DOI: 10.1002/hlca.19730560507.

(8) Schoch, E.; Benda, I.; Schreier, P. Bioconversion of α-Damascone by *Botrytis cinerea*. *Appl. Environ. Microbiol.* **1991**, *57* (1), 15–18. DOI: 10.1128/Aem.57.1.15-18.1991.

(9) Yorozu, K.; Takai, T.; Yamada, T.; Mukaiyama, T. A Novel Method for the Preparation of Acid-Sensitive Epoxides from Olefins with the Combined Use of Molecular-Oxygen and Aldoacetal Catalyzed by a Cobalt(II) Complex. *Bull. Chem. Soc. Jpn.* **1994**, *67* (8), 2195–2202. DOI: 10.1246/bcsj.67.2195.

(10) Behr, D.; Wahlberg, I.; Nishida, T.; Enzell, C. R. Tobacco Chemistry .47. (3*S*,6*R*,7*E*,9*R*)-4, 7-Megastigmadiene-3,9-Diol and (3*S**,6*R**,7*E*,9*S**)-4,7-Megastigmadiene-3,9-Diol. Two New nor-Carotenoids of Greek Tobacco. *Acta Chem. Scand. B:Org. Chem. Biochem.* **1978**, *32* (6), 391–394. DOI: 10.3891/acta.chem.scand.32b-0391.

(11) D'Abrosca, B.; DellaGreca, M.; Fiorentino, A.; Monaco, P.; Oriano, P.; Temussi, F. Structure elucidation and phytotoxicity of C13 nor-isoprenoids from *Cestrum parqui*. *Phytochemistry* **2004**, *65* (4), 497–505. DOI: 10.1016/j.phytochem.2003.11.018.

(12) Murakami, T.; Kishi, A.; Matsuda, H.; Hattori, M.; Yoshikawa, M. Medicinal foodstuffs. XXIV. Chemical constituents of the processed leaves of *Apocynum venetum* L.: Absolute stereostructures of apocynosides I and II. *Chem. Pharma. Bull.* **2001**, *49* (7), 845–848. DOI: 10.1248/cpb.49.845.

(13) Hall, E. A.; Bell, S. G. The efficient and selective biocatalytic oxidation of norisoprenoid and aromatic substrates by CYP101B1 from *Novosphingobium aromaticivorans* DSM12444. *RSC Adv.* **2015**, *5* (8), 5762–5773. DOI: 10.1039/c4ra14010a.

(14) Englert, G. C-13-Nmr - Study of Cis-Trans Isomeric Vitamins-a, Carotenoids and Related Compounds. *Helv. Chim. Acta* **1975**, *58* (8), 2367–2390. DOI: 10.1002/hlca.19750580817.

(15) Hutchins, R. O.; Learn, K.; Eltelbany, F.; Stercho, Y. P. Aminoborohydrides as Reducing Agents .1. Sodium (Dimethylamino)Borohydrides and (Tert-Butylamino)Borohydrides as Selective Reducing Agents. *J. Org. Chem.* **1984**, *49* (13), 2438–2443. DOI: 10.1021/jo00187a028.

(16) Ide, H.; Toki, S. Metabolism of β-Ionone - Isolation, Characterization and Identification of Metabolites in Urine of Rabbits. *Biochem. J.* **1970**, *119* (2), 281–287. DOI: 10.1042/bj1190281.

(17) Uchiyama, T.; Miyase, T.; Ueno, A.; Usmanghani, K. Terpenic Glycosides from *Pluchea indica*. *Phytochemistry* **1989**, *28* (12), 3369–3372. DOI: 10.1016/0031-9422(89)80349-8.

(18) Takazawa, O.; Tamura, H.; Kogami, K.; Hayashi, K. New Synthesis of Megastigma-4,6,8-Trien-3-Ones, 3-Hydroxy-β-Ionol, 3-Hydroxy-β-Ionone, 5,6-Epoxy-3-Hydroxy-β-Ionol, and 3-Oxo-α-Ionol. *Bull. Chem. Soc. Jpn.* **1982**, *55* (6), 1907–1911. DOI: 10.1246/bcsj.55.1907.
